# Precision phenotyping across the life cycle to validate and decipher drought-adaptive QTLs of wild emmer wheat (*Triticum turgidum* ssp. *dicoccoides*) introduced into elite wheat varieties

**DOI:** 10.3389/fpls.2022.965287

**Published:** 2022-10-12

**Authors:** Madita Lauterberg, Yehoshua Saranga, Mathieu Deblieck, Christian Klukas, Tamar Krugman, Dragan Perovic, Frank Ordon, Andreas Graner, Kerstin Neumann

**Affiliations:** ^1^Leibniz Institute of Plant Genetics and Crop Plant Research (IPK), Gatersleben, Germany; ^2^The Robert H. Smith Institute of Plant Sciences and Genetics in Agriculture, The Hebrew University of Jerusalem, Rehovot, Israel; ^3^Institute for Resistance Research and Stress Tolerance, Julius Kühn-Institute, Quedlinburg, Germany; ^4^Digitalization in Research and Development (ROM), BASF SE, Ludwigshafen am Rhein, Germany; ^5^Institute of Evolution, University of Haifa, Haifa, Israel

**Keywords:** high-throughput phenotyping, wild emmer wheat, near-isogenic lines, drought resilience, stay green, stay-green effect

## Abstract

Drought events or the combination of drought and heat conditions are expected to become more frequent due to global warming, and wheat yields may fall below their long-term average. One way to increase climate-resilience of modern high-yielding varieties is by their genetic improvement with beneficial alleles from crop wild relatives. In the present study, the effect of two beneficial QTLs introgressed from wild emmer wheat and incorporated in the three wheat varieties BarNir, Zahir and Uzan was studied under well-watered conditions and under drought stress using non-destructive High-throughput Phenotyping (HTP) throughout the life cycle in a single pot-experiment. Plants were daily imaged with RGB top and side view cameras and watered automatically. Further, at two time points, the quantum yield of photosystem II was measured with a top view FluorCam. The QTL carrying near isogenic lines (NILs) were compared with their corresponding parents by *t*-test for all non-invasively obtained traits and for the manually determined agronomic and yield parameters. Data quality of phenotypic traits (repeatability) in the controlled HTP experiment was above 85% throughout the life cycle and at maturity. Drought stress had a strong effect on growth in all wheat genotypes causing biomass reduction from 2% up to 70% at early and late points in the drought period, respectively. At maturity, the drought caused 47–55% decreases in yield-related traits grain weight, straw weight and total biomass and reduced TKW by 10%, while water use efficiency (WUE) increased under drought by 29%. The yield-enhancing effect of the introgressed QTLs under drought conditions that were previously demonstrated under field/screenhouse conditions in Israel, could be mostly confirmed in a greenhouse pot experiment using HTP. Daily precision phenotyping enabled to decipher the mode of action of the QTLs in the different genetic backgrounds throughout the entire wheat life cycle. Daily phenotyping allowed a precise determination of the timing and size of the QTLs effect (s) and further yielded information about which image-derived traits are informative at which developmental stage of wheat during the entire life cycle. Maximum height and estimated biovolume were reached about a week after heading, so experiments that only aim at exploring these traits would not need a longer observation period. To obtain information on different onset and progress of senescence, the CVa curves represented best the ongoing senescence of plants. The QTL on 7A in the BarNir background was found to improve yield under drought by increased biomass growth, a higher photosynthetic performance, a higher WUE and a “stay green effect.”

## Introduction

Wheat is one of the three most important staple foods worldwide and is consumed daily in the form of baked goods or pasta. Baked goods such as bread and flat bread are an important source of carbohydrates and protein for a large part of the human population (Shiferaw et al., 2013). The ongoing climate change threatens wheat yields, as with every degree of temperature, world wheat production decreases by 6% (Asseng et al., 2015). In 2020 the global wheat production was 760 million tons according to the FAOSTAT (2020), a warming of 1.5°C would mean a loss of 68 million tons. As the climate warms, droughts or dry and hot conditions are expected to become the norm by mid-century in major growing regions such as parts of Europe, the USA and Canada, and wheat production will accordingly fall below its long-term average (Leng and Hall, 2019; Toreti et al., 2019).

Drought is an extreme, prolonged condition in which less water or precipitation is available than is necessary for the plants’ needs. Due to the parched soil, transpiration needs are not met, so less water and nutrients can be taken up by mass flow or diffusion and development processes are severely impaired (Barzana et al., 2020). Photosynthesis requires CO_2_ as well as water and light. CO_2_ flows through the stomata into the mesophyll at a rate described by stomatal conductance, which is related to turgor pressure and osmotic potential (Buckley and Mott, 2013). Plants close their stomata with increasing drought stress to reduce water loss. If dry conditions prevail, water is lost through the stomata openings due to physical compensation. The osmotic pressure within the plant cell decreases and the resulting osmotic stress, a disturbance of the ion balance, damages the cell membrane and large molecules. Strategies to osmotically adjust or open stomata regulate intercellular solute levels under water limitation, promoting maintenance of turgor and integrity of metabolic functions (Blum, 2017).

In Mediterranean climates, of which Israel is one, water deficit and high temperatures are common during the final stage of wheat growth, hence the grain filling phase is mainly affected (Saranga et al., 2008). Escape from drought is a common strategy to prevent the effects of such terminal drought. The strategy involves rapid plant development with a high metabolic rate. The stomata are open to allow the necessary high gas exchange. This leads to a moderate but effective photosynthetic rate, low water use efficiency (WUE) and rapid expansion and division of cells (Wang et al., 2017).

Phenotyping is known to be a time-consuming and partly subjective procedure. Non-invasive high-throughput phenotyping (HTP) offers a precise and rapid way to study genotypes in an objective and standardized manner (Chen et al., 2014). It can be carried out in the field or in the greenhouse, with greenhouse experiments under controlled conditions being particularly suitable for climate change scenarios such as drought stress (Langstroff et al., 2022). Modern phenotyping technology provides better experimental opportunities to identify key loci and mechanisms for the complex stress response (Langridge and Reynolds, 2021). Precision phenotyping has allowed a deep characterisation of individual drought tolerance components in barley with high phenotypic data quality even under drought and thus the breakdown of their genetic architecture (Neumann et al., 2015; Dhanagond et al., 2019; Pham et al., 2019).

However, up to now, HTP experiments have only been conducted until about flowering time. Insight into the senescence phase could previously be obtained by mobile field phenotyping from flowering to final maturity (Christopher et al., 2016) during some days between anthesis and final maturity (Kipp et al., 2014; Christopher et al., 2016). Nevertheless, the entire life cycle from plant establishment to final maturity has not yet been assessed by non-destructive phenotyping.

Wild species represent an important genetic resource to identify beneficial alleles from landraces and wild relatives and incorporate these into modern varieties (Mascher et al., 2019). Wild emmer wheat is the ancestor of bread and durum wheat. It is well adapted to the dry climate of the levant and thus represents a valuable source of genetic diversity to improve drought resilience (Nevo and Beiles, 1989; Peleg et al., 2005; Krugman et al., 2018).

In a recombinant inbred line (RIL) population derived from a cross between durum wheat (cv. Langdon) and wild emmer wheat (acc. G18-16), two beneficial QTLs originating from wild emmer wheat were identified (Peleg et al., 2009; Fatiukha et al., 2021). A QTL on chromosome 2B confers higher grain yield under drought stress and control conditions and a QTL on chromosome 7A confers higher total and spike dry matter under drought stress, referred to hereafter as higher productivity (Merchuk-Ovnat et al., 2016a). Near isogenic lines (NILs) carrying these QTL regions have been developed for the 2B and 7A QTLs by marker assisted breeding backcross procedure to three Israeli cultivars. The advantageous effect on yield components in the elite cultivar background has been confirmed previously under well-watered and drought conditions (Merchuk-Ovnat et al., 2016a,b).

In the current study, we investigated the NILs carrying the two wild emmer QTLs along with the parental cultivars in an HTP experiment using the previously established protocol to simulate drought stress (Dhanagond et al., 2019). For the first time, a HTP experiment captured the whole plant life cycle. The study aimed to validate the QTL effects during drought stress observed in the field/screenhouse, in a pot experiment and to specify the spatio-temporal effects of wild emmer wheat QTLs on growth and drought resilience across the entire life cycle.

## Materials and methods

### Plant material

A marker-assisted backcross program was employed for the introgression of the wild donor (*T. turgidum* ssp. *dicoccoides*, acc. G18-16) alleles in selected QTL regions into durum and bread wheat cultivar., as described previously by Merchuk-Ovnat et al. (2016a). All the recurrent parents were elite Israeli cultivars, widely used commercially and well adapted to the Israeli semi-arid condition. The parental lines have very similar heading times, with only few days differences (Merchuk-Ovnat et al., 2016a). We studied three Near-Isogenic Lines (NIL) at a stage of BC_3_F_7_ and their recurrent parental cultivars: NIL-B-7A-2 and NIL-Z-7A-5 both carrying the 7A QTL with a size of 46 cM in the background of bread wheat cultivars BarNir and Zahir, respectively, and NIL-U-2B-3 carrying the 2B QTL comprises 43,5 cM the background of durum wheat cultivar Uzan (Table 1). In a new genetic map generated by 15 K SNP array (TraitGenetics, Gatersleben) the 7A QTL is designated as *QVegdm.huj. uh-7A* and the 2B QTL is designated as *QGy.huj. uh-2B.1* (Fatiukha et al., 2021). Furthermore, the size of the QLT on chromosome B7A in BarNir measures 115Mbp (Deblieck et al., 2020).

**Table 1:** Overview of used plant material.

Name	Generation	Recurrent parents	Location of QTL introgression	Associated traits	Flanking markers
BarNir *T. aestivum*	Israeli cultivar				
NIL-B-7A-2 *T. aestivum*	BC_3_F_7_	BarNir	7A	Total & Spike Dry Matter under Drought	Xgwm60, Xwmc422
Zahir *T. aestivum*	Israeli cultivar				
NIL-Z-7A-5 *T. aestivum*	BC_3_F_7_	Zahir	7A	Total & Spike Dry Matter under Drought	Xgwm60, Xwmc596
Uzan *T. durum*	Israeli cultivar				
NIL-U-2B-3 *T. durum*	BC_3_F_7_	Uzan	2B	Grain Yield & Harvest Index	Xgwm1128, Xgwm1177

Modified after (Merchuk-Ovnat et al., 2016a).

The name of the NIL is derived from the first letter of the parental variety, the number of the chromosome containing the introgression, and the line number.

These lines were previously evaluated in two consecutive years, under contrasting water regimes in a field/screenhouse experiment in Israel and were found advantageous under drought for the respective traits (Merchuk-Ovnat et al., 2016a).

### HTP system

The High-throughput Phenotyping (HTP) system (LemnaTec-Scanalyzer 3D) used in the current study is installed in an environmentally controlled greenhouse at IPK Gatersleben (51°49′23″ N, 11°17′13″ E, altitude 112 m). On this system, each plant is transported by conveyor belts to the imaging chambers equipped with top and side view RGB and fluorescence cameras, where the lifter allows imaging from different angles in side view. The balance-watering station enables controlled watering and thereby defined drought setups. The system has been upgraded with a chlorophyll fluorescence camera (FluorCam) from PSI to measure photosynthetic performance from top view (Tschiersch et al., 2017).

### HTP experiment

All wheat lines were phenotyped on the HTP platform under contrasting water supply with 10 biological replicates per line in each treatment. The HTP experiment took place from July 2019 to November 2019 and covered the entire life cycle of the wheat plants from sowing until maturity. Seeds were provided by Prof. Y. Saranga, the Hebrew University of Jerusalem, Israel. To ensure the presence of one plant in each pot even, two seeds per pot were sown and thinned after germination to leave one seedling per pot. Each pot (18.5 cm height x 14.9 cm diameter) was filled in with Klasman substrate no. 2 and supplemented with 7 g fertilizer, containing 19% total nitrogen, 9% P_2_O_5,_ and 10% K_2_O. Below each pot is a container to collect water in case not all water during automated watering can be fully absorbed immediately. Since the growing conditions are not homogenous within a greenhouse, all pots were randomized by the modus “Random 208,” which exchanges randomly positions of 208 plants with the position of another plant, included in the operating software of the commercial system, several times a week. Plants were imaged and watered daily, imaging was performed from three side view angles (0, 45 and 90°) and top view. In the well-watered treatment, plants were always watered to 90% plant available water content according to Dhanagond et al. (2019). The day length of supplementary greenhouse lights was set to 15 h per day during the whole experiment. The chosen temperature and watering setup during the HTP experiment aimed to mimic the Israeli field situation where temperatures increase over time and a drought period is slowly establishing and progressing. However, it should be noted that technical limitations of the greenhouse climatization prevent to match the exact temperatures as in Merchuk-Ovnat et al. (2016a) or the daily temperature gradients. Here, maximal temperatures of around 47 degrees were achieved in the field/screenhouse, which is not feasible in a greenhouse. Until 30 days after sowing (DAS), plants grew without any stress for plant establishment. The temperature during this first phase was 12°C at night and 16°C during the day. To induce drought stress, the irrigation of the plants in stress treatment was reduced to 30% plant available water content on DAS 31. This level that does not cause plant wilting or visual stress symptoms, but results in reduced growth and is therefore considered a mild drought level. With the onset of drought, the temperature was increased to 20°C during the day and 16°C at night. At DAS 62 (about a week after heading), a further temperature increase was made to 24°C during the day and 20°C at night. From DAS 64 onwards, irrigation was further reduced to 20% plant available water content to induce severe drought in the grain filling phase based on the barley threshold from Dhanagond et al. (2019). Drought level and temperature regime were persisted until maturity.

Measurements of chlorophyll fluorescence were made with the FluorCam at two time points during severe drought stress (DAS 69 and 74). Regular plant recordings and irrigation were carried out at night, starting at midnight. The first saturating light flash (4,000 μmol/m^2^/s) is used to measure the working efficiency of photosystem two under strong light influence (800μmo/m^2^/s), shortly followed by a second light flash to measure the quantum yield after a lower light intensity (80 μmol/m^2^/s) (Grieco et al., 2020). If the quantum yields of the second and the first light flash are put in relation to each other, one can measure how the photosystem can adapt to the changing light flashes, i.e., what plasticity it possesses.

Several additional traits were measured during the HTP experiment. Number of tillers were counted at DAS 28 before the drought, at DAS 53 (about 3 weeks of mild drought) and at DAS 70 about one week after the onset of severe drought (Supplementary material 1). Heading time (BBCH55) was determined by visual inspection of the raw images, when half of the first developing ear protrudes from the flag leaf (Witzenberger et al., 1989). At DAS 67 flag leaf of the main tiller from each plant was measured for length and width at the widest point to calculate flag leaf area (length*width*0.75 = area). Then the distal half of the same flag leaf was sampled to determine osmolality (Osmotic Potential MPa; Table 2) using a vapor pressure osmometer (model 5520; Wescor Inc., Logan, UT, USA) as described by Merchuk-Ovnat et al. (2016a).

**Table 2:** Non-imaging traits measured before and at maturity.

Traits measured before maturity	Traits measured at maturity
TN DAS28	Main Ear Awn Length (cm)
TN DAS53	Ear Length (cm)
TN DAS70	Culm Length (cm)
Gain of TN DAS28 and 53	Peduncle length (cm)
Gain of TN DAS53 and 70	Last Internode length (cm)
Gain of TN DAS28 and 70	Plant height (cm)
BBCH55 in DAS	Number of Spikes
Flag Leaf Width (mm)	Number of fertile Spikes
Flag Leaf Length (cm)	Plant Biomass (g)
Flag Leaf Area (cm^2^)	Plant Grain Weight (g)
Osmotic Potential (MPa)	Plant Straw Weight (g)
QY-H DAS 69	Plant Harvest Index
QY-H DAS 74	Biomass WUE (g/l)
QY-L DAS 69	Plant TKW (g)
QY-L DAS 74	Plant Seed area (mm^2^)
QY-LH Ratio DAS 69	Plant Seed width (mm)
QY-LH Ratio DAS 74	Plant Seed length (mm)
	Plant Grain Number
	Grains per Ear
	Main Ear Spikelet Number
	Main Ear Grain Number
	Main Ear Grains per Spikelet
	Main Ear Biomass (g)
	Main Ear Grain Weight (g)
	Main Ear Straw (g)
	Main Ear Harvest Index
	Main Ear TKW (g)
	Main Ear Seed Area (mm^2^)
	Watersum(l)

TN, tiller number; QY-H, quantum yield of photosystem II under high-light and QY-L under low light; QY-LH, ratio of QY-L to QY-H; DAS, days after sowing.

At the ripening phase, plants were inspected and taken off the system for harvest when having reached full maturity. The first plants were removed from the system at DAS 96, while the last mature plants were removed at DAS 121. The day of final maturity, called final estimated biovolume (EB), was considered to be the day when, for the last time, seven out of ten biological replicas were still on the platform. As plant appearance only slowly changed in the ripening period, images were only taken every 2 days from DAS 96 onwards, while watering was still continued daily. At plant maturity, several growth and yield parameters were recorded manually (Table 2). We determined plant height (PH) without awns, culm length (CL) was from the soil surface to the base of the three first spikes, ear length, peduncle length and the length of the last internode below the flag leaf. In addition, the total number of spikes and the number of fertile spikes were counted. The above-ground plant biomass, the grain, and the straw weight were determined, and the harvest index (HI) was calculated. The main ear was harvested separately. Thousand kernel weight (TKW) was determined for the seeds of the main ear and for the seeds from the rest of the plant. Since the water sum for each pot is recorded by the system, the water use efficiency (WUE) for the entire biomass was calculated from the ratio of final biomass to water sum.

### Comparison of the HTP experiment with the screenhouse/field experiment

In order to evaluate to what extent, the data from the HTP studies correspond to the results in the two field/screenhouse experiments, the comparable traits were used: Culm Length (cm; CL), Days Planting to Heading (here referred to BBCH55), Grains per Ear, Grain Yield (g), Harvest Index, Osmotic Potential (Mia), Number of Spikes per Plant, TKW and Plant Biomass (g; Merchuk-Ovnat et al., 2016a). The absolute values with significance between the parents and the NIL were compared. Additionally, the average value of each individual NIL was compared to the corresponding parent in percentage (NIL/Parent*100).

### Image analysis

The image analysis was carried out with the Integrated Analysis Platform (IAP; Klukas et al. 2014). Due to the long observation time, the data set was more than two Terabyte and could initially not be handled by IAP. This problem was overcome with the release of IAP version 2.3.0. The FluorCam data were analyzed using the manufacturer’s software.

Our focus in image data processing was on parameters from the RGB camera: the EB [voxel], plant height (PH) [mm] and the mean color value (CVa) [hue] from the side view. This average color value is part of the HSV color space, which is described by the hue, saturation and value of a color. To simplify the analysis of colors, different hue values are combined in a 20-bin model. Based on this model, an average hue of 0.23 corresponds to an image of a green plant. The EB is calculated based on images from top view and three different angles of side view.


$$\begin{array}{l} \mathrm{Estimated Biovolume}\;\left[\mathrm{voxel}\right]\\ =\sqrt{\mathrm{average pixel}\;{\mathrm{side area}}^{2}*\mathrm{top}\;\mathrm{area}} \end{array}$$

### Statistical analysis

For the statistical analysis of the HTP experimental data, R Studio was used as described by Dhanagond et al. (2019). For the estimation of variance components and Best Linear Unbiased Estimations (BLUEs), the R package ASReml was used and consequently a mixed model with Residual Maximum Likelihood applied. In terms of plant establishment, all plants grew under identical conditions until DAS 31. Consequently, there was still no stress or control treatment, so all 20 biological replicates for each parent and each corresponding NIL were considered for subsequent data analysis. From DAS 31, the start of the mild drought treatment, the two treatments drought and well-watered were analyzed separately.

The outlier test was performed according to the Tukey method (Anscombe and Tukey, 1963). BLUE values were calculated for each day and treatment separately using the model *Y* = (*μ* + *G* + *e*), where *Y* is the vector of observed phenotypic values, *μ* is the intercept, *G* is the effect of genotype and *e* is the residual value for each plant. *μ* and *G* were treated as fixed effects.

The variance components were calculated using the mixed linear model *Y* = *G* + *e*, where *G* is the effect of genotype and e is that of residual. There is also the assumption that all effects are random effects. Since this is a single experiment, repeatability is calculated from the variance of the genotype the error variance and ten biological replicates.


$$R=\frac{v_{G}}{v_{G}+\frac{v_{e}}{nRep}}$$

*R* Repeatability

*V_G_* genotypic variance

*V_e_* error variance

*nRep* number of biological replicates.

Values for the EB were analyzed with the downscaling factor of 10^6^ during all calculations. Further, due to a management error, the FluorCam was forgotten to pull back and therefore blocked the view of the RGB top view camera from DAS 28 to 32. Therefore, these days were excluded for EB that is calculated from side and top view areas but could be analyzed for PH (from side view images) and side view CVa. As FluorCam measurement on the first time point before the onset of drought was not successful, we did not consider this day in the analysis and only used the data from DAS 69 and 74.

## Results

### High repeatability of biomass related traits under drought stress

**Figure 1:**
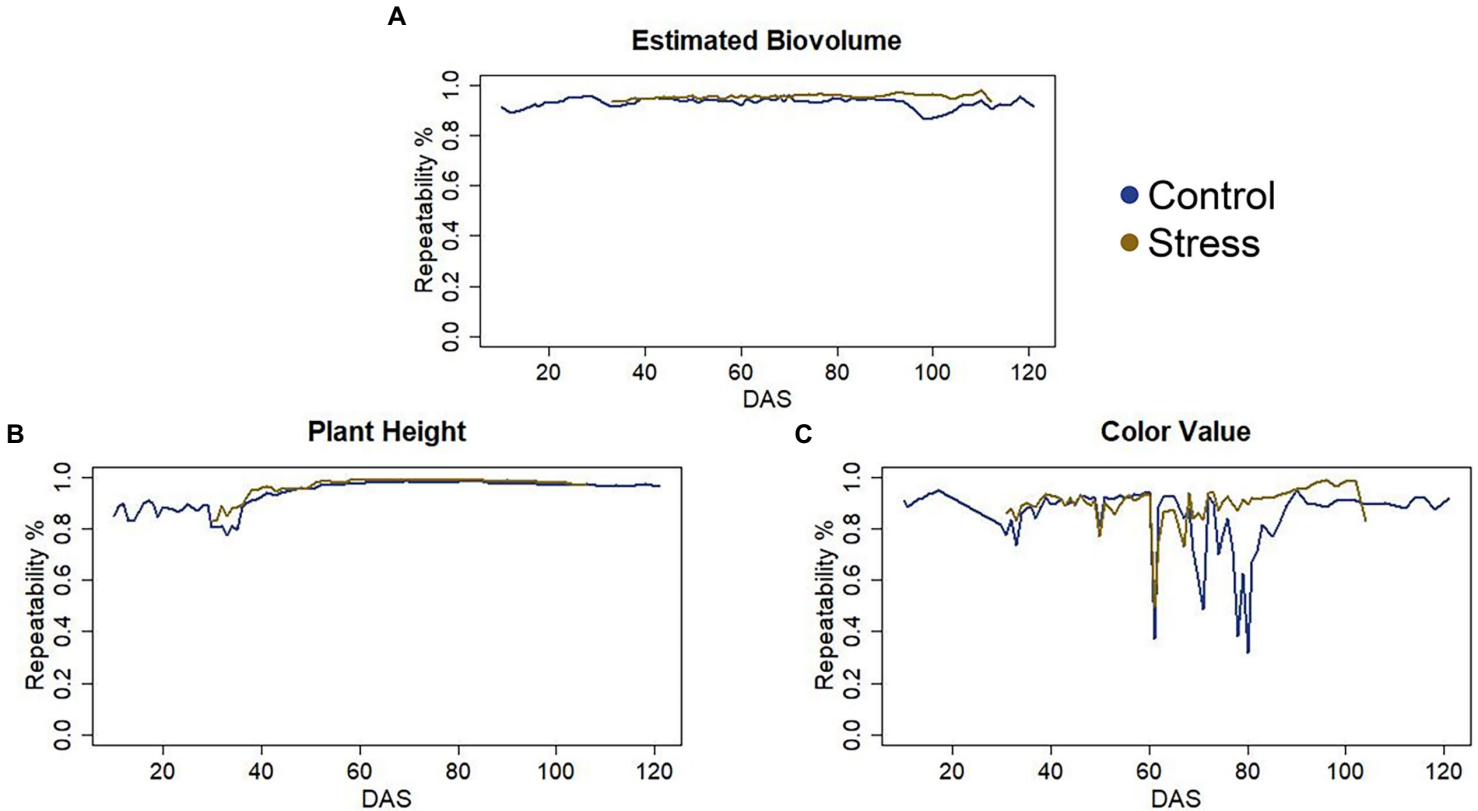
**(A)** Repeatability of the Estimated Biovolume. **(B)** Repeatability of the Plant Height. **(C)** Repeatability of the Color Value.

Data quality, represented by repeatability, was very high throughout the life cycle for PH and EB, i.e., above 85%. Except for a few days between DAS 60 and 80, repeatability was also higher than 80% for CVa (Figure 1).

**Figure 2:**
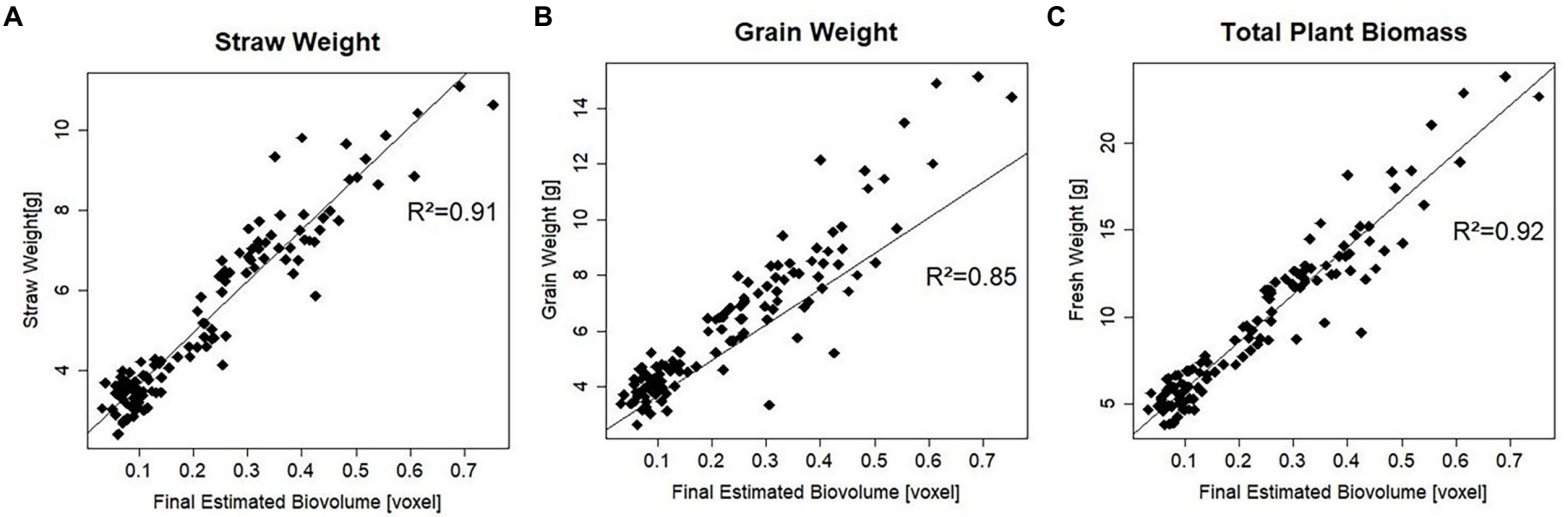
Coefficient of determination (*R*^2^) of manually measured traits at maturity with final estimated biovolume for all 120 plants. **(A)** Coefficient of determination with plant straw weight. **(B)** Coefficient of determination with plant grain weight. **(C)** Coefficient of determination with plant biomass.

To determine how well EB predicts the true value of plant biomass and its components, final EB was correlated with straw weight, grain weight, and biomass parameters measured manually at maturity (Figure 2). With a coefficient of determination of 92%, the correlation of EB with total plant biomass was the highest, closely followed by the correlation with straw weight, where the coefficient of determination was 91%. For Grain weight (GW), the correlation was slightly lower than the other two parameters, i.e.85%.

### Impact of drought stress on evaluated traits

Drought stress treatment had a strong effect on growth in all wheat lines. Regarding EB and PH significant differences between the control and the stress treatment were observed after one week of drought, from DAS 38 to harvest (Supplementary material 2). The reduction of the traits varied for selected days during mild stress (DAS 33, 40, 47 and 54) and severe stress (DAS 70, 80 and 90) from 4 to70% (Supplementary material 3). The CVa differed between treatments only during the maturation period from DAS 67 to harvest, indicating a faster maturation under drought stress (Supplementary material 2). At maturity, drought-induced losses were most severe, amounting to 47–55% decreases in biomass-related traits such as total biomass, grain weight and straw weight (Supplementary material 4).

The mean time to reach BBCH55 under stress conditions was DAS 48, 2 days earlier compared to control (Supplementary materials 4, 5). BarNir, NIL-U-2B-3, and Uzan showed a slightly but significantly earlier heading under stress conditions than under control conditions. However, significant differences between the NILs and the parental lines within the stress treatment were only present for BarNir and NIL-B-7A-2 which headed significantly 1 day later than BarNir.

The effect of drought stress was also evident for the trait “time to maturity.” The stressed plants matured on average 18 days earlier than control plants with larger variation in stress treatment compared to control (Supplementary material 6).

### QTL effects on traits measured before and at maturity

#### Effect of QTL on chromosome 7A

For the traits recorded before maturity, significant differences were found between the parents and the corresponding NILs in a few traits (Table 3). Against the background of BarNir, the QTL on chromosome 7A in NIL-B-7A-2 proved to be superior in terms of flag leaf size both under control conditions with an increase of 18% and under drought conditions with 12%. Moreover, the 7A QTL in BarNir background improved the efficiency of photosystem II in both treatments under high light conditions (Quantum Yield under High light QY-H) on both time points under severe drought (DAS 69 and 74), while the QY in the transition to low light conditions (Quantum Yield under Low light QY-L), was only higher in the NIL on DAS 74 in the well-watered treatment. The QY-LH ratio, which describes the plasticity of photosystem II, was lower on both days, indicating a lower stress effect in the NIL. However, NIL-Z-7A-5 containing the same QTL in the background of Zahir, showed no difference compared to Zahir for all these traits. In both backgrounds, no influence of the 7A-QTL on the osmotic potential was detected.

**Table 3:** Averages and standard deviation and significant differences (*t*-test) between parents and corresponding NIL of traits measured before maturity.

	BarNir	NIL-B-7A-2	*p*-value	Zahir	NIL-Z-7A-5	*p*-value	Uzan	NIL-U-2B-3	*p*-value
**Control**									
TN DAS28	3.00 ± 0.67	2.90 ± 1.10	0.81	2.30 ± 0.48	2.10 ± 0.57	0.41	2.90 ± 0.32	2.90 ± 0.74	1.00
TN DAS53	6.30 ± 1.25	5.90 ± 1.60	0.54	3.40 ± 0.52	3.00 ± 0.47	0.09	3.70 ± 1.06	4.50 ± 1.27	0.14
TN DAS70	6.60 ± 1.07	7.10 ± 1.60	0.42	3.80 ± 0.79	3.80 ± 0.63	1.00	5.70 ± 0.95	7.40 ± 2.32	**0.05**
Gain of TN DAS28 and 53	3.30 ± 1.06	3.00 ± 1.05	0.53	1.10 ± 0.57	0.90 ± 0.57	0.44	0.80 ± 1.14	1.60 ± 1.26	0.15
Gain of TN DAS53 and 70	0.30 ± 0.48	1.20 ± 0.79	**0.01**	0.40 ± 0.70	0.80 ± 0.63	0.20	2.00 ± 1.49	2.90 ± 2.42	0.33
Gain of TN DAS28 and 70	3.60 ± 0.97	4.20 ± 1.03	0.20	1.50 ± 0.85	1.70 ± 0.67	0.57	2.80 ± 0.92	4.50 ± 1.84	**0.02**
BBCH55 in DAS	46.60 ± 0.97	47.00 ± 1.49	0.49	55.60 ± 2.99	54.00 ± 2.87	0.24	50.00 ± 0.67	49.80 ± 1.40	0.69
Flag Leaf Width (mm)	14.50 ± 0.53	15.80 ± 1.75	**0.04**	18.20 ± 1.14	18.00 ± 0.82	0.66	18.00 ± 1.15	18.10 ± 1.66	0.88
Flag Leaf Length (cm)	32.28 ± 1.40	33.89 ± 3.40	0.18	24.76 ± 2.53	25.60 ± 2.17	0.44	26.45 ± 1.94	25.40 ± 1.39	0.18
Flag Leaf Area (cm^2^)	156.53 ± 6.12	188.03 ± 26.44	**0.00**	244.13 ± 20.29	242.93 ± 14.08	0.88	239.93 ± 21.03	241.88 ± 31.82	0.87
Osmotic Potential (MPa)	−1.53 ± 0.08	−1.52 ± 0.09	0.76	−1.34 ± 0.13	−1.38 ± 0.13	0.45	−1.54 ± 0.14	−1.58 ± 0.13	0.57
QY-H DAS 69	0.44 ± 0.01	0.47 ± 0.01	**0.00**	0.46 ± 0.01	0.47 ± 0.01	0.50	0.47 ± 0.01	0.47 ± 0.01	0.86
QY-H DAS 74	0.44 ± 0.01	0.46 ± 0.02	0.09	0.46 ± 0.01	0.46 ± 0.01	0.74	0.46 ± 0.01	0.46 ± 0.01	1.00
QY-L DAS 69	0.51 ± 0.01	0.51 ± 0.01	0.57	0.51 ± 0.01	0.51 ± 0.01	0.18	0.53 ± 0.02	0.53 ± 0.01	0.66
QY-L DAS 74	0.52 ± 0.01	0.53 ± 0.02	**0.04**	0.51 ± 0.02	0.51 ± 0.01	0.88	0.52 ± 0.02	0.53 ± 0.01	0.16
QY-LH Ratio DAS 69	1.15 ± 0.03	1.09 ± 0.02	**0.00**	1.09 ± 0.04	1.10 ± 0.03	0.70	1.12 ± 0.04	1.12 ± 0.02	0.75
QY-LH Ratio DAS 74	1.17 ± 0.03	1.16 ± 0.03	0.98	1.12 ± 0.04	1.12 ± 0.04	0.90	1.13 ± 0.04	1.15 ± 0.03	0.18
**Stress**									
TN DAS28	2.60 ± 0.52	2.80 ± 0.42	0.36	2.20 ± 0.79	2.00 ± 0.67	0.55	2.40 ± 0.52	3.30 ± 0.48	**0.00**
TN DAS53	4.10 ± 0.99	4.40 ± 1.07	0.53	2.70 ± 0.48	2.80 ± 0.63	0.70	3.00 ± 0.47	3.80 ± 0.42	**0.00**
TN DAS70	4.40 ± 0.97	4.60 ± 1.26	0.70	3.00 ± 0.67	3.00 ± 0.47	1.00	3.30 ± 0.48	5.10 ± 0.88	**0.00**
Gain of TN DAS28 and 53	1.50 ± 0.97	1.60 ± 0.97	0.82	0.50 ± 0.53	0.80 ± 0.92	0.38	0.60 ± 0.70	0.50 ± 0.53	0.72
Gain of TN DAS53 and 70	0.30 ± 0.48	0.20 ± 1.03	0.78	0.30 ± 0.67	0.20 ± 0.42	0.70	0.30 ± 0.82	1.30 ± 1.06	**0.03**
Gain of TN DAS28 and 70	1.80 ± 0.92	1.80 ± 1.32	1.00	0.80 ± 0.79	1.00 ± 082	0.58	0.90 ± 0.74	1.80 ± 1.03	**0.04**
BBCH55 in DAS	45.20 ± 0.63	46.20 ± 0.92	**0.01**	52.60 ± 1.84	52.80 ± 2.39	0.84	48.30 ± 0.67	47.50 ± 1.27	0.10
Flag Leaf Width (mm)	15.00 ± 1.15	15.70 ± 1.16	0.19	16.50 ± 1.72	15.90 ± 1.37	0.40	16.60 ± 0.97	16.40 ± 2.07	0.78
Flag Leaf Length (cm)	31.46 ± 1.05	35.78 ± 1.81	**0.00**	16.28 ± 3.43	17.55 ± 5.30	0.53	23.28 ± 0.67	22.96 ± 3.21	0.76
Flag Leaf Area (cm^2^)	168.53 ± 15.57	189.98 ± 26.62	**0.04**	204.60 ± 22.68	189.83 ± 25.46	0.19	213.08 ± 22.28	182.66 ± 72.86	0.22
Osmotic Potential (MPa)	−1.86 ± 0.14	−1.89 ± 0.11	0.52	−1.73 ± 0.22	−1.72 ± 0.18	0.93	−1.82 ± 0.07	−1.89 ± 0.12	0.15
QY-H DAS 69	0.42 ± 0.02	0.46 ± 0.01	**0.00**	0.45 ± 0.02	0.44 ± 0.02	0.57	0.44 ± 0.02	0.43 ± 0.02	0.11
QY-H DAS 74	0.41 ± 0.01	0.45 ± 0.01	**0.00**	0.44 ± 0.01	0.44 ± 0.01	0.82	0.42 ± 0.02	0.39 ± 0.07	0.14
QY-L DAS 69	0.49 ± 0.02	0.51 ± 0.02	0.16	0.50 ± 0.02	0.50 ± 0.01	0.45	0.51 ± 0.02	0.49 ± 0.01	0.18
QY-L DAS 74	0.49 ± 0.03	0.51 ± 0.02	0.18	0.50 ± 0.02	0.50 ± 0.02	0.85	0.48 ± 0.03	0.47 ± 0.01	0.17
QY-LH Ratio DAS 69	1.18 ± 0.03	1.12 ± 0.04	**0.00**	1.11 ± 0.03	1.14 ± 0.05	0.20	1.14 ± 0.04	1.16 ± 0.05	0.43
QY-LH Ratio DAS 74	1.20 ± 0.06	1.14 ± 0.05	**0.02**	1.14 ± 0.05	1.15 ± 0.04	0.72	1.15 ± 0.04	1.29 ± 0.43	0.31

The mean followed by the standard deviation is shown. TN, tiller number; QY-H, quantum yield of photosystem II under high-light and QY-L under low light; QY-LH, ratio of QY-L to QY-H; DAS, days after sowing, for both = for control and stress treatment. A *t*-test with a significance level of *p* < 0.05 was used to detect significant differences between lines and has been highlighted here in bold.

The treatment has a significant effect on the time of heading (BBCH55; Table 3, Supplementary material 5). For the control treatment, the median of all six lines is at DAS 48 and for the stress treatment at DAS 50. However, this effect also depended on the genetic background and the treatment. NIL-B-7A-2 showed a significantly later heading of 1 day under stress conditions (Table 3). In contrast, in the NIL-Z-7A-5, also carrying the QTL 7A but in the Zahir background, a significantly earlier heading was observed in the control treatment. In the line NIL-U-2B-3 a significantly earlier heading under stress was detected compared to Uzan. For the osmotic potential, no significant differences were found for any of the lines (Table 3).

The 7A-QTL segment introduced in BarNir, NIL-B-7A-2, conferred significantly 25% higher plant biomass in the control and 33% higher plant biomass in the drought stress treatment at maturity (Table 4). In addition to increased PH, the 7A QTL in BarNir background increased also awn length, peduncle length and the length of the last internode in both treatments, while the ear length was decreased in well-watered conditions. In the Zahir background, no effect of the 7A QTL on height, awn length and ear length was detected, while the peduncle was longer only in well-watered conditions but the last internode was longer only in drought conditions in the respective NIL. In the BarNir background, the QTL caused increased grain and straw weight in both treatments, while HI was unaffected. These traits were not affected in the Zahir background (Table 4). Moreover, in the BarNir background, the 7A QTL significantly improved WUE, TKW and seed size parameters in both treatments. In the Zahir background improved WUE and TKW was observed only under drought stress, the higher TKW resulted from a higher seed area, or seed length.

**Table 4:** Averages and standard deviation and significant differences (*t*-test) between parents and corresponding NIL of traits manually measured at maturity.

	BarNir	NIL-B-7A-2	*p*-value	Zahir	NIL-Z-7A-5	*p*-value	Uzan	NIL-U-2B-3	*p*-value
**Control**									
Plant height (cm)	47.68 ± 1.58	72.61 ± 2.85	**0.00**	72.74 ± 3.27	74.78 ± 3.12	0.17	62.48 ± 1.86	54.74 ± 3.42	**0.00**
Main Ear Awn Length (cm)	6.26 ± 0.59	8.35 ± 0.70	**0.00**	6.35 ± 0.52	6.71 ± 0.23	0.07	11.71 ± 0.91	11.15 ± 1.07	0.22
Ear Length (cm)	10.23 ± 0.45	8.60 ± 3.06	0.11	10.17 ± 0.59	9.88 ± 0.47	0.24	5.20 ± 1.08	5.01 ± 0.30	0.60
Culm Length (cm)	37.45 ± 1.49	64.24 ± 4.39	**0.00**	62.37 ± 2.75	64.90 ± 2.99	0.09	57.28 ± 1.92	49.73 ± 3.15	**0.00**
Peduncle length (cm)	6.70 ± 1.26	13.16 ± 5.28	**0.00**	10.47 ± 1.91	13.20 ± 2.14	**0.01**	19.18 ± 12.23	11.64 ± 2.30	0.07
Last Internode length (cm)	16.09 ± 0.60	18.54 ± 7.00	0.28	17.25 ± 0.72	17.83 ± 0.59	0.07	11.62 ± 12.24	14.35 ± 0.77	0.49
Number of Spikes	6.80 ± 1.14	6.90 ± 1.73	0.88	4.00 ± 1.05	3.90 ± 0.57	0.79	5.30 ± 0.67	6.30 ± 1.25	**0.04**
Number of fertile Spikes	6.50 ± 1.08	6.90 ± 1.73	0.54	4.00 ± 1.05	3.90 ± 0.57	0.79	4.90 ± 0.99	5.80 ± 1.40	0.11
Plant Biomass (g)	1,251 ± 2.02	16.94 ± 5.89	**0.04**	13.27 ± 4.00	11.76 ± 1.85	0.29	11.69 ± 1.83	12.51 ± 3.22	0.49
Plant Grain Weight (g)	7.60 ± 1.24	10.50 ± 3.95	**0.04**	8.70 ± 2.34	7.50 ± 1.08	0.15	6.90 ± 1.70	7.40 ± 1.99	0.54
Plant Straw Weight (g)	6.35 ± 1.02	8.42 ± 2.24	**0.02**	7.61 ± 1.80	6.97 ± 1.00	0.33	6.86 ± 0.59	6.97 ± 1.56	0.84
Plant Harvest Index	0.51 ± 0.04	0.51 ± 0.05	0.88	0.46 ± 0.04	0.45 ± 0.05	0.48	0.44 ± 0.07	0.46 ± 0.05	0.36
Biomass WUE (g/l)	1.53 ± 0.09	1.66 ± 0.26	0.14	1.46 ± 0.20	1.43 ± 0.14	0.73	1.27 ± 0.13	1.33 ± 0.19	0.42
Plant TKW (g)	37.30 ± 2.16	44.60 ± 6.55	**0.00**	47.43 ± 4.75	47.55 ± 2.18	0.95	50.05 ± 570	48.32 ± 3.05	0.41
Plant Seed area (mm^2^)	13.92 ± 0.54	15.94 ± 1.61	**0.00**	16.29 ± 0.93	16.69 ± 0.49	0.24	18.66 ± 0.97	17.47 ± 0.71	**0.01**
Plant Seed width (mm)	3.25 ± 0.10	3.48 ± 0.20	**0.00**	3.73 ± 0.13	3.68 ± 0.05	0.31	3.82 ± 0.13	3.76 ± 0.11	0.31
Plant Seed Length (mm)	5.94 ± 0.10	6.32 ± 0.25	**0.00**	6.07 ± 0.23	6.29 ± 0.14	**0.02**	6.92 ± 0.16	6.54 ± 0.11	**0.00**
Plant Grain Number	204.90 ± 34.01	227.80 ± 65.98	0.34	182.50 ± 45.45	156.30 ± 23.19	0.12	136.10 ± 27.32	152.00 ± 37.74	0.29
Grains per Ear	31.80 ± 4.47	32.98 ± 5.10	0.59	46.47 ± 8.29	40.34 ± 4.92	0.06	28.35 ± 6.30	26.55 ± 4.24	0.46
Main Ear Spikelet Number	18.10 ± 0.99	18.80 ± 1.03	0.14	23.80 ± 1.62	22.60 ± 1.35	0.09	14.80 ± 0.42	14.20 ± 0.79	**0.05**
Main Ear Grain Number	32.20 ± 6.71	34.80 ± 4.16	0.31	52.80 ± 6.66	45.40 ± 4.74	**0.01**	32.90 ± 2.13	31.00 ± 3.53	0.16
Main Ear Grains per Spikelet	56.92 ± 11.80	67.16 ± 5.82	**0.02**	119.24 ± 13.26	104.21 ± 13.16	**0.02**	89.21 ± 5.99	84.64 ± 9.84	0.23
Main Ear Biomass (g)	1.48 ± 0.17	2.01 ± 0.29	**0.00**	3.08 ± 0.52	2.73 ± 0.29	0.08	2.09 ± 0.28	1.90 ± 0.24	0.11
Main Ear Grain Weight (g)	1.21 ± 0.16	1.65 ± 0.26	**0.00**	2.55 ± 0.43	2.26 ± 0.25	0.08	1.72 ± 0.28	1.58 ± 0.18	0.19
Main Ear Straw (g)	0.27 ± 0.06	0.36 ± 0.08	**0.01**	0.54 ± 0.10	0.48 ± 0.05	0.10	0.37 ± 0.04	0.32 ± 0.07	0.06
Main Ear Harvest Index	0.82 ± 0.04	0.82 ± 0.04	0.89	0.83 ± 0.01	0.83 ± 0.01	0.94	0.82 ± 0.04	0.83 ± 0.02	0.36
Main Ear TKW (g)	38.16 ± 4.60	47.26 ± 4.61	**0.00**	48.04 ± 3.60	49.87 ± 4.44	0.32	52.29 ± 7.97	50.92 ± 2.23	0.61
Main Ear Seed Area (mm^2^)	14.38 ± 0.95	16.53 ± 1.18	**0.00**	16.17 ± 0.92	16.72 ± 0.95	0.21	18.95 ± 1.16	17.71 ± 0.55	**0.01**
Watersum (l)	8.14 ± 0.99	9.91 ± 2.25	**0.04**	8.96 ± 1.68	8.20 ± 0.71	0.21	9.21 ± 1.10	9.28 ± 1.62	0.91
**Stress**									
Plant height (cm)	44.82 ± 0.99	67.21 ± 2.54	**0.00**	68.72 ± 1.79	67.83 ± 2.47	0.39	58.08 ± 1.29	48.03 ± 2.25	**0.00**
Main Ear Awn Length (cm)	6.16 ± 0.59	8.06 ± 0.72	**0.00**	5.67 ± 0.48	5.62 ± 0.56	0.83	11.46 ± 1.09	10.65 ± 0.88	0.09
Ear Length (cm)	10.57 ± 0.42	8.18 ± 3.88	0.07	8.76 ± 3.20	9.23 ± 3.28	0.75	4.84 ± 1.71	4.63 ± 1.66	0.78
Culm Length (cm)	34.25 ± 0.8	59.03 ± 4.09	**0.00**	59.82 ± 3.30	58.47 ± 3.57	0.39	52.10 ± 2.26	43.70 ± 3.25	**0.00**
Peduncle length (cm)	4.98 ± 0.97	14.86 ± 3.90	**0.00**	10.42 ± 3.84	6.10 ± 20.73	0.53	14.45 ± 5.12	9.83 ± 4.10	**0.04**
Last Internode length (cm)	15.37 ± 0.37	19.21 ± 0.94	**0.00**	12.65 ± 21.33	15.70 ± 0.54	0.66	10.01 ± 11.14	11.45 ± 4.08	0.71
Number of Spikes	3.60 ± 0.84	4.10 ± 0.88	0.21	2.50 ± 0.53	2.40 ± 0.52	0.67	3.00 ± 0.00	3.80 ± 0.42	**0.00**
Number of fertile Spikes	3.33 ± 0.50	4.10 ± 0.88	**0.03**	2.40 ± 0.52	2.40 ± 0.52	1.00	3.00 ± 0.00	3.80 ± 0.42	**0.00**
Plant Biomass (g)	5.42 ± 0.55	8.09 ± 1.39	**0.00**	4.97 ± 0.76	5.12 ± 0.86	0.69	5.66 ± 0.75	6.43 ± 1.16	0.10
Plant Grain Weight (g)	4.00 ± 0.34	5.70 ± 0.91	**0.00**	4.00 ± 0.62	4.00 ± 0.63	0.88	3.90 ± 0.55	4.40 ± 0.78	0.18
Plant Straw Weight (g)	3.02 ± 0.31	4.48 ± 0.60	**0.00**	3.34 ± 0.32	3.39 ± 0.30	0.69	3.63 ± 0.35	3.69 ± 0.53	0.78
Plant Harvest Index	0.50 ± 0.03	0.49 ± 0.04	0.37	0.41 ± 0.06	0.41 ± 0.05	0.88	0.42 ± 0.03	0.46 ± 0.03	**0.01**
Biomass WUE (g/l)	1.93 ± 0.20	2.17 ± 0.25	**0.03**	1.70 ± 0.21	1.79 ± 0.19	0.34	1.70 ± 0.11	1.91 ± 0.19	**0.01**
Plant TKW (g)	33.05 ± 3.33	39.29 ± 2.11	**0.00**	38.17 ± 6.32	44.01 ± 5.27	**0.04**	46.85 ± 4.21	46.71 ± 3.48	0.94
Plant Seed area (mm^2^)	13.17 ± 0.67	14.71 ± 0.73	**0.00**	13.88 ± 1.47	15.42 ± 1.20	**0.02**	17.48 ± 0.74	16.75 ± 0.53	**0.02**
Plant Seed width (mm)	3.08 ± 0.11	3.30 ± 0.08	**0.00**	3.39 ± 0.20	3.52 ± 0.13	0.09	3.69 ± 0.13	3.64 ± 0.09	0.24
Plant Seed Length (mm)	5.94 ± 0.10	6.18 ± 0.20	**0.00**	5.67 ± 0.34	6.06 ± 0.26	**0.01**	6.68 ± 0.11	6.49 ± 0.10	**0.00**
Plant Grain Number	120.30 ± 16.23	140.60 ± 26.67	**0.05**	105.90 ± 17.38	90.80 ± 21.29	0.10	81.20 ± 9.66	92.60 ± 17.30	0.09
Grains per Ear	35.01 ± 4.80	34.57 ± 2.84	0.81	44.73 ± 4.89	38.00 ± 5.73	**0.01**	28.17 ± 3.17	24.25 ± 3.11	**0.01**
Main Ear Spikelet Number	18.70 ± 0.48	18.70 ± 1.06	1.00	22.40 ± 1.17	22.20 ± 1.62	0.76	14.80 ± 0.92	13.60 ± 1.17	**0.02**
Main Ear Grain Number	37.60 ± 5.68	39.70 ± 6.91	0.47	51.40 ± 3.92	41.10 ± 7.78	**0.00**	30.20 ± 2.62	27.90 ± 2.33	**0.05**
Main Ear Grains per Spikelet	67.13 ± 10.19	73.73 ± 11.94	0.20	121.98 ± 13.69	90.19 ± 17.93	**0.00**	80.11 ± 6.65	74.25 ± 8.03	0.10
Main Ear Biomass (g)	1.62 ± 0.09	2.06 ± 0.22	**0.00**	2.37 ± 0.25	2.23 ± 0.17	0.18	1.91 ± 0.23	1.62 ± 0.17	**0.00**
Main Ear Grain Weight (g)	1.31 ± 0.10	1.71 ± 0.19	**0.00**	1.95 ± 0.23	1.82 ± 0.16	0.17	1.56 ± 0.21	1.36 ± 0.13	**0.02**
Main Ear Straw (g)	0.31 ± 0.03	0.35 ± 0.06	**0.04**	0.42 ± 0.06	0.41 ± 0.06	0.74	0.35 ± 0.04	0.26 ± 0.04	**0.00**
Main Ear Harvest Index	0.81 ± 0.02	0.83 ± 0.02	0.08	0.82 ± 0.02	0.82 ± 0.02	0.55	0.81 ± 0.02	0.84 ± 0.02	**0.00**
Main Ear TKW (g)	35.52 ± 4.66	43.82 ± 5.61	**0.00**	37.97 ± 4.37	45.29 ± 6.10	**0.01**	51.40 ± 3.74	48.76 ± 2.58	0.08
Main Ear Seed Area (mm^2^)	14.44 ± 0.77	16.25 ± 1.27	**0.00**	13.93 ± 1.05	15.68 ± 1.48	**0.01**	18.35 ± 0.69	17.24 ± 0.50	**0.00**
Watersum (l)	2.82 ± 0.14	3.73 ± 0.43	**0.00**	2.92 ± 0.17	2.85 ± 0.21	0.43	3.31 ± 0.30	3.34 ± 0.38	0.86

A t-test with a significance level of *p* < 0.05 was used to detect significant differences between lines and has been highlighted here in bold.

#### Effect of QTL on chromosome 2B

The wild emmer wheat QTL on chromosome 2B in NIL-U-2B-3 caused a more pronounced tiller number of 35% more tillers under stress compared to the recurrent parent Uzan (Table 3). Furthermore, a small effect on heading was found, the NIL-U-2B-3 headed about 1 day earlier under drought stress compared to Uzan. No effect of the 2B QTL was detected for the osmotic potential. At maturity, the NIL-U-2B-3 plants were smaller in both treatments (Table 4). In accordance with the higher tillering during growth, a higher number of fertile spikes was also observed under drought stress, along with an improved WUE. Besides, a higher HI in both treatments was obtained. However, the higher number of tillers was linked with a lower TKW and its components and a lower grain number per ear.

### Comparison of HTP experiment with the field/screenhouse experiment

The QTL effects for common traits between the HTP study and the field/screenhouse experiments from Merchuk-Ovnat et al. (2016a) were compared by calculating the relative percentage difference of the respective NIL and the corresponding parent in the HTP and each of the two field/screenhouse experiments and by looking at the phenotypic mean values in HTP and the two field/screenhouse experiments (Supplementary material 7; Table 5). Mostly, the advantageous effect of the QTL was visible in HTP and in at least one of the field/screenhouse experiments. However, the magnitude of the effects differed, sometimes the effect was more pronounced in the HTP experiment, in some cases more in the field/screenhouse. Most consistent were the results of both studies for the QTL effect in NIL-B-7A-2 compared to BarNir, while for the 7A QTL in the background of Zahir, more significant differences for yield-related traits were found in the field/screenhouse that were not detected in the HTP experiment. Notably, also the QTL effects between the 2 years of field/screenhouse experiments varied. Considering the absolute values, the BBCH55 stage occurred 10–15 days earlier in the controlled environment of the HTP experiment. Compared to the field, plants in the HTP experiment were smaller and had a lower grain yield and lower values for other grain-yield related parameters across all genotypes and treatments such as TKW or HI (Table 5).

**Table 5:** Comparison of the absolute values (mean) of the NILs and the parents of common traits of the HTP experiment and field/screenhouse experiments.

Trait		Treament	HTP			FY1			FY2		
		**BarNir**	**NIL-B-7A-2**	**BarNir**	**NIL-B-7A-2**	**BarNir**	**NIL-B-7A-2**
Culm Length (cm)	Control	37	64	***	60	75	***	59	70	***
	Stress	34	59	***	56	70	***	50	62	***
BBCH55	Control	47	47		64	66	***	63	67	***
	Stress	45	46	**	63	65	***	62	66	***
Grains per Ear	Control	32	33		57	55		54	48	
	Stress	35	35		59	63		44	46	
Grain Yield (g)	Control	7.60	10.50		24	28.00		18.00	16.10	
	Stress	4.00	570	***	11.80	18.80	*	7.30	11.80	*
Harvest Index	Control	0.51	0.51		0.57	0.56		0.52	0.56	
	Stress	0.50	0.49		0.52	0.56		0.55	0.53	
Osmotic Potential (Mpa)	Control	−1.53	−1.52		−1.32	−1.26		−1.19	−1.26	
	Stress	−1.86	−1.89		−1.76	−1.61		−1.33	−1.52	***
Spikes per Plant	Control	7	7		13	14		10	8	
	Stress	4	4		7	9		5	7	
TKW	Control	37	45	***	49	55	**	48	48	
	Stress	33	39	***	41	47	**	48	50	
Plant Biomass (g)	Control	12.51	16.94	*	42.20	50.90		34.70	33.30	
	Stress	542	8.09	***	22.50	34.10	*	13.20	22.10	*
		**Zahir**	**NIL-Z-7A-5**		**Zahir**	**NIL-Z-7A-5**		**Zahir**	**NIL-Z-7A-5**	
Culm Length (cm)	Control	62	65		74	83	**	68	76	**
	Stress	60	58		69	75	*	63	67	*
BBCH55	Control	56	54		64	71	***	61	67	***
	Stress	53	53		62	71	***	59	67	***
Grains per Ear	Control	46	40		63	66		66	62	
	Stress	45	38		61	69		52	49	
Grain Yield (g)	Control	8.70	7.50		21.00	29.80	**	15.80	21.10	*
	Stress	4.00	4.00		12.90	17.00		8.10	11.00	
Harvest Index	Control	0.46	0.45		0.56	0.55		0.53	0.51	
	Stress	0.41	0.41		0.54	0.51		0.57	0.53	
Osmotic Potential (Mpa)	Control	−1.34	−1.38		−1.17	−1.18		−1.12	−1,15	
	Stress	−1.73	−1.72		−1.46	−1.54		−1.27	−1,22	
Spikes per Plant	Control	4	4		8	10		6	8	*
	Stress	3	2		5	7	*	4	5	*
TKW	Control	47	48		55	55		52	56	
	Stress	38	44		49	50		55	55	
Plant Biomass (g)	Control	13.27	11.76		39.80	54.60	**	29.50	41.20	*
	Stress	4.97	5.12		23.70	33.30		14.10	20.50	
		**Uzan**	**NIL-U-2B-3**		**Uzan**	**NIL-U-2B-3**		**Uzan**	**NIL-U-2B-3**	
Culm Length (cm)	Control	57	50	***				65	72	*
	Stress	52	44	***				54	60	*
BBCH55	Control	50	50					67	68	
	Stress	48	48					65	67	
Grains per Ear	Control	28	27					55	46	
	Stress	28	24	*				40	45	
Grain Yield (g)	Control	6.90	7.40					13.30	19.70	*
	Stress	3.90	4.40					4.30	11.00	*
Harvest Index	Control	0.44	0.46					0.52	0.49	
	Stress	0.42	0.46	*				0.45	0.52	
Osmotic Potential (Mpa)	Control	−154	−1.58					−1.18	−1.20	
	Stress	−1.82	−1.89					−1.40	−1.40	
Spikes per Plant	Control	5	6	*				5	8	***
	Stress	3	4	***				3	5	
TKW	Control	50	48					62	68	*
	Stress	47	47					60	66	**
Plant Biomass (g)	Control	11.69	12.51					25.60	40.00	*
	Stress	5.66	6.43					9.60	21.50	

The values for the field/screenhouse are taken from Merchuk-Ovnat et al. (2016a, Table 3, Supplementary Tables S4, S5). HTP, high-throughput phenotyping experiment; FY, field/screenhouse experiment year 1 and 2; BBCH55, heading. Significance data is from the original tables of Merchuk-Ovnat et al. (2016a) and Tables 3, 4. Mean comparisons by Student’s t-test between each line and its recurrent parent (*, **, ***) under control and drought stress treatments at p < 0.05, 0.01, 0.001, respectively.

Significant differences between parents and NILs were found for several traits across the environments. For example, NIL-B-7A-5 was significantly larger than BarNir in all environments, as measured by culm length. NIL-U-2B-3 was significantly smaller in the HTP experiment and significantly larger in the field experiment. The increased grain yield and plant biomass for NIL-B-7A-5 was also significantly greater for all environments between 43 and 69%. For TKG, the difference between the first field/screenhouse experiment and the HTP experiment was equally significant. In both environments, NIL-U-2B-3 showed a number of spikes, but it was only significant under control for both environments.

### Dynamic phenotyping revealed effect of wild emmer wheat QTLs on drought resilience

#### Effect of QTL on chromosome 7A for NIL-B-7A-2

**Figure 3:**
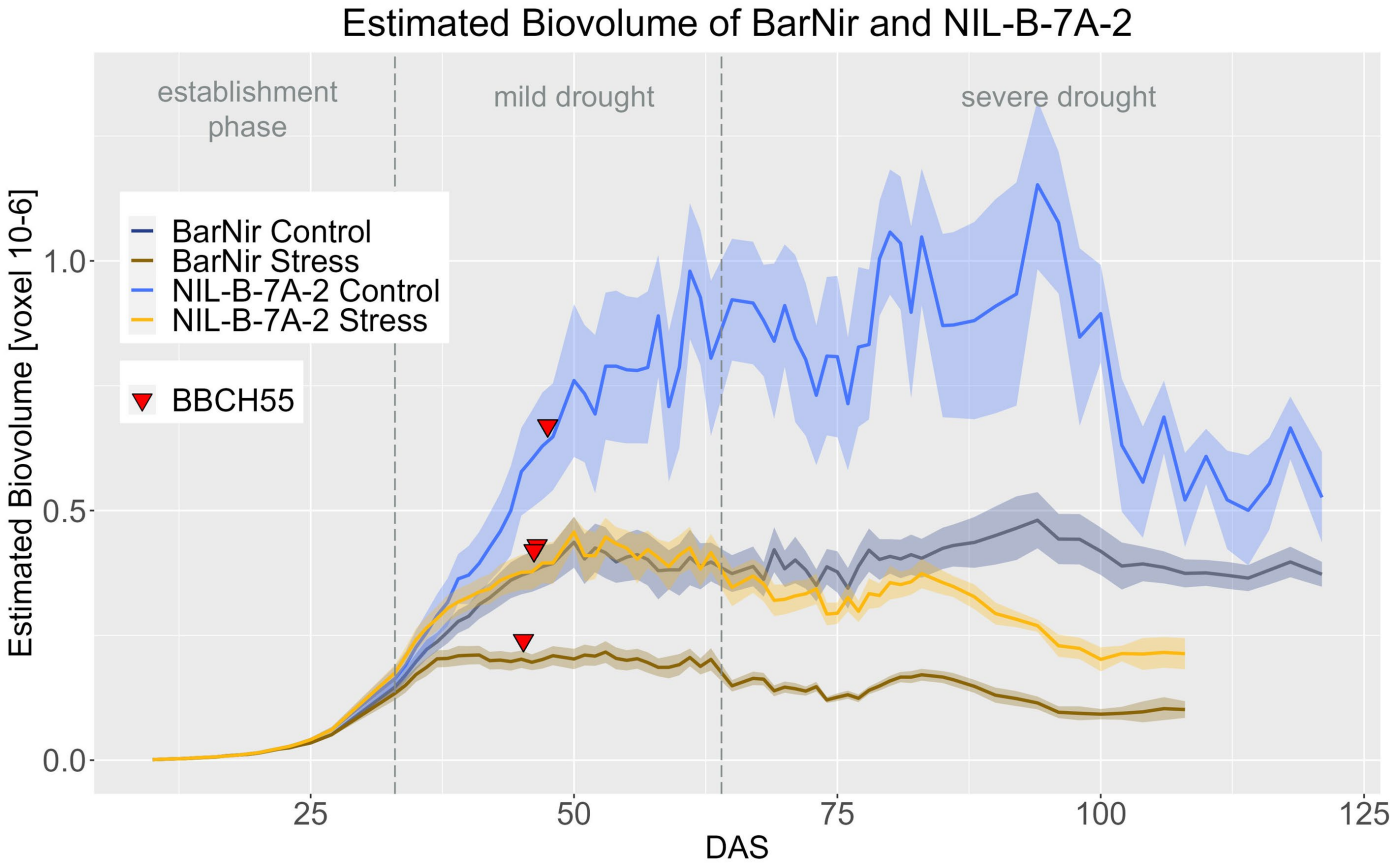
A Estimated biovolume of BarNir and NIL-B-7A-2 based on the calculated BLUEs values. DAS, days after sowing; BBCH55, heading day. The last DAS for each genotype was chosen for the DAS where the last time 60% of the plants were imaged (as mature plants were taken off subsequently). The shadows describe the confidence interval; as long as the shadows of the individual lines (parent and corresponding NIL) do not overlap, the significance level of *a* = 0.05 is not reached and therefore a significant difference exists.

Significant differences were found for EB between BarNir and NIL-B-7A-2 for the period from DAS 45–102 in the control treatment and DAS 33–102 in stress treatment (Figure 3; Supplementary materials 8, 9). Thus, differences were observed in both treatments, but they occurred earlier under drought. In both treatments, the NIL showed a higher EB. Furthermore, when testing whether the treatment had a significant effect on the EB of each genotype, NIL-B-7A-2 showed a six-day later response to drought stress treatment than BarNir (Supplementary materials 10).

**Figure 4:**
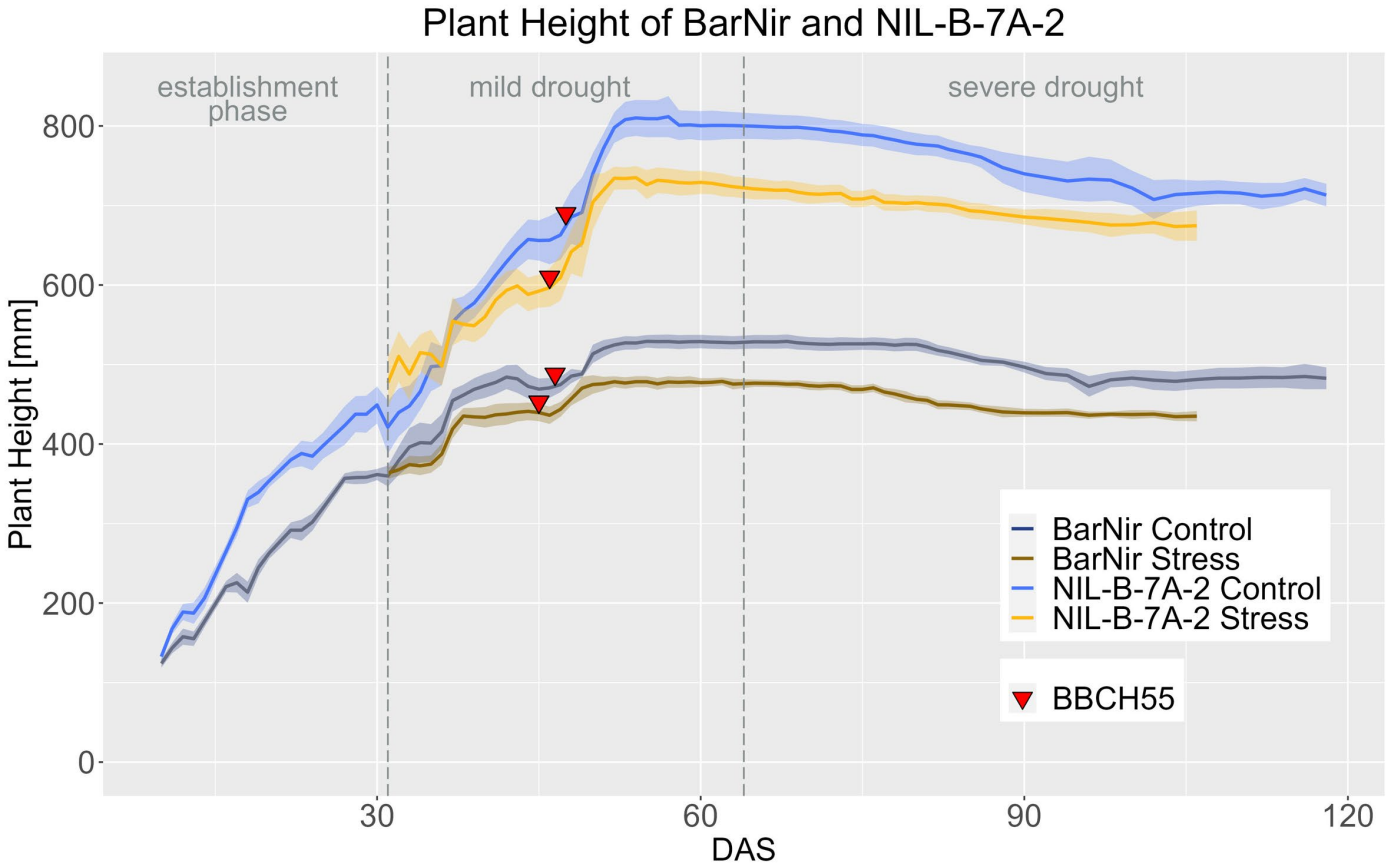
Plant height of BarNir and NIL-B-7A-2 based on the calculated BLUEs values. DAS, days after sowing; BBCH55, heading day. The last DAS for each genotype was chosen for the DAS where the last time 60% of the plants were imaged (as mature plants were taken off subsequently). The shadows describe the confidence interval; as long as the shadows of the individual lines do not overlap, the significance level of *a* = 0.05 is not reached and therefore a significant difference exists.

Consistent with the observations at maturity, plants of NIL-B-7A-2 were significantly taller than those of the parent BarNir throughout the entire life cycle under both conditions (Figure 4; Supplementary materials 11–14). Similar to EB, the PH in the NIL showed a response to drought stress 7 days later than in BarNir (Supplementary material 14).

**Figure 5:**
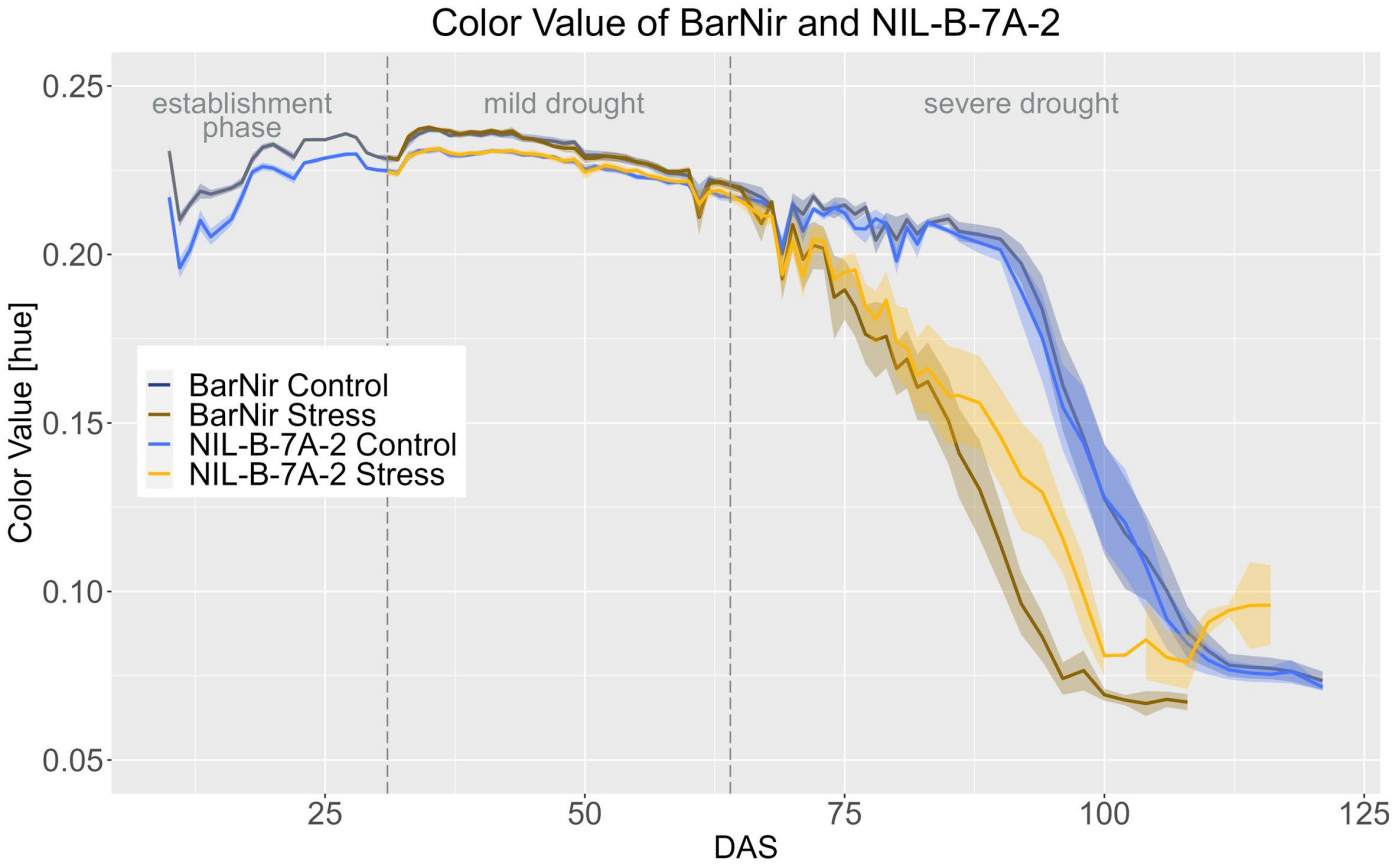
Color value of BarNir and NIL-B-7A-2 based on the calculated BLUEs values. DAS, days after sowing; BBCH55, heading day. The last DAS for each genotype was chosen for the DAS where the last time 60% of the plants were imaged (as mature plants were taken off subsequently). The shadows describe the confidence interval; as long as the shadows of the individual lines do not overlap, the significance level of *a* = 0.05 is not reached and therefore a significant difference exists.

The QTL 7A affected the CVa. Until around DAS 60, the NIL in the BarNir background had a significantly slightly lower CVa in both treatments in the BarNir background. However, in the severe drought treatment during the late ripening phase, plants of the NIL had a significantly higher CVa from DAS 90 onwards, showing a slower ripening of the NIL (Figure 5; Supplementary material 15).

The differences between the treatments were significant from DAS 70 and 71 onwards for the NIL-B-7A-2 and BarNir, respectively (Supplementary materials 16–18).

#### Effect of QTL on chromosome 7A for NIL-Z-7A-5

**Figure 6:**
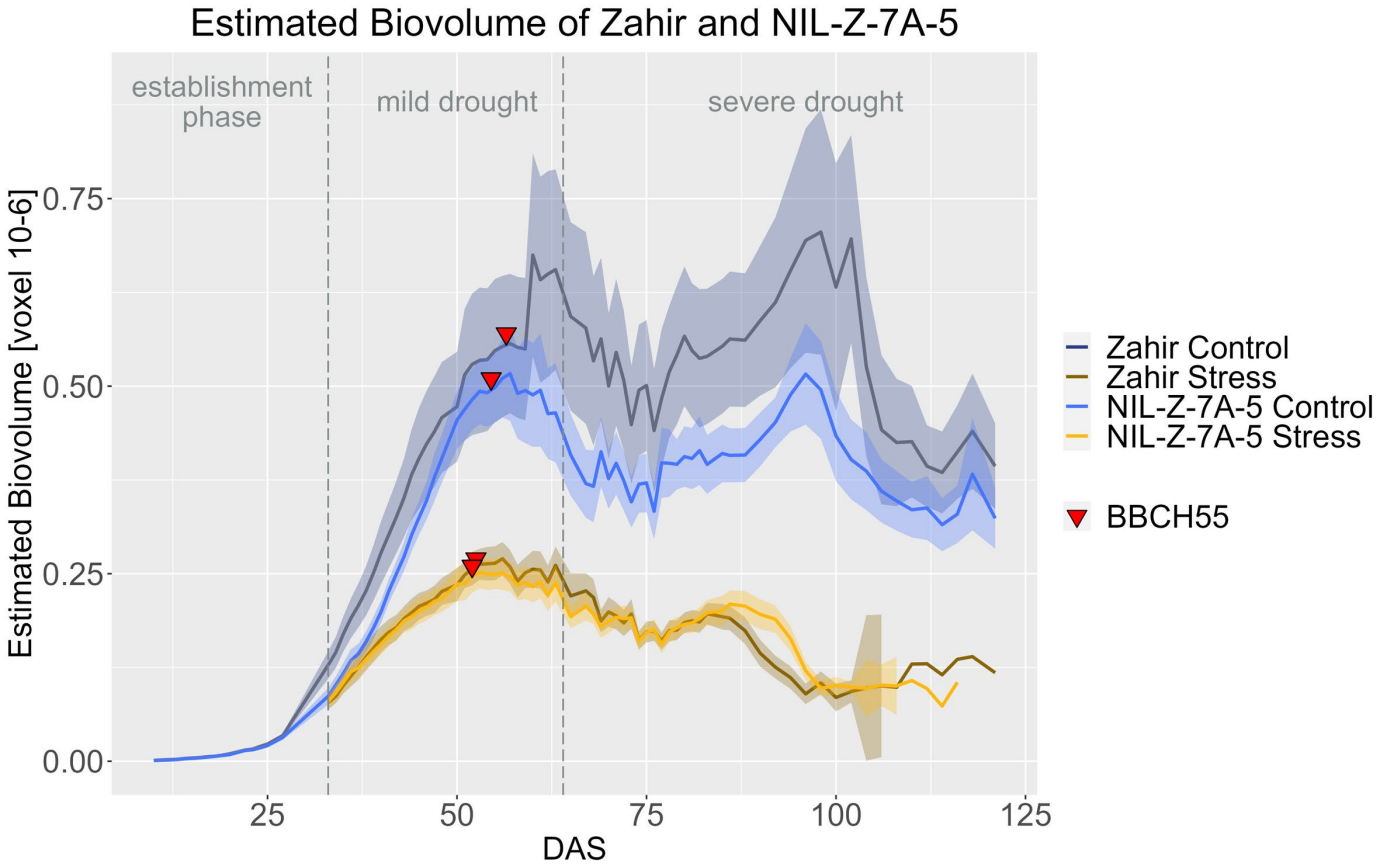
Estimated biovolume of Zahir and NIL-Z-7A-5 based on the calculated BLUEs values. DAS, days after sowing; BBCH55, heading day. The last DAS for each genotype was chosen for the DAS where the last time 60% of the plants were imaged (as mature plants were taken off subsequently). The shadows describe the confidence interval; as long as the shadows of the individual lines do not overlap, the significance level of *a* = 0.05 is not reached and therefore a significant difference exists.

NIL-Z-7A-5 produced less EB in both treatments compared to Zahir, with this effect being more pronounced in the control treatment than in the stress treatment. However, the difference between NIL and parent was significant only for a few DAS, such as DAS 10 to 13 in the plant establishment phase and during the mild stress phase from DAS 33 to 43 (Figure 6; Supplementary materials 8, 11). However, the standard deviation for EB under control was high for Zahir and NIL, which might have confounded a larger EB effect of the QTL (Supplementary material 9). Nevertheless, also in the stress treatment, the two genotypes had a very similar EB. Only in the late ripening phase, during DAS 90–96, the EB was significantly higher under drought in NIL-Z-7A-5, showing a positive effect of the 7A QTL (Supplementary material 8). Moreover, when testing if the treatment had a significant influence on EB for each genotype, NIL-Z-7A-5 showed a seven-days later reaction to the drought compared to Zahir, similar as seen in the BarNir background (Supplementary material 10).

**Figure 7:**
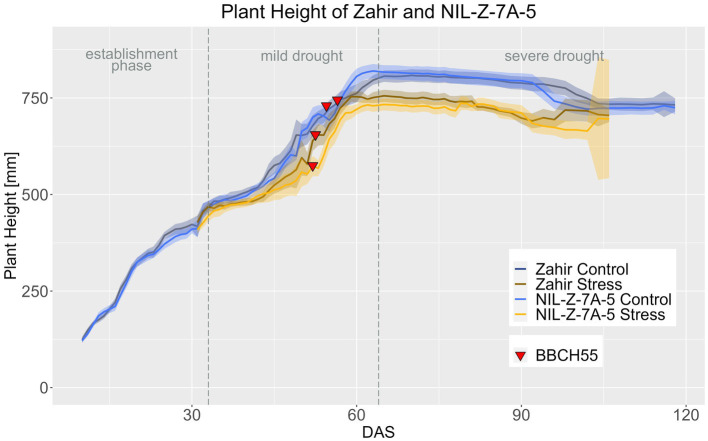
Plant height of Zahir and NIL-Z-7A-5 based on the calculated BLUEs values. DAS, days after sowing; BBCH55, heading day. The last DAS for each genotype was chosen for the DAS where the last time 60% of the plants were imaged (as mature plants were taken off subsequently). The shadows describe the confidence interval; as long as the shadows of the individual lines do not overlap, the significance level of *a* = 0.05 is not reached and therefore a significant difference exists.

In the Zahir background in both treatments, Zahir and NIL-Z-7A-5 did not differ in PH throughout the life cycle, which is in accordance with the absent difference for PH at maturity (Figure 7; Supplementary materials 11–14). The differences in PH between the treatments became significant for Zahir from DAS 62 onwards and for NIL-Z-7A-5 from DAS 57 on, so the QTL carrying NIL reduced PH 5 days earlier compared to the parental line (Supplementary material 17), which contrasts with the observed later response to drought of EB of NIL-Z-7A-5.

**Figure 8:**
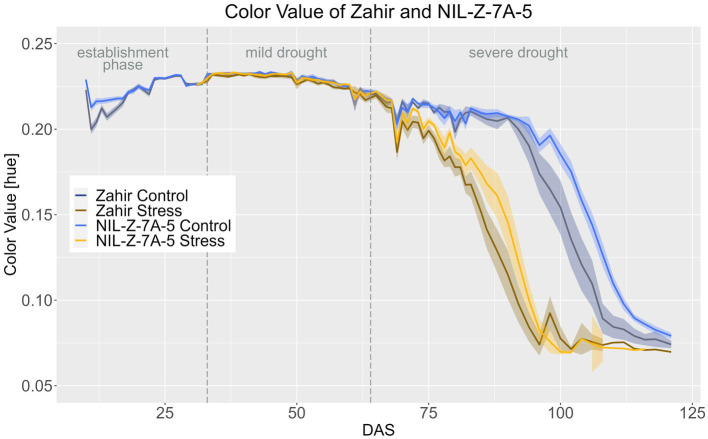
Color value of Zahir and NIL-Z-7A-5 based on the calculated BLUEs values. DAS, days after sowing; BBCH55, heading day. The last DAS for each genotype was chosen for the DAS where the last time 60% of the plants were imaged (as mature plants were taken off subsequently). The shadows describe the confidence interval; as long as the shadows of the individual lines do not overlap, the significance level of *a* = 0.05 is not reached and therefore a significant difference exists.

**Figure 9:**
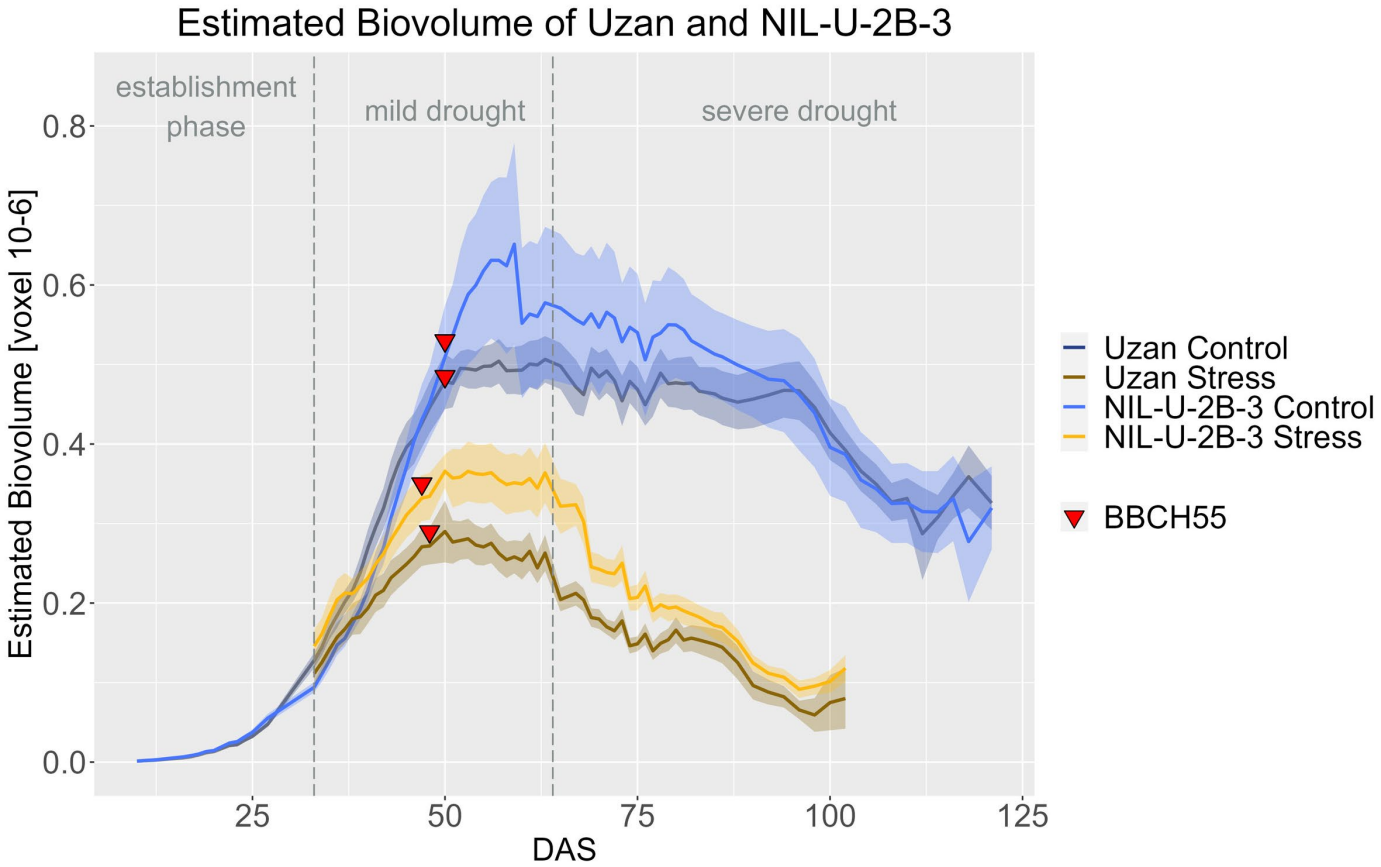
Color value of Uzan and NIL-U-2B-3 based on calculated BLUEs values.DAS, days after sowing; BBCH55, heading day. The last DAS for each genotype was chosen for the DAS where the last time 60% of the plants were imaged (as mature plants were taken off subsequently). The shadows describe the confidence interval; as long as the shadows of the individual lines do not overlap, the significance level of *a* = 0.05 is not reached and therefore a significant difference exists.

In Zahir background, the 7A QTL showed no effect on CVa until the ripening stage and the treatment effect was significant from DAS 69 onwards for Zahir and from DAS 72 for NIL-Z-7A-5 (Figure 8; Supplementary materials 16–18). However, NIL-Z-7A-5 had significantly higher CVa from DAS 98–121 in the control treatment and DAS 82 - DAS 92 in the stress treatment (Figure 9; Supplementary material 15), showing a slower ripening arising from the 7A QTL.

#### Effect of QTL on chromosome 2B for NIL-U-2B-3

**Figure 10:**
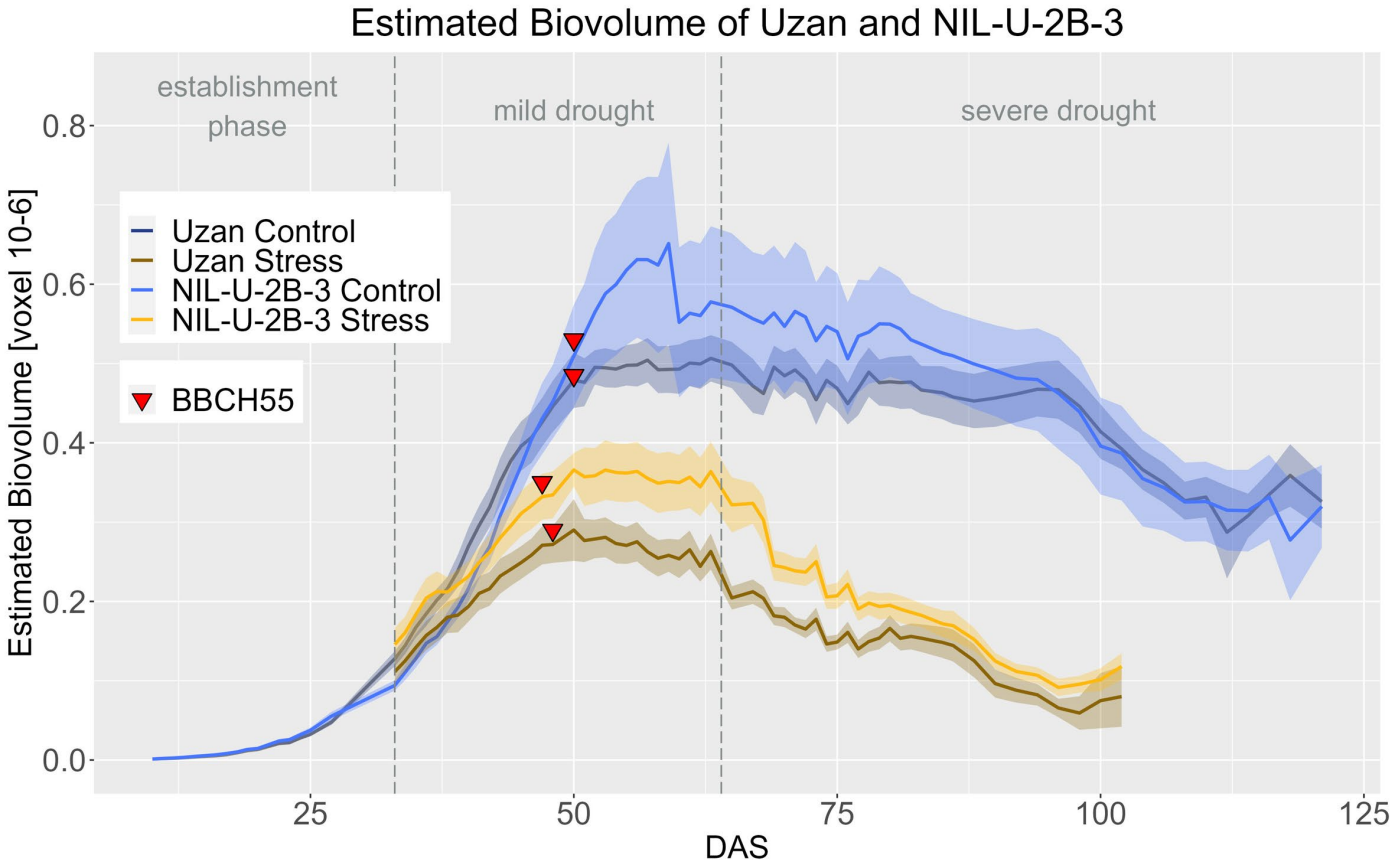
Estimated biovolume of Uzan and NIL-U-2B-3 based on the calculated BLUEs values. DAS, days after sowing; BBCH55, heading day. The last DAS for each genotype was chosen for the DAS where the last time 60% of the plants were images (as mature plants were taken off subsequently). The shadows describe the confidence interval; as long as the shadows of the individual lines do not overlap, the significance level of *a* = 0.05 is not reached and therefore a significant difference exists.

Regarding the effect of the 2B-QTL in the genetic background of Uzan, NIL-U-2B-3 produced significantly more EB compared to Uzan in the stress treatment during DAS 33–79 (Figure 10; Supplementary materials 8, 9). In the control treatment, the NIL also showed more EB, especially at DAS 48–90, but this difference was not significant. A treatment effect, i.e., a later response to drought in the biomass differences between control and stress treatment, of the 2B-QTL was also visible for the 2B-QTL, but less pronounced than in the 7A-QTL. From DAS 33 to 38 and from DAS 46 to harvest, the EB of NIL-U-2B-3 3 differed significantly in the two treatments, while in Uzan a significant difference between control and stress EB was observed from DAS 37 onwards (Supplementary material 10).

**Figure 11:**
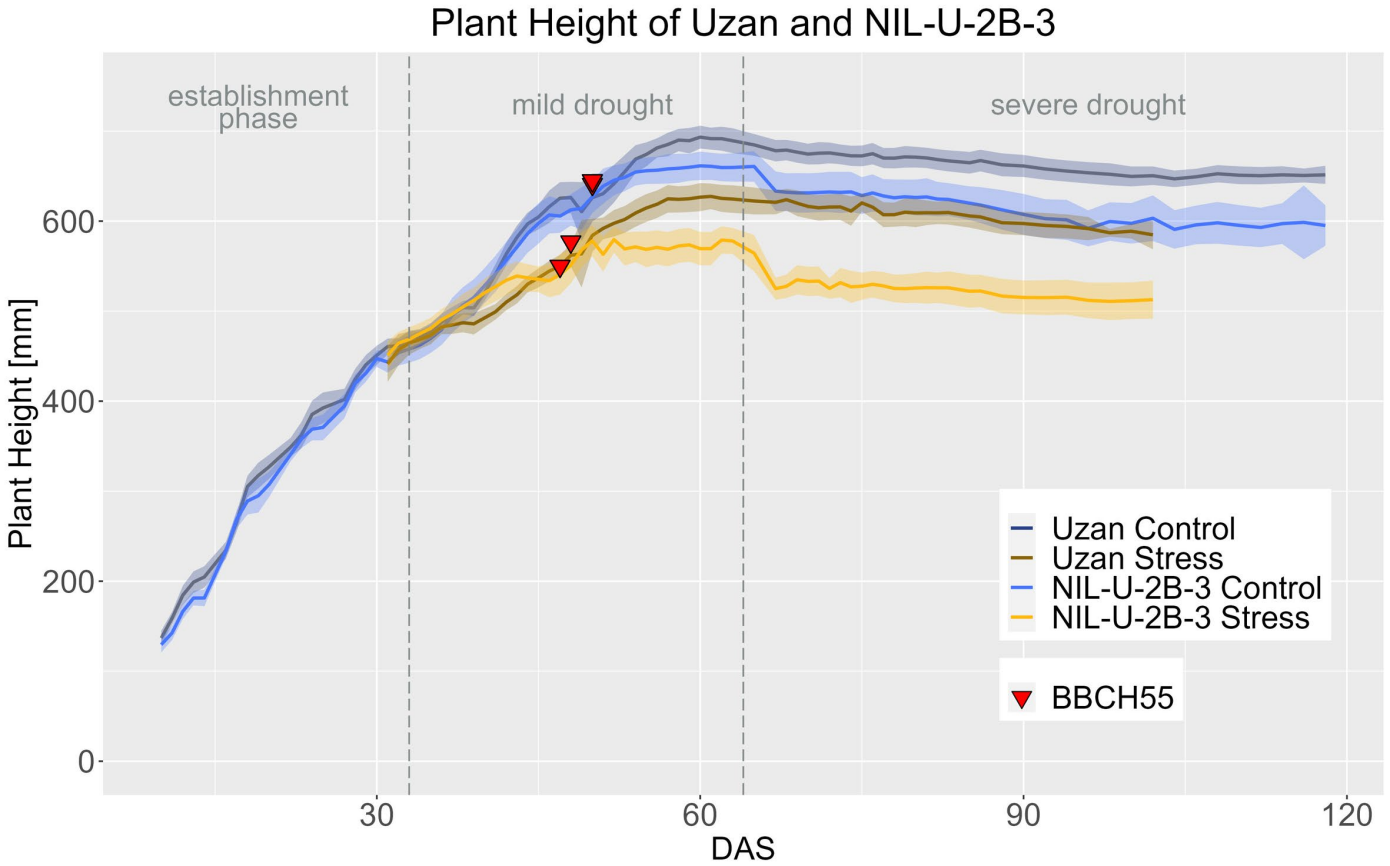
Plant height of Uzan and NIL-U-2B-3 based on calculated BLUEs values. DAS, days after sowing; BBCH55, heading day. The last DAS for each genotype was chosen for the DAS where the last time 60% of the plants were imaged (as mature plants were taken off subsequently). The shadows describe the confidence interval; as long as the shadows of the individual lines do not overlap, the significance level of *a* = 0.05 is not reached and therefore a significant difference exists.

In accordance with reduced PH at maturity (Table 4), the NIL-U-2B-3 remained smaller than its parent Uzan, especially in the period after heading (Figure 11; Supplementary materials 11–13). In the control treatment, the difference between the genotypes in PH was significant from DAS 58 onwards and in the stress treatment from DAS 53 on (Supplementary material 14). PH in Uzan differed significantly between control and stress treatment from DAS 40 onwards, while in the NIL this difference occurred 4 days later from DAS 44 onwards (Supplementary material 14), so the NIL responded later to drought concerning PH.

The 2B QTL showed a minor effect on the CVa. Uzan had a higher CVa during the mild drought phase than NIL-U-2B-3 (Figure 9; Supplementary materials 15, 16, 18). In the control treatment this difference was significant until DAS 90 and in stress treatment until DAS 76 (Supplementary material 15). The effect between the control and the stress treatment is for both Uzan and NIL-U-2B-3 significant from DAS 68 onwards (Supplementary material 17).

## Discussion

In the present study, the detailed effects of two QTLs introduced from wild emmer wheat for higher productivity with respect to spike and total dry matter under drought (chromosome 7A) or productivity with respect to grain yield across drought and control environments (chromosome 2B) were examined under well-watered and drought stress conditions using non-destructive HTP, for the first time throughout the whole plant life cycle. Our daily phenotyping results confirm advantageous effects of yield or yield parameters of the QTLs which were identified under field/screenhouse conditions in Israel and enabled us to determine the QTL effects on growth and their timing.

### Suitability of HTP phenotyping for evaluation of yield characteristics

The phenotyping platform used in this study has been used previously and has proven useful for estimating plant biomass up to the flowering stage under well-watered and drought stress conditions in barley (Neumann et al., 2015). As the plant material was from Israel, the chosen setup and greenhouse conditions aimed at mimicking the field situation in Israel, with a rising temperature gradient corresponding to the seasonal pattern and a slowly progressing drought reaching severe stress during grain filling.

With the application of an established standardized phenotyping protocol, high repeatability was achieved for EB and PH for each day throughout the life cycle under both, well-watered and drought stress conditions. Except for a few days, the data quality of the CVa was also high. The results of this study are in line with the high data quality throughout the vegetative growth period in well-watered and drought stress conditions on the same HTP system (Neumann et al. 2017; Dhanagond et al., 2019). The suitability of EB as a true proxy of biomass was also evident, in agreement with previous studies that were restricted to vegetative growth stages, though (Munns et al., 2010; Golzarian et al., 2011; Dhanagond et al., 2019; Shorinola et al., 2019). The results demonstrate the usefulness of this proxy over the entire plant life cycle. Based on the high-quality data set, statistical analyses to reveal the QTL effects throughout the life cycle could be performed.

Further, the data set allows to make statements about which imaging traits provide relevant information during or until which cereal developmental stage for HTP experiments. The EB increased dynamically until the milk ripening stage, while final PH was reached already shortly after flowering. After that, PH remained constant, while EB decreased with the ripening process due to the increase in mature plant parts, as these contain less water and thus the visible area is smaller. The CVa can be used to visualize and quantify the process of senescence of the plant by the changing plant color from green to yellow (Mikołajczak et al., 2020). If there is sufficient water available, the color of a plant is green and only changes with progressing senescence. However, drought stress can cause severe effects on plant pigments and changed the color of the plant when leaves were wilting during vegetative growth (Neumann et al., 2015). In the chosen setup of the current study of a slowly intensifying drought until maturity, the drought stressed plants started ripening earlier compared to the well-watered plants but showed no color changes during the vegetative phase with mild drought stress. The individual ripening curves reflected by the CVa of single plants are so clear that they represent a promising resource for the application of mathematic models. Curve modeling can add valuable insights into growth dynamics and allows the estimation of further traits. By growth curve modelling of vegetative biomass formation in barley, time points of maximal growth or start of wilting under drought could be estimated and resulted in the detection of corresponding QTL (Chen et al., 2014; Neumann et al. 2017; Dhanagond et al., 2019). By modeling the ripening curve based on CVa, it may be possible to quantify the ripening speed, which differed between genotypes and treatments and will shed more light into this phenology phase.

### General effects of drought stress during HTP experiment

The timing of drought significantly affects plant development. If drought stress occurs during the tillering stage, biomass, and the number of tillers per plant are reduced (Dhanagond et al., 2019). PH is affected when drought occurs during stem elongation (Ihsan et al., 2016) and the seed set is negatively affected by drought stress at the flowering stage (Sehgal et al., 2018). When drought stress occurs in the grain filling phase, TKW is reduced (V et al., 2019). As drought in our study started at the tillering stage and lasted until maturity, all these components were affected by progressing drought stress. Similar observations were made in a recent study in spring wheat (Fadoul et al., 2021).

In this HTP experiment, the plants of control and stress treatment started to show significant differences for EB and PH 7 days after the onset of drought. This is in accordance with previous observations in barley on the same HTP system, where biomass in control and drought treatment differed after 5 to 7 days (Neumann et al., 2015). The slower growth was accompanied by a decrease in tillering. Notably, the 10% loss in PH measured on DAS 47 was less than the 20–30% loss in tillering. Thus, the reduced EB may be mainly explained by reduced tillering rather than smaller plants. Similar losses caused by drought were also described in comparable HTP experiments (Honsdorf et al., 2014; Dhanagond et al., 2019; Pham et al., 2019). For the first time, a drought HTP experiment was conducted until maturity to simulate a natural progressing and intensifying drought. The long-term drought caused reductions of all yield components such as number of seeds, TKW and number of tillers and lead to faster maturity of stressed plants. Further, image capturing allowed to determine the heading date of each plant. Heading time is known to be affected by drought. Depending on the timing of the stress and its intensity, it can cause a delay in flowering (Chen et al., 2020; Gol et al., 2020) or lead to earlier flowering as a possible stress escape mechanism (Shavrukov et al., 2017). In the current study, there was only a small effect on heading time. This could be attributed to the fact that only mild drought was applied until flowering. However, a significant delay in the maturation date was observed in the NILs of 7A QTL, indicating that these plants are more resilient to drought. In addition, the Israeli plant material is already adapted to terminal drought and hardly changes their flowering time (Nevo et al., 2012).

### Comparison of HTP experiment with the field/screenhouse experiment

Heading time occurred much earlier in the greenhouse compared to the field. A significantly earlier flowering of up to 15 days in controlled environments compared to the field has recently been described in wheat (Sales et al., 2022). Still, the small but significant increasing effect of the 7A QTL in the BarNir background on heading time was detected also in the HTP experiment. The observed general lower yield parameters for single plants in pots compared to plants grown in plant stands in the field/screenhouse were to be expected. However, when averaging the two field years and comparing across all genotypes and both treatments, we reach between 62% (spikes per plant) and 87% (TKW) of the average trait values in the field for the yield parameters and 80% for culm length.

Besides the obvious differences of single plant growth in pots to growth in plant stands in a field soil, the growing conditions in terms of temperature, light quantity and quality, humidity and water availability are not the same. Though the HTP study incorporated a temperature gradient over the growing period, temperatures during day and night are stable in the climate-controlled greenhouse and do not show fluctuations as in the field and also the maxima in temperature reached in the two field years are technically not possible to reach in our greenhouse. Water availability is also difficult to compare between pot and field, due to the different ways of measuring in liters per pot in HTP and mm of precipitation in the field, which are two entirely different systems. According to Merchuk-Ovnat et al. (2016a), the applied water in the control treatment was 690–710 mm and in the water-limited 290-320 mm, which is about 42% lower precipitation in drought stress. In this HTP study, an average of 9 liters was watered to control plants and 3.2 liters to the drought stressed plants, corresponding to one third of the well-watered amount.

Strikingly, NIL-U2-B-3 in the Uzan background had an increasing effect on plant height, while having a decreasing effect in the HTP study in contrast. In general, the plant density in a pot experiment is much lower than in a field experiment. Poorter et al. (2016) analyzed in a meta-analysis of 100 trials how plant height is affected by the different plant densities in the different environments of pot and field. Genotypes planted in different environments showed no consistent trend in their height with respect to environment (Poorter et al., 2016). Thus, the difference in the 2B-QTL effect on plant height could attributed to the genotype and environment interaction. While the NIL-B-7A-2 was significantly taller than the parent BarNir in both HTP and field study, the effect was more pronounced in the HTP study. Similarly, the other observed differences in QTL effect occurrence and size can be interpreted as genotype x environment interaction effects.

### Dissection of wild emmer wheat QTL effects on shoot growth

In general, we could successfully reproduce the positive effects of the wild emmer wheat QTLs as had been observed in the previous field experiments. This demonstrates the suitability of the system and the applied setup to study complex traits such as drought resilience. The increased flag leaf area, photosynthesis and WUE of NIL-B-7A-2 in the HTP experiment have already been observed under stress and control treatments in the greenhouse and under control conditions in the field/screenhouse (Merchuk-Ovnat et al., 2016b). Furthermore, photosynthesis was measured both *via* gas exchange and with a portable device *via* photochemical quenching during sprouting and grain filling, and inferences were also made about WUE *via* carbon isotopes. In addition, the higher grain yield, TKW, and total dry matter of NIL-B-7A-2 (Merchuk-Ovnat et al., 2016a) were confirmed in the present experiment. However, the higher grain yield of NIL-Z-7A-2 in the control treatment in the field/screenhouse experiment (Merchuk-Ovnat et al., 2016a) was not observed in the HTP experiment, where TKW was increased under drought stress conditions.

The effect of the wild emmer wheat QTL on chromosome 7A revealed a mixed pattern in BarNir and Zahir backgrounds. For some traits it differed in both cultivars, while for others similar effects were observed. Thus, in both backgrounds a higher TKW was detected under drought stress conditions. In addition, a significant delay in the timing of senescence was observed in both based on CVa. This effect, known as ‘stay-green’ is advantageous under drought stress as it extends the photosynthetic activity, thus providing more assimilates for grain filling (Kamal et al., 2019). While ‘stay-green’ had a positive effect on TKW and yield in wheat mutants (Spano et al., 2003), a negative correlation of the late onset of senescence with yield was observed by Kipp et al. (2014). Non-invasive imaging methods such as those applied here, can visualize the ‘stay-green’ effect. In the field, the Normalized Difference Vegetation Index (NDVI) turned out to be suitable for its detection and could be modeled by a logistic model (Christopher et al., 2014). In a drought stress study it was possible to establish a clear relationship between higher yield and delayed senescence in wheat (Christopher et al., 2016; Rebetzke et al. 2016). The ‘stay-green’ effect was recently investigated in a GWAS study with sorghum based on several years of experiments with field-based drought stress in ten environments (Faye et al., 2021). It was detected that orthologs of the flowering genes in maize underlie the effect and thus cause an increased grain weight. HTP experiments until plant maturity offer the chance to investigate this effect in conjunction with other important traits to gain a holistic understanding of potential trade-offs.

The effect of the 7A-QTL may bear on the production and distribution of hormones at the time of grain filling. In the study of the ‘stay green’ phenotype in wheat, an association with altered cytokinin metabolism and the hormone ABA has already been established (Wang et al., 2016). In a previous transcriptome study of drought effect in drought resilient vs. susceptible wild emmer wheat accessions, the involvement of plant hormones, mainly ABA, GA, IAA, and prolonged metabolic activity were associated with drought resilience (Krugman et al., 2010, 2011).

In the background of BarNir the 7A QTL also resulted in significantly greater EB in both treatments from the seedling stage on, which was connected with larger plants, higher photosynthetic rate, and an improved WUE. The slightly lower CVa observed during a phase when all other plants appeared normally green could be related to epicuticle growth. A link between leaf color and wax content has been demonstrated in oilseed rape by overexpressing the lipid transfer protein gene *BraLTP1* and in Spanish juniper (*Juniperus thurifera*) by measuring the leaf reflectance of green and glaucous leaves with a spectroradiometer (Esteban et al., 2014; Liu et al., 2014). Several studies have identified a relationship between epicuticular waxes and reduced transpiration and higher photosynthesis under drought stress (Guo et al., 2016; Bi et al., 2017). Future studies should evaluate epicuticular waxes on the leaf surface in more detail in addition to plant hormones in BarNir and NIL-B-7A-2.

Since these effects in the BarNir background were not found in the Zahir background, the origin of the QTL was scrutinized. The QTLs were selected from a RIL population originating from a cross between wild emmer wheat and the durum wheat cultivar Langdon. The selection of flanking markers for the QTLs was based on a DArT map (Peleg et al. 2008; Merchuk-Ovnat et al., 2016a). In 2020, another genetic map was created based on 15 K SNP array for the RIL 12 and NIL-B-7A-2 (Deblieck et al., 2020; Fatiukha et al., 2021). Based on this new map, that included 4,015 SNPs, it was found that genetic material from Langdon is also present in the selected QTL region.

Therefore, there is the potential that the differences of the QTL effects in NIL-B-7A-2 compared to NIL-Z-7A-5 arise from the Langdon fragment. Different effects of introgressed QTLs in different genetic backgrounds are not only caused by genotype-environment interactions, but are also due to the different backgrounds (Muellner et al., 2020; Ollier et al., 2020).

The 2B QTL showed a positive effect on the development of EB from flowering to maturity in stress treatment. In principle, there is a clear correlation between a higher number of tillers and a higher yield (Naruoka et al., 2011). However, under stress conditions, increased tillering can be a disadvantage for the plants because not all shoots form fertile ears (Wang Z. et al., 2016; Fábián et al., 2019). Nevertheless, NIL-U-2B-3, also showed a higher number of spikes in both treatments and a higher number of fertile spikes under drought. Higher grain weight, as observed in the field/screenhouse, could not be confirmed here, which may be due to genotype-environment interactions (Merchuk-Ovnat 2016a). Although the plant grain weight was not higher under any treatment in NIL-U-2B-3 in the HTP experiment, the WUE and the HI were increased under drought. The higher tillering and WUE may be related to abscisic acid (ABA; Wang et al., 2018; Itam et al., 2020). The plant hormone ABA is involved in many metabolic pathways. It is an important regulator of water use because it directly regulates stomatal aperture, thereby affecting transpiration (Dunn et al., 2019; Mega et al., 2019). CIPK genes play an important role as they mediate between the ABA signaling pathway and drought stress responses (Cui et al., 2018). Sensitivity for ABA should be tested in the future.

The fact that a higher grain weight was not achieved despite the increased number of fertile ears may be explained by the source-sink relationship. Although more EB or leaf area is available as a source and more fertile ears as sinks, the process of filling the grains was nevertheless interrupted by drought stress or earlier onset of senescence, which interrupts photosynthesis.

Presumably, many resources were invested in tillering, which is far higher for single plants than for plants in field stands, leaving fewer resources in the form of water-soluble carbohydrates available for grain filling (Abdelrahman et al., 2020).

## Conclusion

This study demonstrates that non-invasive imaging under controlled conditions and a well-chosen setup can shed light on complex traits such as yield formation under drought even with the drawbacks of a pot experiment. For the first time, an HTP experiment was conducted over the whole plant life cycle in wheat and was able to not only confirm the effect of improved yield and dry matter of two wild emmer wheat QTLs introgressed into Israeli wheat cultivars but also resulted in further insights of their effects during plant development and their temporal dynamics. Thus, it is clear that HTP and field experiments can be combined to complement elucidation of intogressed QTLs in NILs, as in this study, and serve to further decipher mechanisms. Lessons from this experiment can also be drawn with respect to the useful phenotyping period for traits such as PH and EB. Maximum PH and EB were reached about a week after heading, so experiments that only aim at exploring these traits can be stopped at that time. To obtain information on different onset and progress of senescence, the evaluation period should be extended, accordingly. Here, the CVa curves represented best the ongoing senescence of plants. This process can be subjected to modelling of growth curves to obtain parameters for the rate of maturation as the curves of CVa of individual plants are very clear at that time.

The effects of beneficial QTLs of wild emmer wheat in drought and also in control conditions demonstrate the importance of using wild alleles for crop improvement. The differences in the effect of the 7A QTL in the two genetic backgrounds need to be further evaluated in the future. Since the effect of wild emmer wheat QTLs was confirmed in field/screenhouse and pot trials, the NILs were recently crossed with elite German cultivars for future research.

## Data availability statement

The raw data supporting the conclusions of this article will be made available by the authors, without undue reservation.

## Author contributions

YS and KN designed the study. KN conducted the experiment. ML analyzed the precision phenotyping data and was together with KN the major contributor in writing the manuscript. YS supported in statistical analysis of the phenotypic data. CK supported in image analysis and developed an update of the image analysis software IAP. YS and TK developed the material and together with AG, MD, DP, and FO supported in interpretation of the results about QTL effects. All authors contributed to the article and approved the submitted version.

## Funding

This study was supported by the Israel Ministry of Agriculture and Rural Development, Chief Scientist Foundation (Grants 837-0079-10, 837-0162-14) and the German Federal Ministry of Food and Agriculture (FKZ: 2813IL03). YS is the incumbent of the Haim Gvati Chair in Agriculture. This work was conducted as part of the STARGATE project funded by the European Union’s Horizon 2020 Research and Innovation Program under grant agreement number 952339.

## Conflict of interest

CK was employed by company BASF SE.

The remaining authors declare that the research was conducted in the absence of any commercial or financial relationships that could be construed as a potential conflict of interest.

## Publisher’s note

All claims expressed in this article are solely those of the authors and do not necessarily represent those of their affiliated organizations, or those of the publisher, the editors and the reviewers. Any product that may be evaluated in this article, or claim that may be made by its manufacturer, is not guaranteed or endorsed by the publisher.
