# Supplementary material for: Precision phenotyping across the life cycle to validate and decipher drought-adaptive QTLs of wild emmer wheat (Triticum turgidum ssp. dicoccoides) introduced into elite wheat varieties
Source: Front Plant Sci. 2022 Oct 12;13:965287. doi: 10.3389/fpls.2022.965287 (PMC9598872; doi:10.3389/fpls.2022.965287)
Supplement: Supplementary file 1 [file Table_1.docx]

**Supplementary Material**

[Supplementary Material 1: Time course of actions during HTP experiment. 41](#_Toc109856288)

[Supplementary Material 2: Comparison of control and stress treatment for all genotypes (parents and corresponding NILs) in the HTP experiment using t-test. 42](#_Toc109856289)

[Supplementary Material 3: Descriptive statistics based on BLUEs for all genotypes (parents and corresponding NILs) for selected days in HTP experiment. 43](#_Toc109856290)

[Supplementary Material 4: Average based on BLUEs for all genotypes (parents and corresponding NILs) of traits measured before and at maturity in the HTP experiment. 44](#_Toc109856291)

[Supplementary Material 5: Distribution of Heading (BBCH55) in DAS. 46](#_Toc109856292)

[Supplementary Material 6: DAS to maturity. 47](#_Toc109856293)

Supplementary Material 7: Comparison of the percentage increase in performance of the NILs compared to the parents of common traits of the HTP experiment and field/screenhouse experiments…………………9

Supplementary Material 8: p-values of a comparison of parent vs NIL for estimated biovolume using t-test………………………………………………………………………………………………………….12

Supplementary Material 9: Descriptive statistics for the trait estimated biomass for selected days in the HTP experiment. ………………………………………………………………………………………………...13

Supplementary Material 10: p-values of a comparison of control vs stress treatment for each genotype for Estimated Biovolume using t-test…………………………………………………………………………..15

Supplementary Material 11: p-values of a comparison of parent vs NIL during plant establishment phase using t-test for estimated biovolume, plant height and the color value……………………………………...16

Supplementary Material 12: p-values of a comparison of parent vs NIL for plant height using t-test………17

Supplementary Material 13: Descriptive statistics for the trait plant height on selected days in the HTP experiment. ………………………………………………………………………………………………..18

Supplementary Material 14: p-values of a comparison of control vs stress treatment for plant height using t-test………………………………………………………………………………………………………….20

Supplementary Material 15: p-values of a comparison of parent vs NIL for color value using t-test……….21

Supplementary Material 16: Descriptive statistics for the trait color value for selected days in the HTP experiment. ………………………………………………………………………………………………..22

Supplementary Material 17: p-values of a comparison of control vs stress treatment for color value using t-test………………………………………………………………………………………………………….24

Supplementary Material 18: Descriptive statistics for the trait color value for selected days in the HTP experiment. ………………………………………………………………………………………………..25

**Supplementary Material 1:** Time course of actions during HTP experiment.

| Action | Date | DAS | Temperature |
| --- | --- | --- | --- |
| Sowing with two seeds per pot | 05.07.2019 | 0 | 16/12°C, 15h light |
| Thinning out to one plant per pot | 16.07.2019 | 11 |  |
| First side view imaging | 17.07.2019 | 12 |  |
| Tiller count 1 | 02.08.2019 | 28 |  |
| Initiation of drought stress down to 30% PAW | 05.08.2019 | 31 | 20/16°C, 15h light |
| Tiller count 2 | 27.08.2019 | 53 |  |
| Temperature increased | 05.09.2019 | 62 | 24/20°C, 15h light |
| Initiation of drought stress down to 20% PAW | 07.09.2019 | 64 |  |
| Flag leave measurement and sampling for Osmolarity | 10.09.2019 | 67 |  |
| Chlorophyll Fluorescence Imaging 1 | 12.09.2019 | 69 |  |
| Tiller count 3 | 13.09.2019 | 70 |  |
| Chlorophyll Fluorescence Imaging 2 | 17.09.2019 | 74 |  |
| Last imaging | 05.11.2019 | 123 |  |

**Supplementary Material 2:** Comparison of control and stress treatment for all genotypes (parents and corresponding NILs) in the HTP experiment using t-test.

| **Estimated Biovolume (voxel 10^6^)** | | | | |  |  |  |  |  |  |  |  |  |  |  |  |  |  |  |  |
| --- | --- | --- | --- | --- | --- | --- | --- | --- | --- | --- | --- | --- | --- | --- | --- | --- | --- | --- | --- | --- |
| **DAS** | **33** | **34** | **35** | **36** | **37** | **38** | **39** | **40** | **41** | **42** | **43** | **44** | **45** | **46** | **47** | **48** | **50** | **51** | **52** | **53** |
| **p-value** | 0.62 | 0.45 | 0.34 | 0.21 | 0.12 | 0.03 | 0.00 | 0.00 | 0.00 | 0.00 | 0.00 | 0.00 | 0.00 | 0.00 | 0.00 | 0.00 | 0.00 | 0.00 | 0.00 | 0.00 |
| **DAS** | **54** | **55** | **56** | **57** | **58** | **59** | **60** | **61** | **62** | **63** | **65** | **67** | **68** | **69** | **70** | **71** | **72** | **73** | **74** | **75** |
| **p-value** | 0.00 | 0.00 | 0.00 | 0.00 | 0.00 | 0.00 | 0.00 | 0.00 | 0.00 | 0.00 | 0.00 | 0.00 | 0.00 | 0.00 | 0.00 | 0.00 | 0.00 | 0.00 | 0.00 | 0.00 |
| **DAS** | **76** | **77** | **78** | **79** | **80** | **81** | **82** | **83** | **85** | **86** | **88** | **90** | **92** | **94** | **96** |  |  |  |  |  |
| **p-value** | 0.00 | 0.00 | 0.00 | 0.00 | 0.00 | 0.00 | 0.00 | 0.00 | 0.00 | 0.00 | 0.00 | 0.00 | 0.00 | 0.00 | 0.00 |  |  |  |  |  |
|  |  |  |  |  |  |  |  |  |  |  |  |  |  |  |  |  |  |  |  |  |
| **Plant Height (mm)** | | | |  |  |  |  |  |  |  |  |  |  |  |  |  |  |  |  |  |
| **DAS** | **31** | **32** | **33** | **34** | **35** | **36** | **37** | **38** | **39** | **40** | **41** | **42** | **43** | **44** | **45** | **46** | **47** | **48** | **49** | **50** |
| **p-value** | 0.33 | 0.74 | 0.99 | 0.90 | 0.54 | 0.21 | 0.14 | 0.03 | 0.01 | 0.00 | 0.00 | 0.00 | 0.00 | 0.00 | 0.00 | 0.00 | 0.00 | 0.00 | 0.00 | 0.00 |
| **DAS** | **51** | **52** | **53** | **54** | **55** | **56** | **57** | **58** | **59** | **60** | **61** | **62** | **63** | **65** | **67** | **68** | **69** | **70** | **71** | **72** |
| **p-value** | 0.00 | 0.00 | 0.00 | 0.00 | 0.00 | 0.00 | 0.00 | 0.00 | 0.00 | 0.00 | 0.00 | 0.00 | 0.00 | 0.00 | 0.00 | 0.00 | 0.00 | 0.00 | 0.00 | 0.00 |
| **DAS** | **73** | **74** | **75** | **76** | **77** | **78** | **79** | **80** | **81** | **82** | **83** | **85** | **86** | **88** | **90** | **92** | **94** | **96** |  |  |
| **p-value** | 0.00 | 0.00 | 0.00 | 0.00 | 0.00 | 0.00 | 0.00 | 0.00 | 0.00 | 0.00 | 0.00 | 0.00 | 0.00 | 0.00 | 0.00 | 0.00 | 0.00 | 0.00 |  |  |
|  |  |  |  |  |  |  |  |  |  |  |  |  |  |  |  |  |  |  |  |  |
| **Color Value (hue)** | | |  |  |  |  |  |  |  |  |  |  |  |  |  |  |  |  |  |  |
| **DAS** | **31** | **32** | **33** | **34** | **35** | **36** | **37** | **38** | **39** | **40** | **41** | **42** | **43** | **44** | **45** | **46** | **47** | **48** | **49** | **50** |
| **p-value** | 0.71 | 0.80 | 0.37 | 0.67 | 0.43 | 0.68 | 0.70 | 0.82 | 0.60 | 0.89 | 0.70 | 0.66 | 0.93 | 0.96 | 0.84 | 0.86 | 0.62 | 0.53 | 0.45 | 0.12 |
| **DAS** | **51** | **52** | **53** | **54** | **55** | **56** | **57** | **58** | **59** | **60** | **61** | **62** | **63** | **65** | **67** | **68** | **69** | **70** | **71** | **72** |
| **p-value** | 0.07 | 0.77 | 0.75 | 0.98 | 0.79 | 0.65 | 0.43 | 0.83 | 0.85 | 0.44 | 0.51 | 0.03 | 0.52 | 0.10 | 0.00 | 0.00 | 0.00 | 0.00 | 0.00 | 0.00 |
| **DAS** | **73** | **74** | **75** | **76** | **77** | **78** | **79** | **80** | **81** | **82** | **83** | **85** | **86** | **88** | **90** | **92** | **94** | **96** |  |  |
| **p-value** | 0.00 | 0.00 | 0.00 | 0.00 | 0.00 | 0.00 | 0.00 | 0.00 | 0.00 | 0.00 | 0.00 | 0.00 | 0.00 | 0.00 | 0.00 | 0.00 | 0.00 | 0.00 |  |  |

Significant differences calculated with a two-sided Student's t-test (p<0.05) are highlighted in green. The drought started at DAS 31 but due to technical issues with top view imaging in that period, this trait could only be compared from DAS 33 onwards.

**Supplementary Material 3:** Descriptive statistics based on BLUEs for all genotypes (parents and corresponding NILs) for selected days in HTP experiment.

| **Genotype** | | **Estimated Biovolume** | | | **Plant Height** | | | **Color Value** | | |
| --- | --- | --- | --- | --- | --- | --- | --- | --- | --- | --- |
|  | | **(Voxel 10^6^)** | | | **(mm)** | | | **(hue)** | | |
| **Measurement** | | **Control** | **Stress** | **Loss (%)** | **Control** | **Stress** | **Loss (%)** | **Control** | **Stress** | **Loss (%)** |
| **DAS33** | **Av** | **1.24** | **1.21** | **2** | **451.3** | **451.4** | **0** | **0.23** | **0.23** | **0** |
|  | **SD** | 0.41 | 0.45 | -10 | 44.5 | 50.9 | -14 | 0.004 | 0.004 | 0 |
|  | **Min** | 0.53 | 0.54 | -2 | 317.6 | 338.8 | -7 | 0.22 | 0.219 | 0 |
|  | **Max** | 2.21 | 2.13 | 4 | 547 | 570.4 | -4 | 0.238 | 0.238 | 0 |
| **DAS40** | **Av** | **2.71** | **2.13** | **21** | **519.9** | **493.3** | **5** | **0.232** | **0.232** | **0** |
|  | **SD** | 0.87 | 0.69 | 21 | 47.7 | 47.3 | 1 | 0.003 | 0.003 | 0 |
|  | **Min** | 1.42 | 1.17 | 18 | 425.1 | 396.7 | 7 | 0.225 | 0.226 | 0 |
|  | **Max** | 4.98 | 4.08 | 18 | 657.1 | 611.6 | 7 | 0.238 | 0.238 | 0 |
| **DAS47** | **Av** | **4.45** | **2.71** | **39** | **591.8** | **535.5** | **10** | **0.231** | **0.23** | **0** |
|  | **SD** | 1.42 | 0.88 | 38 | 72 | 68.1 | 5 | 0.003 | 0.003 | 0 |
|  | **Min** | 2.3 | 1.46 | 37 | 439.2 | 403.9 | 8 | 0.223 | 0.225 | -1 |
|  | **Max** | 8.78 | 4.8 | 45 | 743.4 | 743.3 | 0 | 0.237 | 0.235 | 1 |
| **DAS54** | **Av** | **5.47** | **2.95** | **46** | **678** | **603.3** | **11** | **0.227** | **0.227** | **0** |
|  | **SD** | 2.01 | 0.93 | 54 | 92.5 | 86.3 | 7 | 0.003 | 0.003 | 0 |
|  | **Min** | 2.63 | 1.25 | 52 | 496 | 449.3 | 9 | 0.219 | 0.219 | 0 |
|  | **Max** | 11.85 | 5.22 | 56 | 897 | 782.2 | 13 | 0.234 | 0.233 | 0 |
| **DAS70** | **Av** | **5.14** | **2.11** | **59** | **707** | **634.1** | **10** | **0.214** | **0.204** | **5** |
|  | **SD** | 2.12 | 0.66 | 69 | 112.8 | 108.2 | 4 | 0.006 | 0.01 | NA |
|  | **Min** | 1.81 | 1.22 | 33 | 491.1 | 452.9 | 8 | 0.203 | 0.179 | 12 |
|  | **Max** | 10.93 | 3.68 | 66 | 885 | 825 | 7 | 0.222 | 0.221 | 0 |
| **DAS80** | **Av** | **5.52** | **2.05** | **63** | **699.6** | **626.6** | **10** | **0.201** | **0.171** | **15** |
|  | **SD** | 2.38 | 0.75 | 68 | 108.9 | 112.4 | -3 | 0.012 | 0.018 | NA |
|  | **Min** | 2.48 | 1.25 | 50 | 484.5 | 436.6 | 10 | 0.183 | 0.132 | 28 |
|  | **Max** | 12.97 | 4.43 | 66 | 878.7 | 821.1 | 7 | 0.22 | 0.207 | 6 |
| **DAS90** | **Av** | **5.42** | **1.6** | **70** | **680.4** | **604.4** | **11** | **0.203** | **0.116** | **43** |
|  | **SD** | 2.37 | 0.71 | 70 | 113.1 | 106.3 | 6 | 0.008 | 0.034 | NA |
|  | **Min** | 2.45 | 0.53 | 78 | 470.3 | 420.2 | 11 | 0.177 | 0.069 | 61 |
|  | **Max** | 14.73 | 3.56 | 76 | 872.4 | 787.3 | 10 | 0.214 | 0.179 | 16 |

Av = Average, SD = Standard deviation, Min = Minimum, Max = Maximum. The loss is calculated by the percentage loss from the stress treatment compared to the control treatment.

**Supplementary Material 4:** Average based on BLUEs for all genotypes (parents and corresponding NILs) of traits measured before and at maturity in the HTP experiment.

| **Traits at maturity** | **Control** | **Stress** | **Loss (%)** |  | **Traits before maturity** | **Control** | **Stress** | **Loss (%)** |
| --- | --- | --- | --- | --- | --- | --- | --- | --- |
| Main Ear Awn Length (cm) | 8.4 ** | 7.84 | 7 |  | TN DAS28 | 2.68 | 2.55 | 5 |
| Ear Length (cm) | 8.4 | 8.45 | -1 |  | TN DAS53 | 4.47 ** | 3.47 | 22 |
| Peduncle length (cm) | 12.01 | 11.51 | 4 |  | TN DAS70 | 5.73 ** | 3.9 | 32 |
| Last Internode length (cm) | 16.76 ** | 15.29 | 9 |  | Gain of TN DAS28 and 53 | 1.78 ** | 0.92 | 48 |
| Culm length (cm) | 55.71 | 50.75 | 9 |  | Gain of TN DAS53 and 70 | 1.15 ** | 0.43 | 63 |
| Plant height (cm) | 64.03 ** | 59.17 | 8 |  | Gain of TN DAS28 and 70 | 3.05 ** | 1.35 | 56 |
| Number of Spikes | 5.53 ** | 3.22 | 42 |  | BBCH55 in DAS | 50.17 ** | 47.96 | 4 |
| Number of fertile Spikes | 5.33 ** | 3.18 | 40 |  | Flag Leaf Width (mm) | 17.1 ** | 16.08 | 6 |
| Plant Biomass (g) | 13.22 ** | 5.96 | 55 |  | Flag Leaf Length (cm) | 28.27 ** | 24.57 | 13 |
| Plant Grain Weight (g) | 8.19 ** | 4.32 | 47 |  | Flag Leaf Area (cm²) | 357.3 ** | 295.2 | 17 |
| Plant Straw Weight (g) | 7.25 ** | 3.6 | 50 |  | Osmotic Potential (MPa) | -1.47 ** | -1.81 | -23 |
| Plant Harvest Index | 0.48 ** | 0.45 | 6 |  | QY-H DAS 69 | 0.46 ** | 0.44 | 4 |
| Biomass WUE (g/l) | 1.45 ** | 1.87 | -29 |  | QY-H DAS 74 | 0.46 ** | 0.43 | 7 |
| Plant TKW (g) | 46.06 | 41.35 | 10 |  | QY-L DAS 69 | 0.52 ** | 0.5 | 4 |
| Plant Seed area (mm^2^) | 16.6 ** | 15.24 | 8 |  | QY-L DAS 74 | 0.52 ** | 0.49 | 6 |
| Plant Seed width (mm) | 3.62 ** | 3.44 | 5 |  | QY-LH Ratio DAS 69 | 1.11 ** | 1.14 | -3 |
| Plant Seed Length (mm) | 6.36 ** | 6.17 | 3 |  | QY-LH Ratio DAS 74 | 1.14 | 1.15 | -1 |
| Plant Grain Number | 176.6 ** | 105.23 | 40 |  |  |  |  |  |
| Grains per Ear | 34.27 | 34.2 | 0 |  |  |  |  |  |
| Main Ear Spikelet Number | 18.72 | 18.4 | 2 |  |  |  |  |  |
| Main Ear Grain Number | 38.18 | 38.11 | 0 |  |  |  |  |  |
| Main Ear Grains per Spikelet | 2.06 | 2.1 | -2 |  |  |  |  |  |
| Main Ear Biomass (g) | 2.21 ** | 1.96 | 11 |  |  |  |  |  |
| Main Ear Grain Weight (g) | 1.84 ** | 1.6 | 13 |  |  |  |  |  |
| Main Ear Straw (g) | 0.39 ** | 0.35 | 10 |  |  |  |  |  |
| Main Ear Harvest Index | 0.83 | 0.82 | 1 |  |  |  |  |  |
| Main Ear TKW (g) | 48.05 ** | 43.79 | 9 |  |  |  |  |  |
| Main Ear Seed Area (mm^2^) | 16.75 ** | 15.98 | 5 |  |  |  |  |  |
| Watersum (l) | 8.95 | 3.16 | 65 |  |  |  |  |  |

Significant differences calculated with a two-sided Student's t-test (p<0.05) are marked with: * p<0.02 and ** p<0.001. TN = tiller number, QY-H = quantum yield of photosystem II under high-light and QY-L under low light, QY-LH = ratio of QY-L to QY-H, DAS = days after sowing. The loss is calculated by the percentage loss from the stress treatment compared to the control treatment. Negative losses result from drought stress

**Supplementary Material 5:** Violin plots for Heading time (BBCH55) in DAS.

a.) for the treatments, b.) for the genotypes in control treatment and c.) for the genotypes in stress treatment.

**
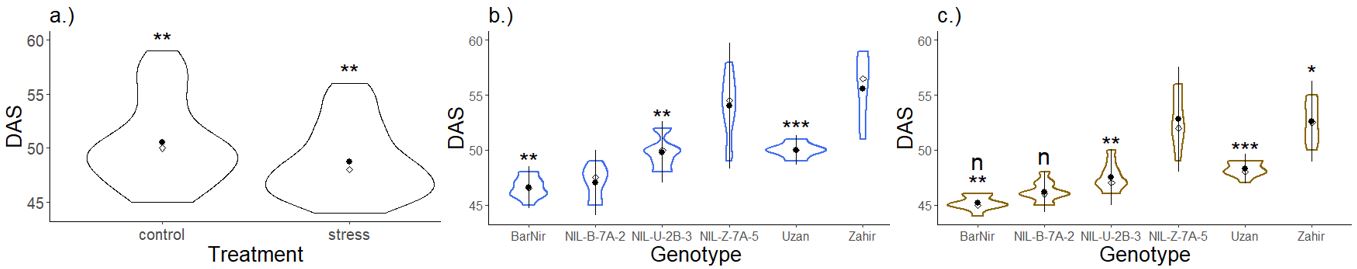
** Two-tailed Student's t-test were performed to detect differences between genotypes in control and stress and marked with p<0.05 *, 0.01 ** and 0.001 ***, respectively. Significant differences between cultivars and NILs are marked with an n for p<0.05..

**Supplementary Material 6:** Violin plots for DAS to maturity.

a.) Comparison of the two treatments and b) Comparison of the genotypes in stress treatment.

**
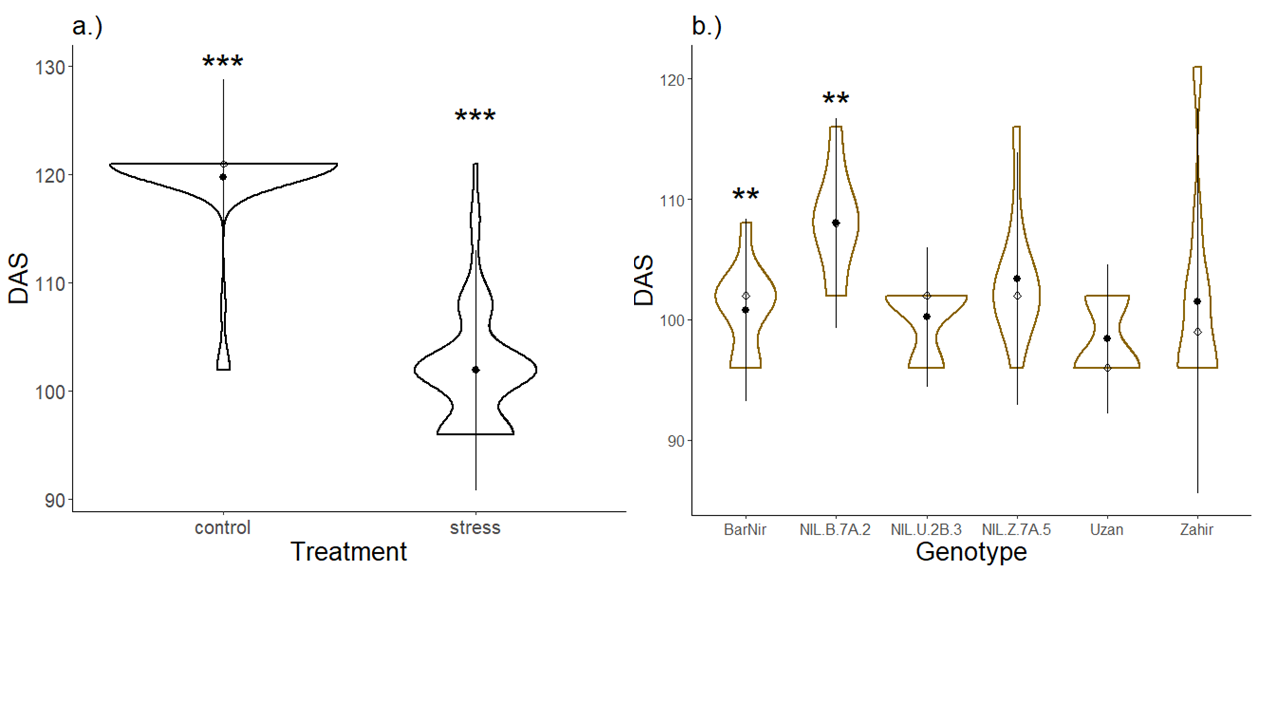
** Two-tailed Student's t-test were performed to detect differences between genotypes in control and stress and marked with p<0.05 *, 0.01 ** and 0.001 ***, respectively.

**Supplementary Material 7:** Comparison of the percentage increase in performance of the NILs compared to the parents of common traits of the HTP experiment and field/screenhouse experiments.

|  |  | **% NIL-B-7A-2 of BarNir** | | | | | | **% NIL-Z-7A-5 of Zahir** | | | | | | **% NIL-U-2B-3 of Uzan** | | | |
| --- | --- | --- | --- | --- | --- | --- | --- | --- | --- | --- | --- | --- | --- | --- | --- | --- | --- |
| **Trait** | **Treatment** | **in HTP** | | **in Field Y1** | | **in Field Y2** | | **in HTP** | | **in Field Y1** | | **in Field Y2** | | **in HTP** | | **in Field Y2** | |
| Culm Length(cm) | Control | 172 | *** | 125 | *** | 119 | *** | 104 |  | 112 | ** | 112 | *** | 87 | *** | 111 | * |
|  | Stress | 172 | *** | 125 | *** | 124 | *** | 98 |  | 109 | * | 106 | * | 84 | *** | 111 | * |
| BBCH55 | Control | 101 |  | 103 | *** | 106 | *** | 97 |  | 111 | *** | 110 | *** | 100 |  | 101 |  |
|  | Stress | 102 | ** | 103 | *** | 106 | *** | 100 |  | 115 | *** | 114 | *** | 98 |  | 103 |  |
| Grains per Ear | Control | 104 |  | 97 |  | 90 |  | 87 |  | 106 |  | 94 |  | 94 |  | 85 |  |
|  | Stress | 99 |  | 107 |  | 104 |  | 85 | ** | 113 |  | 95 |  | 86 | * | 112 |  |
| Grain Yield (g) | Control | 138 |  | 119 |  | 89 |  | 86 |  | 142 | ** | 134 | * | 107 |  | 148 | * |
|  | Stress | 143 | *** | 159 | * | 162 | * | 100 |  | 132 |  | 136 |  | 113 |  | 256 | * |
| Harvest Index | Control | 100 |  | 98 |  | 108 |  | 98 |  | 98 |  | 96 |  | 105 |  | 94 |  |
|  | Stress | 98 |  | 108 |  | 96 |  | 100 |  | 94 |  | 93 |  | 110 | * | 116 |  |
| Osmotic Potential (Mpa) | Control | 99 |  | 95 |  | 106 |  | 103 |  | 101 |  | 103 |  | 103 |  | 102 |  |
|  | Stress | 102 |  | 91 |  | 114 | *** | 99 |  | 105 |  | 96 |  | 104 |  | 100 |  |
| Number of spikes | Control | 101 |  | 106 |  | 86 |  | 98 |  | 123 |  | 132 | * | 119 | * | 160 | *** |
|  | Stress | 114 |  | 126 |  | 142 |  | 96 |  | 125 | * | 143 | * | 127 | *** | 149 |  |
| TKW | Control | 120 | *** | 112 | ** | 101 |  | 100 |  | 101 |  | 107 |  | 97 |  | 109 | * |
|  | Stress | 119 | *** | 115 | ** | 105 |  | 115 | * | 101 |  | 101 |  | 100 |  | 111 | ** |
| Plant Biomass (g) | Control | 135 | * | 121 |  | 96 |  | 89 |  | 137 | ** | 140 | * | 107 |  | 156 | * |
|  | Stress | 149 | *** | 152 | * | 167 | * | 103 |  | 141 |  | 145 |  | 114 |  | 224 |  |

The percentage comparison is calculated as the mean of the NIL / mean of the parent * 100.

The values for the field/screenhouse are taken from Merchuk-Ovnat (2016a; Table 3, TableS4 and Table S5). HTP = High-throughput Phenotyping Experiment; Field Y = field/screenhouse experiment Year 1 and 2; BBCH55=heading. Significance data is from the original tables of Merchuck-Ovnat et al. (2016a) and Table 3 and Table 4. Mean comparisons by Student's t-test between each line and its recurrent parent (*, **, ***) under control and drought stress treatments at p< 0.05, 0.01, 0.001, respectively

**Supplementary Material 8:** p-values of a comparison of parent vs NIL for estimated biovolume using t-test

| **Combination** | **DAS** | **33** | **34** | **35** | **36** | **37** | **38** | **39** | **40** | **41** | **42** | **43** | **44** | **45** | **46** | **47** | **48** | **50** | **51** | **52** | **53** | **54** | **55** |
| --- | --- | --- | --- | --- | --- | --- | --- | --- | --- | --- | --- | --- | --- | --- | --- | --- | --- | --- | --- | --- | --- | --- | --- |
| **BarNir vs.** | Control | 0.38 | 0.36 | 0.25 | 0.27 | 0.10 | 0.10 | 0.02 | 0.06 | 0.08 | 0.05 | 0.05 | 0.04 | 0.00 | 0.00 | 0.00 | 0.00 | 0.00 | 0.00 | 0.01 | 0.00 | 0.00 | 0.00 |
| **NIL-B-7A-2** | Stress | 0.01 | 0.00 | 0.00 | 0.00 | 0.00 | 0.00 | 0.00 | 0.00 | 0.00 | 0.00 | 0.00 | 0.00 | 0.00 | 0.00 | 0.00 | 0.00 | 0.00 | 0.00 | 0.00 | 0.00 | 0.00 | 0.00 |
| **Zahir vs.** | Control | 0.01 | 0.01 | 0.01 | 0.01 | 0.01 | 0.01 | 0.01 | 0.01 | 0.03 | 0.04 | 0.05 | 0.07 | 0.11 | 0.14 | 0.26 | 0.32 | 0.74 | 0.48 | 0.49 | 0.56 | 0.53 | 0.49 |
| **NIL-Z-7A-5** | Stress | 0.86 | 0.61 | 0.71 | 0.67 | 0.95 | 0.83 | 0.77 | 0.67 | 0.74 | 0.87 | 0.84 | 0.77 | 0.72 | 0.75 | 0.88 | 0.57 | 0.96 | 0.54 | 0.61 | 0.59 | 0.51 | 0.47 |
| **Uzan vs.** | Control | 0.00 | 0.00 | 0.00 | 0.01 | 0.00 | 0.00 | 0.01 | 0.01 | 0.03 | 0.03 | 0.10 | 0.20 | 0.42 | 0.95 | 0.90 | 0.88 | 0.54 | 0.21 | 0.22 | 0.13 | 0.09 | 0.08 |
| **NIL-U-2B-3** | Stress | 0.05 | 0.04 | 0.05 | 0.03 | 0.04 | 0.09 | 0.06 | 0.05 | 0.05 | 0.04 | 0.04 | 0.02 | 0.02 | 0.02 | 0.02 | 0.02 | 0.01 | 0.02 | 0.01 | 0.01 | 0.01 | 0.01 |
|  |  |  |  |  |  |  |  |  |  |  |  |  |  |  |  |  |  |  |  |  |  |  |  |
| **Combination** | **DAS** | **56** | **57** | **58** | **59** | **60** | **61** | **62** | **63** | **65** | **67** | **68** | **69** | **70** | **71** | **72** | **73** | **74** | **75** | **76** | **77** | **78** | **79** |
| **BarNir vs.** | Control | 0.00 | 0.00 | 0.00 | 0.00 | 0.00 | 0.00 | 0.00 | 0.00 | 0.00 | 0.00 | 0.00 | 0.00 | 0.00 | 0.00 | 0.00 | 0.00 | 0.00 | 0.00 | 0.00 | 0.00 | 0.00 | 0.00 |
| **NIL-B-7A-2** | Stress | 0.00 | 0.00 | 0.00 | 0.00 | 0.00 | 0.00 | 0.00 | 0.00 | 0.00 | 0.00 | 0.00 | 0.00 | 0.00 | 0.00 | 0.00 | 0.00 | 0.00 | 0.00 | 0.00 | 0.00 | 0.00 | 0.00 |
| **Zahir vs.** | Control | 0.55 | 0.59 | 0.41 | 0.48 | 0.08 | 0.15 | 0.07 | 0.07 | 0.05 | 0.03 | 0.05 | 0.07 | 0.09 | 0.05 | 0.06 | 0.08 | 0.06 | 0.05 | 0.08 | 0.22 | 0.08 | 0.04 |
| **NIL-Z-7A-5** | Stress | 0.35 | 0.50 | 0.77 | 0.53 | 0.28 | 0.47 | 0.39 | 0.33 | 0.23 | 0.32 | 0.23 | 0.59 | 0.45 | 0.90 | 0.72 | 0.75 | 0.91 | 0.96 | 0.91 | 0.69 | 0.90 | 0.80 |
| **Uzan vs.** | Control | 0.06 | 0.09 | 0.10 | 0.08 | 0.38 | 0.33 | 0.35 | 0.29 | 0.27 | 0.17 | 0.15 | 0.26 | 0.31 | 0.23 | 0.18 | 0.17 | 0.22 | 0.18 | 0.27 | 0.22 | 0.30 | 0.16 |
| **NIL-U-2B-3** | Stress | 0.01 | 0.00 | 0.00 | 0.00 | 0.00 | 0.01 | 0.00 | 0.00 | 0.00 | 0.00 | 0.00 | 0.00 | 0.00 | 0.00 | 0.00 | 0.00 | 0.00 | 0.00 | 0.00 | 0.00 | 0.00 | 0.01 |
|  |  |  |  |  |  |  |  |  |  |  |  |  |  |  |  |  |  |  |  |  |  |  |  |
| **Combination** | **DAS** | **80** | **81** | **82** | **83** | **85** | **86** | **88** | **90** | **92** | **94** | **96** | **98** | **100** | **102** | **104** | **106** | **108** | **110** | **112** | **114** | **116** | **121** |
| **BarNir vs.** | Control | 0.00 | 0.00 | 0.00 | 0.00 | 0.00 | 0.00 | 0.00 | 0.00 | 0.00 | 0.00 | 0.00 | 0.00 | 0.00 | 0.01 | 0.04 | 0.00 | 0.03 | 0.00 | 0.04 | 0.06 | 0.02 | 0.03 |
| **NIL-B-7A-2** | Stress | 0.00 | 0.00 | 0.00 | 0.00 | 0.00 | 0.00 | 0.00 | 0.00 | 0.00 | 0.00 | 0.00 | 0.00 | 0.00 | 0.00 |  |  |  |  |  |  |  |  |
| **Zahir vs.** | Control | 0.03 | 0.04 | 0.08 | 0.04 | 0.04 | 0.02 | 0.03 | 0.03 | 0.05 | 0.08 | 0.11 | 0.08 | 0.09 | 0.00 | 0.09 | 0.24 | 0.22 | 0.10 | 0.23 | 0.11 | 0.09 | 0.14 |
| **NIL-Z-7A-5** | Stress | 0.85 | 0.81 | 0.74 | 0.93 | 0.66 | 0.26 | 0.08 | 0.01 | 0.00 | 0.00 | 0.02 |  |  |  |  |  |  |  |  |  |  |  |
| **Uzan vs.** | Control | 0.15 | 0.17 | 0.24 | 0.19 | 0.25 | 0.23 | 0.29 | 0.45 | 0.67 | 0.80 | 0.93 | 0.89 | 0.70 | 0.90 | 0.78 | 0.88 | 0.96 | 0.88 | 0.59 | 0.87 | 0.93 | 0.88 |
| **NIL-U-2B-3** | Stress | 0.07 | 0.03 | 0.06 | 0.07 | 0.18 | 0.18 | 0.12 | 0.05 | 0.06 | 0.04 | 0.02 |  |  |  |  |  |  |  |  |  |  |  |

Significant differences calculated with a two-sided Student's t-test (p<0.05) are highlighted in green.

**Supplementary Material 9**: Descriptive statistics for the trait estimated biomass for selected days in the HTP experiment.

| **Genotype** | | **BarNir** | | **NIL-B-7A-2** | | **Zahir** | | **NIL-Z-7A-5** | | **Uzan** | | **NIL-U-2B-3** | |
| --- | --- | --- | --- | --- | --- | --- | --- | --- | --- | --- | --- | --- | --- |
| **Treatment** | | **Control** | **Stress** | **Control** | **Stress** | **Control** | **Stress** | **Control** | **Stress** | **Control** | **Stress** | **Control** | **Stress** |
| **DAS33** | **Av** | **0.15** | **0.13** | **0.16** | **0.17** | **0.13** | **0.08** | **0.09** | **0.08** | **0.13** | **0.11** | **0.09** | **0.15** |
|  | **SD** | 0.02 | 0.03 | 0.05 | 0.03 | 0.04 | 0.02 | 0.02 | 0.02 | 0.02 | 0.03 | 0.01 | 0.04 |
|  | **Min** | 0.10 | 0.10 | 0.07 | 0.12 | 0.06 | 0.06 | 0.05 | 0.05 | 0.10 | 0.06 | 0.07 | 0.08 |
|  | **Max** | 0.18 | 0.18 | 0.22 | 0.21 | 0.19 | 0.12 | 0.13 | 0.11 | 0.16 | 0.15 | 0.11 | 0.20 |
| **DAS40** | **Av** | **0.29** | **0.21** | **0.37** | **0.33** | **0.28** | **0.16** | **0.20** | **0.16** | **0.27** | **0.19** | **0.22** | **0.23** |
|  | **SD** | 0.05 | 0.04 | 0.11 | 0.05 | 0.08 | 0.03 | 0.03 | 0.03 | 0.05 | 0.04 | 0.03 | 0.04 |
|  | **Min** | 0.21 | 0.15 | 0.15 | 0.24 | 0.15 | 0.12 | 0.14 | 0.12 | 0.20 | 0.13 | 0.16 | 0.15 |
|  | **Max** | 0.35 | 0.27 | 0.50 | 0.41 | 0.43 | 0.22 | 0.24 | 0.20 | 0.37 | 0.25 | 0.27 | 0.29 |
| **DAS47** | **Av** | **0.39** | **0.20** | **0.63** | **0.39** | **0.44** | **0.22** | **0.38** | **0.21** | **0.43** | **0.27** | **0.43** | **0.33** |
|  | **SD** | 0.08 | 0.04 | 0.20 | 0.08 | 0.14 | 0.04 | 0.06 | 0.04 | 0.06 | 0.05 | 0.09 | 0.06 |
|  | **Min** | 0.27 | 0.15 | 0.33 | 0.22 | 0.23 | 0.16 | 0.26 | 0.15 | 0.36 | 0.18 | 0.24 | 0.19 |
|  | **Max** | 0.52 | 0.25 | 0.88 | 0.48 | 0.73 | 0.27 | 0.45 | 0.27 | 0.56 | 0.33 | 0.54 | 0.40 |
| **DAS54** | **Av** | **0.40** | **0.20** | **0.79** | **0.43** | **0.54** | **0.26** | **0.49** | **0.25** | **0.49** | **0.27** | **0.60** | **0.36** |
|  | **SD** | 0.08 | 0.04 | 0.28 | 0.06 | 0.19 | 0.04 | 0.09 | 0.04 | 0.05 | 0.05 | 0.18 | 0.07 |
|  | **Min** | 0.26 | 0.13 | 0.38 | 0.33 | 0.27 | 0.18 | 0.29 | 0.19 | 0.45 | 0.18 | 0.27 | 0.19 |
|  | **Max** | 0.53 | 0.27 | 1.18 | 0.52 | 0.93 | 0.34 | 0.59 | 0.32 | 0.60 | 0.35 | 0.84 | 0.46 |
| **DAS70** | **Av** | **0.38** | **0.15** | **0.91** | **0.32** | **0.50** | **0.20** | **0.38** | **0.19** | **0.48** | **0.18** | **0.55** | **0.24** |
|  | **SD** | 0.07 | 0.02 | 0.19 | 0.05 | 0.19 | 0.04 | 0.08 | 0.03 | 0.06 | 0.02 | 0.17 | 0.04 |
|  | **Min** | 0.30 | 0.12 | 0.52 | 0.21 | 0.18 | 0.12 | 0.22 | 0.13 | 0.39 | 0.13 | 0.26 | 0.15 |
|  | **Max** | 0.48 | 0.19 | 1.09 | 0.37 | 0.74 | 0.24 | 0.47 | 0.25 | 0.61 | 0.22 | 0.91 | 0.29 |
| **DAS80** | **Av** | **0.41** | **0.16** | **1.06** | **0.36** | **0.57** | **0.18** | **0.41** | **0.18** | **0.48** | **0.17** | **0.55** | **0.20** |
|  | **SD** | 0.07 | 0.02 | 0.19 | 0.06 | 0.18 | 0.03 | 0.08 | 0.02 | 0.06 | 0.03 | 0.13 | 0.03 |
|  | **Min** | 0.31 | 0.13 | 0.68 | 0.26 | 0.25 | 0.15 | 0.25 | 0.13 | 0.36 | 0.13 | 0.29 | 0.14 |
|  | **Max** | 0.53 | 0.19 | 1.30 | 0.44 | 0.89 | 0.23 | 0.51 | 0.22 | 0.61 | 0.23 | 0.73 | 0.25 |
| **DAS90** | **Av** | **0.45** | **0.13** | **0.91** | **0.29** | **0.59** | **0.14** | **0.43** | **0.20** | **0.46** | **0.10** | **0.49** | **0.12** |
|  | **SD** | 0.11 | 0.03 | 0.36 | 0.04 | 0.20 | 0.03 | 0.07 | 0.04 | 0.07 | 0.03 | 0.11 | 0.02 |
|  | **Min** | 0.29 | 0.08 | 0.39 | 0.25 | 0.24 | 0.09 | 0.26 | 0.10 | 0.30 | 0.05 | 0.27 | 0.09 |
|  | **Max** | 0.65 | 0.18 | 1.47 | 0.36 | 0.89 | 0.20 | 0.51 | 0.25 | 0.58 | 0.15 | 0.68 | 0.16 |

Av = Average, SD = Standard deviation, Min = Minimum, Max = Maximum

**Supplementary Material 10:** p-values of a comparison of control vs stress treatment for each genotype for Estimated Biovolume using t-test.

| **DAS** | **33** | **34** | **35** | **36** | **37** | **38** | **39** | **40** | **41** | **42** | **43** | **44** | **45** | **46** | **47** | **48** | **50** | **51** | **52** | **53** | **54** |
| --- | --- | --- | --- | --- | --- | --- | --- | --- | --- | --- | --- | --- | --- | --- | --- | --- | --- | --- | --- | --- | --- |
| **BarNir** | 0.29 | 0.14 | 0.09 | 0.05 | 0.05 | 0.01 | 0.00 | 0.00 | 0.00 | 0.00 | 0.00 | 0.00 | 0.00 | 0.00 | 0.00 | 0.00 | 0.00 | 0.00 | 0.00 | 0.00 | 0.00 |
| **NIL-B-7A-2** | 0.56 | 0.41 | 0.58 | 0.76 | 0.85 | 0.74 | 0.21 | 0.30 | 0.22 | 0.10 | 0.09 | 0.05 | 0.00 | 0.00 | 0.00 | 0.00 | 0.01 | 0.00 | 0.01 | 0.00 | 0.00 |
| **Zahir** | 0.00 | 0.00 | 0.00 | 0.00 | 0.00 | 0.00 | 0.00 | 0.00 | 0.00 | 0.00 | 0.00 | 0.00 | 0.00 | 0.00 | 0.00 | 0.00 | 0.00 | 0.00 | 0.00 | 0.00 | 0.00 |
| **NIL-Z-7A-5** | 0.48 | 0.35 | 0.36 | 0.32 | 0.15 | 0.07 | 0.03 | 0.00 | 0.00 | 0.00 | 0.00 | 0.00 | 0.00 | 0.00 | 0.00 | 0.00 | 0.00 | 0.00 | 0.00 | 0.00 | 0.00 |
| **Uzan** | 0.14 | 0.12 | 0.09 | 0.10 | 0.04 | 0.04 | 0.01 | 0.00 | 0.00 | 0.00 | 0.00 | 0.00 | 0.00 | 0.00 | 0.00 | 0.00 | 0.00 | 0.00 | 0.00 | 0.00 | 0.00 |
| **NIL-U-2B-3** | 0.00 | 0.00 | 0.01 | 0.01 | 0.01 | 0.02 | 0.08 | 0.36 | 0.90 | 0.72 | 0.25 | 0.09 | 0.05 | 0.02 | 0.01 | 0.00 | 0.01 | 0.00 | 0.00 | 0.00 | 0.00 |
|  |  |  |  |  |  |  |  |  |  |  |  |  |  |  |  |  |  |  |  |  |  |
| **DAS** | **55** | **56** | **57** | **58** | **59** | **60** | **61** | **62** | **63** | **65** | **67** | **68** | **69** | **70** | **71** | **72** | **73** | **74** | **75** | **76** | **77** |
| **BarNir** | 0.00 | 0.00 | 0.00 | 0.00 | 0.00 | 0.00 | 0.00 | 0.00 | 0.00 | 0.00 | 0.00 | 0.00 | 0.00 | 0.00 | 0.00 | 0.00 | 0.00 | 0.00 | 0.00 | 0.00 | 0.00 |
| **NIL-B-7A-2** | 0.00 | 0.00 | 0.00 | 0.00 | 0.00 | 0.00 | 0.00 | 0.00 | 0.00 | 0.00 | 0.00 | 0.00 | 0.00 | 0.00 | 0.00 | 0.00 | 0.00 | 0.00 | 0.00 | 0.00 | 0.00 |
| **Zahir** | 0.00 | 0.00 | 0.00 | 0.00 | 0.00 | 0.00 | 0.00 | 0.00 | 0.00 | 0.00 | 0.00 | 0.00 | 0.00 | 0.00 | 0.00 | 0.00 | 0.00 | 0.00 | 0.00 | 0.00 | 0.00 |
| **NIL-Z-7A-5** | 0.00 | 0.00 | 0.00 | 0.00 | 0.00 | 0.00 | 0.00 | 0.00 | 0.00 | 0.00 | 0.00 | 0.00 | 0.00 | 0.00 | 0.00 | 0.00 | 0.00 | 0.00 | 0.00 | 0.00 | 0.00 |
| **Uzan** | 0.00 | 0.00 | 0.00 | 0.00 | 0.00 | 0.00 | 0.00 | 0.00 | 0.00 | 0.00 | 0.00 | 0.00 | 0.00 | 0.00 | 0.00 | 0.00 | 0.00 | 0.00 | 0.00 | 0.00 | 0.00 |
| **NIL-U-2B-3** | 0.00 | 0.00 | 0.00 | 0.00 | 0.00 | 0.01 | 0.01 | 0.00 | 0.01 | 0.00 | 0.00 | 0.00 | 0.00 | 0.00 | 0.00 | 0.00 | 0.00 | 0.00 | 0.00 | 0.00 | 0.00 |
|  |  |  |  |  |  |  |  |  |  |  |  |  |  |  |  |  |  |  |  |  |  |
| **DAS** | **78** | **79** | **80** | **81** | **82** | **83** | **85** | **86** | **88** | **90** | **92** | **94** | **96** | **98** | **100** | **102** | **104** | **106** | **108** |  |  |
| **BarNir** | 0.00 | 0.00 | 0.00 | 0.00 | 0.00 | 0.00 | 0.00 | 0.00 | 0.00 | 0.00 | 0.00 | 0.00 | 0.00 | 0.00 | 0.00 | 0.00 |  |  |  |  |  |
| **NIL-B-7A-2** | 0.00 | 0.00 | 0.00 | 0.00 | 0.00 | 0.00 | 0.00 | 0.00 | 0.00 | 0.00 | 0.00 | 0.00 | 0.00 | 0.00 | 0.00 | 0.00 | 0.00 | 0.00 | 0.00 |  |  |
| **Zahir** | 0.00 | 0.00 | 0.00 | 0.00 | 0.00 | 0.00 | 0.00 | 0.00 | 0.00 | 0.00 | 0.00 | 0.00 | 0.00 |  |  |  |  |  |  |  |  |
| **NIL-Z-7A-5** | 0.00 | 0.00 | 0.00 | 0.00 | 0.00 | 0.00 | 0.00 | 0.00 | 0.00 | 0.00 | 0.00 | 0.00 | 0.00 | 0.00 | 0.00 | 0.00 |  |  |  |  |  |
| **Uzan** | 0.00 | 0.00 | 0.00 | 0.00 | 0.00 | 0.00 | 0.00 | 0.00 | 0.00 | 0.00 | 0.00 | 0.00 | 0.00 |  |  |  |  |  |  |  |  |
| **NIL-U-2B-3** | 0.00 | 0.00 | 0.00 | 0.00 | 0.00 | 0.00 | 0.00 | 0.00 | 0.00 | 0.00 | 0.00 | 0.00 | 0.00 | 0.00 | 0.00 | 0.00 |  |  |  |  |  |

Significant differences calculated with a two-sided Student's t-test (p<0.05) are highlighted in green.

**Supplementary Material 11:** p-values of a comparison of parent vs NIL during plant establishment phase using t-test for estimated biovolume, plant height and the color value

| **Estimated Biovolume**  **(voxel 10^6^)** |  |  |  |  |  |  |  |  |  |  |  |  |  |  |  |  |  |  | |
| --- | --- | --- | --- | --- | --- | --- | --- | --- | --- | --- | --- | --- | --- | --- | --- | --- | --- | --- | --- |
| **Combination** | **DAS** | **10** | **11** | **12** | **13** | **14** | **16** | **17** | **18** | **19** | **20** | **22** | **23** | **24** | **25** | **27** |  | |  |
| **BarNir vs. NIL-B-7A-2** | | 0.38 | 0.17 | 0.88 | 0.84 | 0.25 | 0.48 | 0.52 | 0.75 | 0.33 | 0.49 | 0.41 | 0.15 | 0.09 | 0.03 | 0.03 |  | |  |
| **Zahir vs. NIL-Z-7A-5** | | 0.00 | 0.00 | 0.00 | 0.04 | 0.07 | 0.10 | 0.06 | 0.58 | 0.46 | 0.12 | 0.64 | 0.45 | 0.25 | 0.27 | 0.40 |  | |  |
| **Uzan vs. NIL-U-2B-3** | | 0.92 | 0.21 | 0.10 | 0.04 | 0.03 | 0.01 | 0.03 | 0.17 | 0.13 | 0.24 | 0.10 | 0.08 | 0.12 | 0.09 | 0.09 |  | |  |
|  |  |  |  |  |  |  |  |  |  |  |  |  |  |  |  |  |  |  | |
| **Plant Height (mm)** |  |  |  |  |  |  |  |  |  |  |  |  |  |  |  |  |  |  | |
| **Combination** | **DAS** | **10** | **11** | **12** | **13** | **14** | **16** | **17** | **18** | **19** | **20** | **22** | **23** | **24** | **25** | **27** | **28** | **29** | |
| **BarNir vs. NIL-B-7A-2** | | 0.12 | 0.00 | 0.00 | 0.01 | 0.01 | 0.00 | 0.00 | 0.00 | 0.00 | 0.00 | 0.00 | 0.00 | 0.00 | 0.00 | 0.00 | 0.00 | 0.00 | |
| **Zahir vs. NIL-Z-7A-5** | | 0.72 | 0.17 | 0.60 | 0.18 | 0.21 | 0.29 | 0.10 | 0.35 | 0.45 | 0.99 | 0.67 | 0.64 | 0.35 | 0.04 | 0.18 | 0.26 | 0.20 | |
| **Uzan vs. NIL-U-2B-3** | | 0.37 | 0.03 | 0.06 | 0.08 | 0.03 | 0.90 | 0.78 | 0.23 | 0.17 | 0.15 | 0.36 | 0.73 | 0.23 | 0.17 | 0.56 | 0.66 | 0.35 | |
|  |  |  |  |  |  |  |  |  |  |  |  |  |  |  |  |  |  |  | |
| **Color Value (hue)** |  |  |  |  |  |  |  |  |  |  |  |  |  |  |  |  |  |  | |
| **Combination** | **DAS** | **10** | **11** | **12** | **13** | **14** | **16** | **17** | **18** | **19** | **20** | **22** | **23** | **24** | **25** | **27** | **28** | **29** | |
| **BarNir vs. NIL-B-7A-2** | | 0.00 | 0.00 | 0.00 | 0.00 | 0.00 | 0.00 | 0.02 | 0.01 | 0.00 | 0.00 | 0.00 | 0.00 | 0.00 | 0.00 | 0.00 | 0.00 | 0.00 | |
| **Zahir vs. NIL-Z-7A-5** | | 0.00 | 0.00 | 0.00 | 0.04 | 0.00 | 0.00 | 0.18 | 0.69 | 0.52 | 0.85 | 0.07 | 0.17 | 0.65 | 0.70 | 0.27 | 0.30 | 0.12 | |
| **Uzan vs. NIL-U-2B-3** | | 0.00 | 0.00 | 0.00 | 0.09 | 0.03 | 0.02 | 0.17 | 0.95 | 0.22 | 0.51 | 0.47 | 0.55 | 0.89 | 0.16 | 0.00 | 0.00 | 0.00 | |

Significant differences calculated with a two-sided Student's t-test (p<0.05) are highlighted in green.

**Supplementary Material 12:** p-values of a comparison of parent vs NIL for plant height using t-test.

| **Combination** | **DAS** | **30** | **31** | **32** | **33** | **34** | **35** | **36** | **37** | **38** | **39** | **40** | **41** | **42** | **43** | **44** | **45** | **46** | **47** | **48** | **50** | **51** | **52** | **53** |
| --- | --- | --- | --- | --- | --- | --- | --- | --- | --- | --- | --- | --- | --- | --- | --- | --- | --- | --- | --- | --- | --- | --- | --- | --- |
| **BarNir vs.** | Control | 0.02 | 0.02 | 0.01 | 0.04 | 0.01 | 0.00 | 0.00 | 0.00 | 0.00 | 0.00 | 0.00 | 0.00 | 0.00 | 0.00 | 0.00 | 0.00 | 0.00 | 0.00 | 0.00 | 0.00 | 0.00 | 0.00 | 0.00 |
| **NIL-B-7A-2** | Stress | 0.00 | 0.00 | 0.00 | 0.00 | 0.00 | 0.00 | 0.00 | 0.00 | 0.00 | 0.00 | 0.00 | 0.00 | 0.00 | 0.00 | 0.00 | 0.00 | 0.00 | 0.00 | 0.00 | 0.00 | 0.00 | 0.00 | 0.00 |
| **Zahir vs.** | Control | 0.81 | 0.81 | 0.91 | 0.87 | 0.50 | 0.91 | 0.91 | 0.59 | 0.50 | 0.30 | 0.32 | 0.63 | 0.66 | 0.24 | 0.09 | 0.07 | 0.45 | 0.63 | 0.64 | 0.67 | 0.52 | 0.39 | 0.55 |
| **NIL-Z-7A-5** | Stress | 0.15 | 0.15 | 0.01 | 0.06 | 0.59 | 0.45 | 0.92 | 0.88 | 0.75 | 0.76 | 0.79 | 0.61 | 0.51 | 0.69 | 0.77 | 0.61 | 0.47 | 0.45 | 0.39 | 0.34 | 0.33 | 0.02 | 0.00 |
| **Uzan vs.** | Control | 0.09 | 0.09 | 0.47 | 0.32 | 0.41 | 0.55 | 0.51 | 0.96 | 0.84 | 0.44 | 0.58 | 0.97 | 0.67 | 0.50 | 0.52 | 0.69 | 0.61 | 0.25 | 0.41 | 0.96 | 0.68 | 0.83 | 0.70 |
| **NIL-U-2B-3** | Stress | 0.56 | 0.56 | 0.61 | 0.73 | 0.54 | 0.45 | 0.40 | 0.27 | 0.15 | 0.05 | 0.03 | 0.03 | 0.06 | 0.10 | 0.56 | 0.89 | 0.46 | 0.58 | 0.47 | 0.70 | 0.07 | 0.20 | 0.05 |
|  |  |  |  |  |  |  |  |  |  |  |  |  |  |  |  |  |  |  |  |  |  |  |  |  |
| **Combination** | **DAS** | **54** | **55** | **56** | **57** | **58** | **59** | **60** | **61** | **62** | **63** | **65** | **67** | **68** | **69** | **70** | **71** | **72** | **73** | **74** | **75** | **76** | **77** | **78** |
| **BarNir vs.** | Control | 0.00 | 0.00 | 0.00 | 0.00 | 0.00 | 0.00 | 0.00 | 0.00 | 0.00 | 0.00 | 0.00 | 0.00 | 0.00 | 0.00 | 0.00 | 0.00 | 0.00 | 0.00 | 0.00 | 0.00 | 0.00 | 0.00 | 0.00 |
| **NIL-B-7A-2** | Stress | 0.00 | 0.00 | 0.00 | 0.00 | 0.00 | 0.00 | 0.00 | 0.00 | 0.00 | 0.00 | 0.00 | 0.00 | 0.00 | 0.00 | 0.00 | 0.00 | 0.00 | 0.00 | 0.00 | 0.00 | 0.00 | 0.00 | 0.00 |
| **Zahir vs.** | Control | 0.95 | 0.25 | 0.30 | 0.91 | 0.35 | 0.15 | 0.04 | 0.04 | 0.04 | 0.11 | 0.51 | 0.52 | 0.57 | 0.64 | 0.74 | 0.69 | 0.73 | 0.71 | 0.71 | 0.71 | 0.72 | 0.79 | 0.79 |
| **NIL-Z-7A-5** | Stress | 0.05 | 0.16 | 0.20 | 0.20 | 0.10 | 0.01 | 0.02 | 0.10 | 0.21 | 0.14 | 0.14 | 0.17 | 0.15 | 0.19 | 0.15 | 0.19 | 0.13 | 0.19 | 0.13 | 0.13 | 0.17 | 0.13 | 0.16 |
| **Uzan vs.** | Control | 0.32 | 0.18 | 0.06 | 0.06 | 0.03 | 0.04 | 0.03 | 0.03 | 0.03 | 0.04 | 0.09 | 0.01 | 0.01 | 0.01 | 0.02 | 0.02 | 0.02 | 0.02 | 0.02 | 0.01 | 0.01 | 0.01 | 0.01 |
| **NIL-U-2B-3** | Stress | 0.02 | 0.01 | 0.01 | 0.00 | 0.00 | 0.00 | 0.00 | 0.00 | 0.00 | 0.00 | 0.00 | 0.00 | 0.00 | 0.00 | 0.00 | 0.00 | 0.00 | 0.00 | 0.00 | 0.00 | 0.00 | 0.00 | 0.00 |
|  |  |  |  |  |  |  |  |  |  |  |  |  |  |  |  |  |  |  |  |  |  |  |  |  |
| **Combination** | **DAS** | **79** | **80** | **81** | **82** | **83** | **85** | **86** | **88** | **90** | **92** | **94** | **96** | **98** | **100** | **102** | **104** | **106** | **110** | **112** | **114** | **116** | **121** |  |
| **BarNir vs.** | Control | 0.00 | 0.00 | 0.00 | 0.00 | 0.00 | 0.00 | 0.00 | 0.00 | 0.00 | 0.00 | 0.00 | 0.00 | 0.00 | 0.00 | 0.00 | 0.00 | 0.00 | 0.00 | 0.00 | 0.00 | 0.00 | 0.00 |  |
| **NIL-B-7A-2** | Stress | 0.00 | 0.00 | 0.00 | 0.00 | 0.00 | 0.00 | 0.00 | 0.00 | 0.00 | 0.00 | 0.00 | 0.00 | 0.00 | 0.00 | 0.00 |  |  |  |  |  |  |  |  |
| **Zahir vs.** | Control | 0.86 | 0.88 | 0.92 | 0.96 | 0.94 | 0.90 | 0.84 | 0.76 | 0.72 | 0.84 | 0.36 | 0.05 | 0.03 | 0.07 | 0.08 | 0.41 | 0.52 | 0.53 | 0.57 | 0.56 | 0.50 | 0.66 |  |
| **NIL-Z-7A-5** | Stress | 0.75 | 0.79 | 0.34 | 0.62 | 0.56 | 0.56 | 0.90 | 0.89 | 0.41 | 0.76 | 0.31 | 0.31 |  |  |  |  |  |  |  |  |  |  |  |
| **Uzan vs.** | Control | 0.01 | 0.01 | 0.01 | 0.01 | 0.01 | 0.01 | 0.00 | 0.01 | 0.01 | 0.00 | 0.00 | 0.00 | 0.00 | 0.01 | 0.02 | 0.00 | 0.00 | 0.00 | 0.00 | 0.00 | 0.00 | 0.00 |  |
| **NIL-U-2B-3** | Stress | 0.00 | 0.00 | 0.00 | 0.00 | 0.00 | 0.00 | 0.00 | 0.00 | 0.00 | 0.00 | 0.00 | 0.00 |  |  |  |  |  |  |  |  |  |  |  |

Significant differences calculated with a two-sided Student's t-test (p<0.05) are highlighted in green.

**Supplementary Material 13:** Descriptive statistics for the trait plant height on selected days in the HTP experiment.

| **Genotype** | | **BarNir** | | **NIL-B-7A-2** | | **Zahir** | | **NIL-Z-7A-5** | | **Uzan** | | **NIL-U-2B-3** | |
| --- | --- | --- | --- | --- | --- | --- | --- | --- | --- | --- | --- | --- | --- |
| **Measurement** | | **Control** | **Stress** | **Control** | **Stress** | **Control** | **Stress** | **Control** | **Stress** | **Control** | **Stress** | **Control** | **Stress** |
| **DAS33** | **Av** | **396.3** | **374.1** | **447.9** | **487.9** | **469.3** | **468.4** | **467.0** | **444.8** | **469.5** | **464.5** | **457.9** | **468.7** |
|  | **SD** | 49.2 | 22.9 | 52.9 | 67.8 | 27.8 | 23.3 | 35.3 | 29.2 | 17.8 | 26.4 | 31.5 | 27.2 |
|  | **Min** | 317.6 | 338.8 | 378.6 | 385.3 | 413.6 | 431.9 | 414.2 | 382.6 | 443.5 | 425.2 | 431.5 | 419.3 |
|  | **Max** | 472.5 | 425.3 | 547.0 | 570.4 | 512.7 | 505.5 | 505.0 | 477.1 | 500.7 | 505.5 | 535.4 | 505.7 |
| **DAS40** | **Av** | **473.2** | **433.6** | **594.1** | **559.9** | **506.7** | **481.5** | **496.8** | **478.9** | **520.4** | **492.5** | **528.4** | **520.3** |
|  | **SD** | 31.4 | 26.8 | 40.0 | 44.2 | 22.4 | 20.5 | 21.0 | 22.2 | 18.1 | 20.7 | 40.4 | 32.1 |
|  | **Min** | 425.1 | 396.7 | 520.8 | 497.5 | 461.4 | 439.5 | 465.5 | 437.0 | 499.8 | 464.3 | 484.0 | 473.5 |
|  | **Max** | 508.6 | 474.1 | 657.1 | 611.6 | 543.1 | 507.7 | 528.8 | 510.2 | 546.7 | 528.2 | 628.8 | 572.5 |
| **DAS47** | **Av** | **474.8** | **443.6** | **662.9** | **609.2** | **596.0** | **545.3** | **585.8** | **524.0** | **625.5** | **550.1** | **605.6** | **540.7** |
|  | **SD** | 19.9 | 19.4 | 64.5 | 59.3 | 52.1 | 63.0 | 38.3 | 61.4 | 36.8 | 25.8 | 38.5 | 46.3 |
|  | **Min** | 439.2 | 403.9 | 568.2 | 522.2 | 519.0 | 462.7 | 507.0 | 452.6 | 562.3 | 501.2 | 547.1 | 452.1 |
|  | **Max** | 507.2 | 470.3 | 743.4 | 743.3 | 680.8 | 654.3 | 642.1 | 624.7 | 676.2 | 588.3 | 663.7 | 598.4 |
| **DAS54** | **Av** | **527.0** | **478.4** | **810.1** | **735.0** | **704.6** | **653.9** | **703.2** | **600.6** | **668.8** | **609.0** | **654.5** | **571.4** |
|  | **SD** | 17.1 | 14.4 | 46.7 | 28.4 | 39.9 | 42.2 | 56.0 | 53.6 | 32.1 | 31.4 | 30.7 | 35.0 |
|  | **Min** | 496.0 | 449.3 | 746.7 | 681.1 | 655.1 | 595.8 | 617.5 | 539.5 | 617.4 | 538.1 | 613.9 | 508.7 |
|  | **Max** | 556.1 | 501.3 | 897.0 | 782.2 | 770.1 | 709.4 | 785.2 | 690.5 | 710.7 | 652.2 | 701.0 | 619.8 |
| **DAS70** | **Av** | **526.3** | **473.4** | **797.1** | **714.7** | **808.3** | **749.1** | **813.6** | **727.5** | **674.5** | **616.3** | **631.3** | **533.0** |
|  | **SD** | 16.8 | 11.4 | 28.5 | 25.8 | 33.3 | 31.7 | 37.1 | 29.8 | 23.8 | 27.7 | 45.0 | 33.8 |
|  | **Min** | 491.1 | 452.9 | 756.6 | 665.2 | 765.1 | 708.2 | 752.3 | 667.3 | 638.5 | 547.3 | 572.5 | 476.9 |
|  | **Max** | 551.6 | 491.8 | 858.0 | 739.5 | 869.8 | 825.0 | 885.0 | 774.1 | 724.8 | 649.2 | 709.7 | 604.5 |
| **DAS80** | **Av** | **525.2** | **456.3** | **777.0** | **703.7** | **801.9** | **739.9** | **804.3** | **735.7** | **671.0** | **609.0** | **626.3** | **525.7** |
|  | **SD** | 16.7 | 10.5 | 26.7 | 18.8 | 33.0 | 33.4 | 40.9 | 32.8 | 23.8 | 32.6 | 41.1 | 34.0 |
|  | **Min** | 484.5 | 436.6 | 728.7 | 668.9 | 760.1 | 701.0 | 742.3 | 701.5 | 634.1 | 532.3 | 568.6 | 462.6 |
|  | **Max** | 547.4 | 471.9 | 817.6 | 726.8 | 861.3 | 821.1 | 878.7 | 801.0 | 722.0 | 655.6 | 690.0 | 601.2 |
| **DAS90** | **Av** | **496.7** | **439.2** | **739.8** | **685.5** | **788.5** | **697.9** | **794.6** | **711.6** | **661.2** | **597.3** | **607.4** | **515.2** |
|  | **SD** | 14.1 | 10.6 | 44.2 | 19.5 | 35.4 | 29.6 | 40.2 | 38.7 | 25.3 | 26.8 | 47.0 | 38.8 |
|  | **Min** | 470.3 | 420.2 | 655.8 | 658.9 | 745.5 | 659.5 | 737.1 | 671.8 | 627.9 | 532.2 | 538.4 | 439.1 |
|  | **Max** | 514.7 | 453.6 | 783.5 | 709.0 | 847.1 | 750.4 | 872.4 | 787.3 | 717.6 | 629.8 | 690.0 | 587.8 |

Av = Average, SD = Standard deviation, Min = Minimum, Max = Maximum.

**Supplementary Material 14:** p-values of a comparison of control vs stress treatment for plant height using t-test.

| **DAS** | **30** | **31** | **32** | **33** | **34** | **35** | **36** | **37** | **38** | **39** | **40** | **41** | **42** | **43** | **44** | **45** | **46** | **47** | **48** | **50** | **51** | **52** | **53** | **54** |
| --- | --- | --- | --- | --- | --- | --- | --- | --- | --- | --- | --- | --- | --- | --- | --- | --- | --- | --- | --- | --- | --- | --- | --- | --- |
| **BarNir** | 0.71 | 0.71 | 0.38 | 0.21 | 0.13 | 0.14 | 0.11 | 0.01 | 0.02 | 0.01 | 0.01 | 0.01 | 0.00 | 0.01 | 0.02 | 0.02 | 0.01 | 0.00 | 0.00 | 0.00 | 0.00 | 0.00 | 0.00 | 0.00 |
| **NIL-B-7A-2** | 0.08 | 0.08 | 0.02 | 0.16 | 0.03 | 0.59 | 0.98 | 0.97 | 0.34 | 0.14 | 0.09 | 0.15 | 0.11 | 0.04 | 0.00 | 0.01 | 0.03 | 0.07 | 0.14 | 0.21 | 0.01 | 0.00 | 0.00 | 0.00 |
| **Zahir** | 0.64 | 0.64 | 0.92 | 0.94 | 0.05 | 0.37 | 0.04 | 0.15 | 0.06 | 0.02 | 0.02 | 0.00 | 0.00 | 0.00 | 0.01 | 0.02 | 0.06 | 0.07 | 0.05 | 0.10 | 0.01 | 0.16 | 0.06 | 0.02 |
| **NIL-Z-7A-5** | 0.86 | 0.86 | 0.04 | 0.14 | 0.25 | 0.21 | 0.20 | 0.39 | 0.21 | 0.16 | 0.08 | 0.16 | 0.32 | 0.27 | 0.10 | 0.16 | 0.05 | 0.01 | 0.01 | 0.00 | 0.00 | 0.00 | 0.00 | 0.00 |
| **Uzan** | 0.19 | 0.19 | 0.72 | 0.63 | 0.69 | 0.65 | 0.50 | 0.21 | 0.06 | 0.08 | 0.00 | 0.00 | 0.00 | 0.00 | 0.00 | 0.00 | 0.00 | 0.00 | 0.00 | 0.07 | 0.03 | 0.02 | 0.00 | 0.00 |
| **NIL-U-2B-3** | 0.56 | 0.56 | 0.40 | 0.42 | 0.34 | 0.41 | 0.43 | 0.92 | 0.91 | 0.85 | 0.63 | 0.41 | 0.20 | 0.07 | 0.01 | 0.00 | 0.00 | 0.00 | 0.00 | 0.01 | 0.00 | 0.00 | 0.00 | 0.00 |
|  |  |  |  |  |  |  |  |  |  |  |  |  |  |  |  |  |  |  |  |  |  |  |  |  |
| **DAS** | **55** | **56** | **57** | **58** | **59** | **60** | **61** | **62** | **63** | **65** | **67** | **68** | **69** | **70** | **71** | **72** | **73** | **74** | **75** | **76** | **77** | **78** | **79** | **80** |
| **BarNir** | 0.00 | 0.00 | 0.00 | 0.00 | 0.00 | 0.00 | 0.00 | 0.00 | 0.00 | 0.00 | 0.00 | 0.00 | 0.00 | 0.00 | 0.00 | 0.00 | 0.00 | 0.00 | 0.00 | 0.00 | 0.00 | 0.00 | 0.00 | 0.00 |
| **NIL-B-7A-2** | 0.00 | 0.00 | 0.00 | 0.00 | 0.00 | 0.00 | 0.00 | 0.00 | 0.00 | 0.00 | 0.00 | 0.00 | 0.00 | 0.00 | 0.00 | 0.00 | 0.00 | 0.00 | 0.00 | 0.00 | 0.00 | 0.00 | 0.00 | 0.00 |
| **Zahir** | 0.10 | 0.12 | 0.20 | 0.52 | 0.84 | 0.63 | 0.12 | 0.00 | 0.00 | 0.00 | 0.00 | 0.00 | 0.00 | 0.00 | 0.00 | 0.00 | 0.00 | 0.00 | 0.00 | 0.00 | 0.00 | 0.00 | 0.00 | 0.00 |
| **NIL-Z-7A-5** | 0.09 | 0.08 | 0.04 | 0.01 | 0.00 | 0.00 | 0.00 | 0.00 | 0.00 | 0.00 | 0.00 | 0.00 | 0.00 | 0.00 | 0.00 | 0.00 | 0.00 | 0.00 | 0.00 | 0.00 | 0.00 | 0.00 | 0.00 | 0.00 |
| **Uzan** | 0.00 | 0.00 | 0.00 | 0.00 | 0.00 | 0.00 | 0.00 | 0.00 | 0.00 | 0.00 | 0.00 | 0.00 | 0.00 | 0.00 | 0.00 | 0.00 | 0.00 | 0.00 | 0.00 | 0.00 | 0.00 | 0.00 | 0.00 | 0.00 |
| **NIL-U-2B-3** | 0.00 | 0.00 | 0.00 | 0.00 | 0.00 | 0.00 | 0.00 | 0.00 | 0.00 | 0.00 | 0.00 | 0.00 | 0.00 | 0.00 | 0.00 | 0.00 | 0.00 | 0.00 | 0.00 | 0.00 | 0.00 | 0.00 | 0.00 | 0.00 |
|  |  |  |  |  |  |  |  |  |  |  |  |  |  |  |  |  |  |  |  |  |  |  |  |  |
| **DAS** | **81** | **82** | **83** | **85** | **86** | **88** | **90** | **92** | **94** | **96** | **98** | **100** | **102** | **104** | **106** |  |  |  |  |  |  |  |  |  |
| **BarNir** | 0.00 | 0.00 | 0.00 | 0.00 | 0.00 | 0.00 | 0.00 | 0.00 | 0.00 | 0.00 | 0.00 | 0.00 | 0.00 |  |  |  |  |  |  |  |  |  |  |  |
| **NIL-B-7A-2** | 0.00 | 0.00 | 0.00 | 0.00 | 0.00 | 0.00 | 0.00 | 0.01 | 0.01 | 0.01 | 0.01 | 0.01 | 0.13 | 0.03 | 0.02 |  |  |  |  |  |  |  |  |  |
| **Zahir** | 0.00 | 0.00 | 0.00 | 0.00 | 0.00 | 0.00 | 0.00 | 0.00 | 0.00 | 0.00 |  |  |  |  |  |  |  |  |  |  |  |  |  |  |
| **NIL-Z-7A-5** | 0.00 | 0.00 | 0.00 | 0.00 | 0.00 | 0.00 | 0.00 | 0.00 | 0.00 | 0.00 | 0.00 | 0.00 | 0.01 |  |  |  |  |  |  |  |  |  |  |  |
| **Uzan** | 0.00 | 0.00 | 0.00 | 0.00 | 0.00 | 0.00 | 0.00 | 0.00 | 0.00 | 0.00 |  |  |  |  |  |  |  |  |  |  |  |  |  |  |
| **NIL-U-2B-3** | 0.00 | 0.00 | 0.00 | 0.00 | 0.00 | 0.00 | 0.00 | 0.00 | 0.00 | 0.00 | 0.00 | 0.00 | 0.00 |  |  |  |  |  |  |  |  |  |  |  |

Significant differences calculated with a two-sided Student's t-test (p<0.05) are highlighted in green.

**Supplementary Material 15:** p-values of a comparison of parent vs NIL for color value using t-test.

| **Combination** | **DAS** | **30** | **31** | **32** | **33** | **34** | **35** | **36** | **37** | **38** | **39** | **40** | **41** | **42** | **43** | **44** | **45** | **46** | **47** | **48** | **50** | **51** | **52** | **53** |
| --- | --- | --- | --- | --- | --- | --- | --- | --- | --- | --- | --- | --- | --- | --- | --- | --- | --- | --- | --- | --- | --- | --- | --- | --- |
| **BarNir vs.** | Control | 0.00 | 0.00 | 0.00 | 0.01 | 0.00 | 0.00 | 0.00 | 0.00 | 0.00 | 0.00 | 0.00 | 0.00 | 0.00 | 0.00 | 0.00 | 0.00 | 0.00 | 0.00 | 0.00 | 0.01 | 0.00 | 0.00 | 0.00 |
| **NIL-B-7A-2** | Stress | 0.00 | 0.00 | 0.00 | 0.00 | 0.00 | 0.00 | 0.00 | 0.00 | 0.00 | 0.00 | 0.00 | 0.00 | 0.00 | 0.00 | 0.00 | 0.00 | 0.00 | 0.00 | 0.00 | 0.02 | 0.00 | 0.04 | 0.02 |
| **Zahir vs.** | Control | 0.11 | 0.87 | 0.34 | 0.43 | 0.71 | 0.97 | 0.70 | 0.11 | 0.03 | 0.23 | 0.72 | 1.00 | 0.85 | 0.30 | 0.59 | 0.16 | 0.17 | 0.53 | 0.84 | 0.82 | 0.18 | 0.46 | 0.31 |
| **NIL-Z-7A-5** | Stress | 0.74 | 0.76 | 0.99 | 0.23 | 0.68 | 0.13 | 0.13 | 0.10 | 0.03 | 0.28 | 0.11 | 0.83 | 0.34 | 0.23 | 0.28 | 0.30 | 0.04 | 0.28 | 0.25 | 0.65 | 0.02 | 0.55 | 0.50 |
| **Uzan vs.** | Control | 0.01 | 0.01 | 0.02 | 0.39 | 0.01 | 0.00 | 0.01 | 0.02 | 0.01 | 0.01 | 0.01 | 0.05 | 0.00 | 0.05 | 0.01 | 0.00 | 0.00 | 0.00 | 0.01 | 0.09 | 0.00 | 0.01 | 0.00 |
| **NIL-U-2B-3** | Stress | 0.00 | 0.00 | 0.00 | 0.21 | 0.05 | 0.01 | 0.02 | 0.02 | 0.00 | 0.00 | 0.00 | 0.00 | 0.00 | 0.00 | 0.00 | 0.00 | 0.00 | 0.00 | 0.00 | 0.00 | 0.00 | 0.00 | 0.00 |
|  |  |  |  |  |  |  |  |  |  |  |  |  |  |  |  |  |  |  |  |  |  |  |  |  |
| **Combination** | **DAS** | **54** | **55** | **56** | **57** | **58** | **59** | **60** | **61** | **62** | **63** | **65** | **67** | **68** | **69** | **70** | **71** | **72** | **73** | **74** | **75** | **76** | **77** | **78** |
| **BarNir vs.** | Control | 0.00 | 0.00 | 0.00 | 0.01 | 0.02 | 0.02 | 0.02 | 0.91 | 0.00 | 0.00 | 0.02 | 0.57 | 0.25 | 0.45 | 0.93 | 0.16 | 0.00 | 0.18 | 0.95 | 0.24 | 0.03 | 0.04 | 0.05 |
| **NIL-B-7A-2** | Stress | 0.00 | 0.01 | 0.00 | 0.01 | 0.00 | 0.00 | 0.00 | 0.37 | 0.09 | 0.10 | 0.02 | 0.58 | 0.02 | 0.80 | 0.34 | 0.47 | 0.73 | 0.64 | 0.53 | 0.45 | 0.11 | 0.26 | 0.51 |
| **Zahir vs.** | Control | 0.71 | 0.37 | 0.90 | 0.34 | 0.73 | 0.82 | 0.37 | 0.85 | 0.30 | 0.20 | 0.36 | 0.20 | 0.47 | 0.83 | 0.19 | 0.87 | 0.02 | 0.01 | 0.57 | 0.75 | 0.07 | 0.39 | 0.34 |
| **NIL-Z-7A-5** | Stress | 0.03 | 0.56 | 0.14 | 0.02 | 0.06 | 0.00 | 0.01 | 0.36 | 0.50 | 0.09 | 0.08 | 0.17 | 0.05 | 0.18 | 0.16 | 0.38 | 0.01 | 0.02 | 0.10 | 0.06 | 0.00 | 0.12 | 0.13 |
| **Uzan vs.** | Control | 0.00 | 0.00 | 0.00 | 0.00 | 0.00 | 0.00 | 0.00 | 0.01 | 0.00 | 0.00 | 0.00 | 0.05 | 0.00 | 0.03 | 0.74 | 0.37 | 0.00 | 0.00 | 0.07 | 0.02 | 0.00 | 0.13 | 0.88 |
| **NIL-U-2B-3** | Stress | 0.00 | 0.00 | 0.00 | 0.00 | 0.00 | 0.00 | 0.00 | 1.00 | 0.00 | 0.00 | 0.00 | 0.06 | 0.01 | 0.10 | 0.01 | 0.02 | 0.18 | 0.19 | 0.01 | 0.01 | 0.04 | 0.27 | 0.65 |
|  |  |  |  |  |  |  |  |  |  |  |  |  |  |  |  |  |  |  |  |  |  |  |  |  |
| **Combination** | **DAS** | **79** | **80** | **81** | **82** | **83** | **85** | **86** | **88** | **90** | **92** | **94** | **96** | **98** | **100** | **102** | **104** | **106** | **108** | **110** | **112** | **114** | **116** | **121** |
| **BarNir vs.** | Control | 0.89 | 0.22 | 0.27 | 0.35 | 0.85 | 0.01 | 0.57 | 0.40 | 0.30 | 0.25 | 0.43 | 0.60 | 0.92 | 0.98 | 0.84 | 0.83 | 0.34 | 0.62 | 0.53 | 0.64 | 0.53 | 0.50 | 0.32 |
| **NIL-B-7A-2** | Stress | 0.21 | 0.38 | 0.73 | 0.73 | 0.73 | 0.56 | 0.19 | 0.07 | 0.02 | 0.00 | 0.00 | 0.00 | 0.01 | 0.00 |  |  |  |  |  |  |  |  |  |
| **Zahir vs.** | Control | 0.96 | 0.25 | 0.13 | 0.08 | 0.34 | 0.46 | 0.32 | 0.19 | 0.48 | 0.17 | 0.14 | 0.06 | 0.01 | 0.01 | 0.00 | 0.00 | 0.00 | 0.00 | 0.00 | 0.00 | 0.00 | 0.00 | 0.02 |
| **NIL-Z-7A-5** | Stress | 0.01 | 0.07 | 0.12 | 0.05 | 0.03 | 0.02 | 0.02 | 0.01 | 0.04 | 0.04 | 0.08 | 0.16 |  |  |  |  |  |  |  |  |  |  |  |
| **Uzan vs.** | Control | 0.01 | 0.00 | 0.02 | 0.99 | 0.00 | 0.00 | 0.01 | 0.03 | 0.04 | 0.19 | 0.19 | 0.24 | 0.26 | 0.14 | 0.10 | 0.12 | 0.09 | 0.18 | 0.08 | 0.12 | 0.12 | 0.13 | 0.23 |
| **NIL-U-2B-3** | Stress | 0.21 | 0.07 | 0.25 | 0.40 | 0.25 | 0.31 | 0.43 | 0.62 | 0.48 | 0.43 | 0.53 | 0.45 |  |  |  |  |  |  |  |  |  |  |  |

Significant differences calculated with a two-sided Student's t-test (p<0.05) are highlighted in green. **Supplementary Material 16:** Descriptive statistics for the trait color value for selected days in the HTP experiment.

| **Genotype** | | **BarNir** | | **NIL-B-7A-2** | | **Zahir** | | **NIL-Z-7A-5** | | **Uzan** | | **NIL-U-2B-3** | |
| --- | --- | --- | --- | --- | --- | --- | --- | --- | --- | --- | --- | --- | --- |
| **Measurement** | | **Control** | **Stress** | **Control** | **Stress** | **Control** | **Stress** | **Control** | **Stress** | **Control** | **Stress** | **Control** | **Stress** |
| **DAS33** | **Av** | **0.234** | **0.235** | **0.229** | **0.229** | **0.231** | **0.228** | **0.232** | **0.230** | **0.228** | **0.229** | **0.227** | **0.227** |
|  | **SD** | 0.004 | 0.002 | 0.003 | 0.003 | 0.003 | 0.003 | 0.002 | 0.003 | 0.003 | 0.003 | 0.003 | 0.004 |
|  | **Min** | 0.228 | 0.232 | 0.225 | 0.223 | 0.226 | 0.221 | 0.228 | 0.225 | 0.225 | 0.225 | 0.220 | 0.219 |
|  | **Max** | 0.238 | 0.238 | 0.232 | 0.232 | 0.235 | 0.232 | 0.234 | 0.233 | 0.235 | 0.234 | 0.231 | 0.231 |
| **DAS40** | **Av** | **0.235** | **0.236** | **0.230** | **0.230** | **0.232** | **0.231** | **0.232** | **0.232** | **0.231** | **0.232** | **0.228** | **0.228** |
|  | **SD** | 0.002 | 0.001 | 0.002 | 0.002 | 0.001 | 0.001 | 0.001 | 0.001 | 0.001 | 0.001 | 0.002 | 0.002 |
|  | **Min** | 0.233 | 0.233 | 0.228 | 0.228 | 0.230 | 0.229 | 0.230 | 0.230 | 0.227 | 0.230 | 0.225 | 0.226 |
|  | **Max** | 0.238 | 0.238 | 0.232 | 0.232 | 0.235 | 0.233 | 0.235 | 0.234 | 0.233 | 0.234 | 0.231 | 0.231 |
| **DAS47** | **Av** | **0.234** | **0.232** | **0.229** | **0.229** | **0.232** | **0.231** | **0.232** | **0.232** | **0.230** | **0.231** | **0.227** | **0.228** |
|  | **SD** | 0.002 | 0.001 | 0.001 | 0.002 | 0.002 | 0.003 | 0.002 | 0.001 | 0.001 | 0.002 | 0.002 | 0.003 |
|  | **Min** | 0.229 | 0.230 | 0.227 | 0.225 | 0.229 | 0.226 | 0.230 | 0.229 | 0.227 | 0.228 | 0.223 | 0.225 |
|  | **Max** | 0.237 | 0.234 | 0.231 | 0.231 | 0.234 | 0.235 | 0.234 | 0.234 | 0.232 | 0.234 | 0.230 | 0.232 |
| **DAS54** | **Av** | **0.228** | **0.229** | **0.224** | **0.225** | **0.229** | **0.227** | **0.229** | **0.229** | **0.227** | **0.229** | **0.223** | **0.223** |
|  | **SD** | 0.003 | 0.003 | 0.002 | 0.002 | 0.002 | 0.003 | 0.003 | 0.001 | 0.001 | 0.001 | 0.002 | 0.002 |
|  | **Min** | 0.224 | 0.224 | 0.221 | 0.221 | 0.225 | 0.224 | 0.225 | 0.227 | 0.225 | 0.227 | 0.219 | 0.219 |
|  | **Max** | 0.233 | 0.233 | 0.227 | 0.228 | 0.233 | 0.232 | 0.234 | 0.231 | 0.230 | 0.231 | 0.226 | 0.226 |
| **DAS70** | **Av** | **0.215** | **0.209** | **0.215** | **0.204** | **0.216** | **0.205** | **0.212** | **0.209** | **0.212** | **0.205** | **0.212** | **0.193** |
|  | **SD** | 0.007 | 0.014 | 0.004 | 0.008 | 0.004 | 0.008 | 0.008 | 0.005 | 0.005 | 0.010 | 0.004 | 0.008 |
|  | **Min** | 0.204 | 0.184 | 0.205 | 0.187 | 0.209 | 0.191 | 0.203 | 0.202 | 0.205 | 0.191 | 0.203 | 0.179 |
|  | **Max** | 0.222 | 0.221 | 0.220 | 0.213 | 0.222 | 0.216 | 0.222 | 0.217 | 0.219 | 0.216 | 0.217 | 0.204 |
| **DAS80** | **Av** | **0.204** | **0.166** | **0.198** | **0.174** | **0.198** | **0.178** | **0.205** | **0.187** | **0.209** | **0.168** | **0.191** | **0.156** |
|  | **SD** | 0.014 | 0.016 | 0.008 | 0.022 | 0.010 | 0.012 | 0.013 | 0.009 | 0.010 | 0.013 | 0.006 | 0.015 |
|  | **Min** | 0.184 | 0.132 | 0.190 | 0.136 | 0.186 | 0.154 | 0.183 | 0.175 | 0.192 | 0.146 | 0.184 | 0.132 |
|  | **Max** | 0.218 | 0.185 | 0.214 | 0.207 | 0.215 | 0.191 | 0.220 | 0.200 | 0.217 | 0.191 | 0.206 | 0.176 |
| **DAS90** | **Av** | **0.205** | **0.114** | **0.201** | **0.146** | **0.207** | **0.115** | **0.208** | **0.146** | **0.203** | **0.092** | **0.193** | **0.087** |
|  | **SD** | 0.006 | 0.025 | 0.007 | 0.028 | 0.002 | 0.029 | 0.003 | 0.032 | 0.009 | 0.020 | 0.009 | 0.013 |
|  | **Min** | 0.188 | 0.076 | 0.189 | 0.115 | 0.203 | 0.074 | 0.202 | 0.084 | 0.189 | 0.069 | 0.177 | 0.069 |
|  | **Max** | 0.214 | 0.154 | 0.210 | 0.179 | 0.210 | 0.175 | 0.211 | 0.176 | 0.212 | 0.130 | 0.203 | 0.113 |

Av = Average, SD = Standard deviation, Min = Minimum, Max = Maximum.

**Supplementary Material 17:** p-values of a comparison of control vs stress treatment for color value using t-test**.**

| **DAS** | **30** | **31** | **32** | **33** | **34** | **35** | **36** | **37** | **38** | **39** | **40** | **41** | **42** | **43** | **44** | **45** | **46** | **47** | **48** | **50** | **51** | **52** | **53** | **54** | **55** | **56** |
| --- | --- | --- | --- | --- | --- | --- | --- | --- | --- | --- | --- | --- | --- | --- | --- | --- | --- | --- | --- | --- | --- | --- | --- | --- | --- | --- |
| **BarNir** | 0.62 | 0.69 | 0.68 | 0.35 | 0.08 | 0.13 | 0.91 | 0.07 | 0.96 | 0.39 | 0.28 | 0.38 | 0.35 | 0.50 | 0.93 | 0.78 | 0.55 | 0.08 | 0.19 | 0.57 | 0.38 | 0.99 | 0.94 | 0.70 | 0.92 | 0.94 |
| **NIL-B-7A-2** | 0.65 | 0.68 | 0.79 | 0.99 | 0.40 | 0.70 | 0.91 | 0.55 | 0.59 | 0.46 | 0.92 | 0.99 | 0.98 | 0.73 | 0.52 | 0.75 | 0.42 | 0.71 | 0.87 | 0.59 | 0.33 | 0.31 | 0.47 | 0.57 | 0.02 | 0.29 |
| **Zahir** | 0.14 | 0.51 | 0.67 | 0.05 | 0.79 | 0.14 | 0.05 | 0.34 | 0.98 | 0.80 | 0.12 | 0.70 | 0.14 | 0.04 | 0.03 | 0.22 | 0.32 | 0.63 | 0.42 | 0.21 | 0.02 | 0.64 | 0.30 | 0.05 | 0.56 | 0.09 |
| **NIL-Z-7A-5** | 0.91 | 0.80 | 0.25 | 0.06 | 0.83 | 0.84 | 0.85 | 0.60 | 0.68 | 0.81 | 0.59 | 0.80 | 0.31 | 0.04 | 0.16 | 0.13 | 0.55 | 0.95 | 0.94 | 0.67 | 0.23 | 0.63 | 0.12 | 0.57 | 0.35 | 0.75 |
| **Uzan** | 0.81 | 0.89 | 0.66 | 0.82 | 0.92 | 0.52 | 0.12 | 0.17 | 0.20 | 0.11 | 0.06 | 0.10 | 0.00 | 0.01 | 0.04 | 0.21 | 0.40 | 0.20 | 0.19 | 0.87 | 0.86 | 0.14 | 0.05 | 0.00 | 0.61 | 0.51 |
| **NIL-U-2B-3** | 0.04 | 0.51 | 0.18 | 0.74 | 0.84 | 0.17 | 0.27 | 0.75 | 0.43 | 0.50 | 0.45 | 0.97 | 0.42 | 0.65 | 0.38 | 0.35 | 0.65 | 0.85 | 0.61 | 0.16 | 0.41 | 0.77 | 0.77 | 0.82 | 0.74 | 0.29 |
|  |  |  |  |  |  |  |  |  |  |  |  |  |  |  |  |  |  |  |  |  |  |  |  |  |  |  |
| **DAS** | **57** | **58** | **59** | **60** | **61** | **62** | **63** | **65** | **67** | **68** | **69** | **70** | **71** | **72** | **73** | **74** | **75** | **76** | **77** | **78** | **79** | **80** | **81** | **82** | **83** | **85** |
| **BarNir** | 0.68 | 0.62 | 0.54 | 0.14 | 0.33 | 0.27 | 0.75 | 0.71 | 0.07 | 0.43 | 0.06 | 0.25 | 0.04 | 0.00 | 0.01 | 0.01 | 0.00 | 0.00 | 0.00 | 0.00 | 0.00 | 0.00 | 0.00 | 0.00 | 0.00 | 0.00 |
| **NIL-B-7A-2** | 0.70 | 0.40 | 0.82 | 0.19 | 0.95 | 0.91 | 0.30 | 0.52 | 0.11 | 0.22 | 0.07 | 0.00 | 0.01 | 0.00 | 0.01 | 0.00 | 0.00 | 0.00 | 0.00 | 0.00 | 0.00 | 0.00 | 0.00 | 0.00 | 0.00 | 0.00 |
| **Zahir** | 0.01 | 0.28 | 0.09 | 0.54 | 0.06 | 0.08 | 0.06 | 0.53 | 0.36 | 0.10 | 0.00 | 0.00 | 0.02 | 0.00 | 0.00 | 0.00 | 0.00 | 0.00 | 0.00 | 0.00 | 0.00 | 0.00 | 0.00 | 0.00 | 0.00 | 0.00 |
| **NIL-Z-7A-5** | 0.78 | 0.86 | 0.22 | 0.69 | 0.61 | 0.88 | 0.12 | 0.81 | 0.78 | 0.83 | 0.02 | 0.32 | 0.20 | 0.00 | 0.00 | 0.00 | 0.00 | 0.00 | 0.00 | 0.00 | 0.00 | 0.00 | 0.00 | 0.00 | 0.00 | 0.00 |
| **Uzan** | 0.58 | 0.70 | 0.76 | 0.49 | 0.01 | 0.38 | 0.65 | 0.06 | 0.17 | 0.03 | 0.04 | 0.07 | 0.16 | 0.00 | 0.00 | 0.00 | 0.00 | 0.00 | 0.00 | 0.00 | 0.00 | 0.00 | 0.00 | 0.00 | 0.00 | 0.00 |
| **NIL-U-2B-3** | 0.16 | 0.36 | 0.34 | 0.77 | 0.83 | 0.00 | 0.51 | 0.00 | 0.06 | 0.00 | 0.01 | 0.00 | 0.00 | 0.00 | 0.00 | 0.00 | 0.00 | 0.00 | 0.00 | 0.00 | 0.00 | 0.00 | 0.00 | 0.00 | 0.00 | 0.00 |
|  |  |  |  |  |  |  |  |  |  |  |  |  |  |  |  |  |  |  |  |  |  |  |  |  |  |  |
| **DAS** | **86** | **88** | **90** | **92** | **94** | **96** | **98** | **100** | **102** | **104** | **106** | **108** |  |  |  |  |  |  |  |  |  |  |  |  |  |  |
| **BarNir** | 0.00 | 0.00 | 0.00 | 0.00 | 0.00 | 0.00 | 0.00 | 0.00 | 0.00 |  |  |  |  |  |  |  |  |  |  |  |  |  |  |  |  |  |
| **NIL-B-7A-2** | 0.00 | 0.00 | 0.00 | 0.00 | 0.00 | 0.02 | 0.02 | 0.01 |  | 0.13 | 0.21 | 0.44 |  |  |  |  |  |  |  |  |  |  |  |  |  |  |
| **Zahir** | 0.00 | 0.00 | 0.00 | 0.00 | 0.00 | 0.00 |  |  |  |  |  |  |  |  |  |  |  |  |  |  |  |  |  |  |  |  |
| **NIL-Z-7A-5** | 0.00 | 0.00 | 0.00 | 0.00 | 0.00 | 0.00 | 0.00 | 0.00 | 0.00 |  |  |  |  |  |  |  |  |  |  |  |  |  |  |  |  |  |
| **Uzan** | 0.00 | 0.00 | 0.00 | 0.00 | 0.00 | 0.00 |  |  |  |  |  |  |  |  |  |  |  |  |  |  |  |  |  |  |  |  |
| **NIL-U-2B-3** | 0.00 | 0.00 | 0.00 | 0.00 | 0.00 | 0.00 | 0.00 | 0.00 | 0.00 |  |  |  |  |  |  |  |  |  |  |  |  |  |  |  |  |  |

Significant differences calculated with a two-sided Student's t-test (p<0.05) are highlighted in green.

**Supplementary Material 18**: Descriptive statistics for the trait color value for selected days in the HTP experiment.

| **Genotype** | | **BarNir** | | **NIL-B-7A-2** | | **Zahir** | | **NIL-Z-7A-5** | | **Uzan** | | **NIL-U-2B-3** | |
| --- | --- | --- | --- | --- | --- | --- | --- | --- | --- | --- | --- | --- | --- |
| **Measurement** | | **Control** | **Stress** | **Control** | **Stress** | **Control** | **Stress** | **Control** | **Stress** | **Control** | **Stress** | **Control** | **Stress** |
| **DAS33** | **Av** | **0.234** | **0.235** | **0.229** | **0.229** | **0.231** | **0.228** | **0.232** | **0.230** | **0.228** | **0.229** | **0.227** | **0.227** |
|  | **SD** | 0.004 | 0.002 | 0.003 | 0.003 | 0.003 | 0.003 | 0.002 | 0.003 | 0.003 | 0.003 | 0.003 | 0.004 |
|  | **Min** | 0.228 | 0.232 | 0.225 | 0.223 | 0.226 | 0.221 | 0.228 | 0.225 | 0.225 | 0.225 | 0.220 | 0.219 |
|  | **Max** | 0.238 | 0.238 | 0.232 | 0.232 | 0.235 | 0.232 | 0.234 | 0.233 | 0.235 | 0.234 | 0.231 | 0.231 |
| **DAS40** | **Av** | **0.235** | **0.236** | **0.230** | **0.230** | **0.232** | **0.231** | **0.232** | **0.232** | **0.231** | **0.232** | **0.228** | **0.228** |
|  | **SD** | 0.002 | 0.001 | 0.002 | 0.002 | 0.001 | 0.001 | 0.001 | 0.001 | 0.001 | 0.001 | 0.002 | 0.002 |
|  | **Min** | 0.233 | 0.233 | 0.228 | 0.228 | 0.230 | 0.229 | 0.230 | 0.230 | 0.227 | 0.230 | 0.225 | 0.226 |
|  | **Max** | 0.238 | 0.238 | 0.232 | 0.232 | 0.235 | 0.233 | 0.235 | 0.234 | 0.233 | 0.234 | 0.231 | 0.231 |
| **DAS47** | **Av** | **0.234** | **0.232** | **0.229** | **0.229** | **0.232** | **0.231** | **0.232** | **0.232** | **0.230** | **0.231** | **0.227** | **0.228** |
|  | **SD** | 0.002 | 0.001 | 0.001 | 0.002 | 0.002 | 0.003 | 0.002 | 0.001 | 0.001 | 0.002 | 0.002 | 0.003 |
|  | **Min** | 0.229 | 0.230 | 0.227 | 0.225 | 0.229 | 0.226 | 0.230 | 0.229 | 0.227 | 0.228 | 0.223 | 0.225 |
|  | **Max** | 0.237 | 0.234 | 0.231 | 0.231 | 0.234 | 0.235 | 0.234 | 0.234 | 0.232 | 0.234 | 0.230 | 0.232 |
| **DAS54** | **Av** | **0.228** | **0.229** | **0.224** | **0.225** | **0.229** | **0.227** | **0.229** | **0.229** | **0.227** | **0.229** | **0.223** | **0.223** |
|  | **SD** | 0.003 | 0.003 | 0.002 | 0.002 | 0.002 | 0.003 | 0.003 | 0.001 | 0.001 | 0.001 | 0.002 | 0.002 |
|  | **Min** | 0.224 | 0.224 | 0.221 | 0.221 | 0.225 | 0.224 | 0.225 | 0.227 | 0.225 | 0.227 | 0.219 | 0.219 |
|  | **Max** | 0.233 | 0.233 | 0.227 | 0.228 | 0.233 | 0.232 | 0.234 | 0.231 | 0.230 | 0.231 | 0.226 | 0.226 |
| **DAS70** | **Av** | **0.215** | **0.209** | **0.215** | **0.204** | **0.216** | **0.205** | **0.212** | **0.209** | **0.212** | **0.205** | **0.212** | **0.193** |
|  | **SD** | 0.007 | 0.014 | 0.004 | 0.008 | 0.004 | 0.008 | 0.008 | 0.005 | 0.005 | 0.010 | 0.004 | 0.008 |
|  | **Min** | 0.204 | 0.184 | 0.205 | 0.187 | 0.209 | 0.191 | 0.203 | 0.202 | 0.205 | 0.191 | 0.203 | 0.179 |
|  | **Max** | 0.222 | 0.221 | 0.220 | 0.213 | 0.222 | 0.216 | 0.222 | 0.217 | 0.219 | 0.216 | 0.217 | 0.204 |
| **DAS80** | **Av** | **0.204** | **0.166** | **0.198** | **0.174** | **0.198** | **0.178** | **0.205** | **0.187** | **0.209** | **0.168** | **0.191** | **0.156** |
|  | **SD** | 0.014 | 0.016 | 0.008 | 0.022 | 0.010 | 0.012 | 0.013 | 0.009 | 0.010 | 0.013 | 0.006 | 0.015 |
|  | **Min** | 0.184 | 0.132 | 0.190 | 0.136 | 0.186 | 0.154 | 0.183 | 0.175 | 0.192 | 0.146 | 0.184 | 0.132 |
|  | **Max** | 0.218 | 0.185 | 0.214 | 0.207 | 0.215 | 0.191 | 0.220 | 0.200 | 0.217 | 0.191 | 0.206 | 0.176 |
| **DAS90** | **Av** | **0.205** | **0.114** | **0.201** | **0.146** | **0.207** | **0.115** | **0.208** | **0.146** | **0.203** | **0.092** | **0.193** | **0.087** |
|  | **SD** | 0.006 | 0.025 | 0.007 | 0.028 | 0.002 | 0.029 | 0.003 | 0.032 | 0.009 | 0.020 | 0.009 | 0.013 |
|  | **Min** | 0.188 | 0.076 | 0.189 | 0.115 | 0.203 | 0.074 | 0.202 | 0.084 | 0.189 | 0.069 | 0.177 | 0.069 |
|  | **Max** | 0.214 | 0.154 | 0.210 | 0.179 | 0.210 | 0.175 | 0.211 | 0.176 | 0.212 | 0.130 | 0.203 | 0.113 |
